# Thiamine cross-feeding drives microbial coexistence in the leaf microbiome

**DOI:** 10.1038/s41564-026-02499-w

**Published:** 2026-10-05

**Authors:** Yiheng Hu, Johanna Bode, Daniel Gómez-Pérez, Marco Alexandre Guerreiro, Alfredo Mari, Kun Wang, Sinja Niemann, Maryam Mahmoudi, Ariane Kemen, Paloma Duran, Oskar Wacker, Daniel Straub, Sven Nahnsen, Benjamin Schwessinger, Fabrice Roux, Carlos Alonso-Blanco, Jon Ågren, Stéphane Hacquard, Eva H. Stukenbrock, Eric Kemen

**Affiliations:** 1 Department of Microbial Interactions, Centre of Plant Molecular Biology (ZMBP), Eberhard-Karls-University of Tübingen, Tübingen, Germany; 2 Environmental Genomics Group, Botanical Institute, Christian-Albrechts University of Kiel, Kiel, Germany; 3 Max Planck Institute for Evolutionary Biology, Plön, Germany; 4 Department of Plant Microbe Interactions, Max Planck Institute for Plant Breeding Research, Cologne, Germany; 5 Cluster of Excellence on Plant Sciences (CEPLAS), Max Planck Institute for Plant Breeding Research, Cologne, Germany; 6 Quantitative Biology Center (QBiC), University of Tübingen, Tübingen, Germany; 7 M3 Research Center, University of Tübingen, Tübingen, Germany; 8 Biomedical Data Science, Department of Computer Science, University of Tübingen, Tübingen, Germany; 9 Research School of Biology, Australian National University, Canberra, Australian Capital Territory Australia; 10 Departamento de Genética Molecular de Plantas, Centro Nacional de Biotecnología (CNB), Consejo Superior de Investigaciones Científicas (CSIC), Madrid, Spain; 11 Plant Ecology and Evolution, Department of Ecology and Genetics, EBC, Uppsala University, Uppsala, Sweden; 12 Cluster of Excellence GreenRobust, Eberhard-Karls-University of Tübingen, Tübingen, Germany; 13 Cluster of Excellence Controlling Microbes to Fight Infections, Eberhard-Karls-University of Tübingen, Tübingen, Germany; 14 Present Address: Dentistry, School of Health Sciences, College of Medicine and Health, University of Birmingham, Birmingham, UK; 15 Present Address: LIPME, INRAE, CNRS, Université de Toulouse, Castanet-Tolosan, France

**Keywords:** Microbial ecology, Microbiome, Fungal genes

## Abstract

Microbiome homeostasis is crucial for host health and ecosystem function, yet the molecular and ecological mechanisms underlying community assembly and stability remain elusive. Here we uncover a conserved yeast–oomycete association that promotes their coexistence in the leaf microbiome. Using a continental-scale microbiome survey, we identified an asymmetric mutualistic interaction between two eukaryotic hub microbes: the yeast *Dioszegia hungarica* and the obligate oomycete *Albugo laibachii*. We show that *Dioszegia* facilitates *Albugo* colonization by supplying thiamine via a dedicated membrane permease, alleviating *Albugo*’s auxotrophy. Genomic and transcriptomic analyses indicate that natural selection has acted on thiamine production in *Dioszegia*, shaping this metabolic complementation. In planta assays further suggest that *Albugo* presence supports *Dioszegia* persistence under glasshouse conditions. Our study illustrates how the evolution of nutrient cross-feeding mediates microbial coexistence and microbiome stability. Targeting microbial nutrient flows offers new strategies for engineering microbiomes and enhancing plant resilience in natural and agricultural systems.

## Abstract (web summary)

Evolution of nutrient cross-feeding between a yeast and oomycete mediates microbial coexistence and leaf microbiome stability.

## Main

Microbial communities play essential roles in shaping host performance, ecosystem function and biodiversity^1,2^. The composition of host-associated microbiomes shifts across space and time, and is influenced by environmental conditions including climate and nutrient availability, and host genetics, which together shape microbial interactions and community dynamics^3,4^. Although environmental and host factors are well established as major determinants of microbiome structure and function, which in turn can strongly influence host fitness^2,5^, the extent to which microbe–microbe interactions contribute to host-associated microbiome assembly remains unclear. This question is especially intriguing in transient ecological niches such as plant leaves, known as the phyllosphere, where seasonal fluctuations and abiotic stresses impose strong selective pressures^4^. Despite increasing efforts to characterize microbial interactions in plant leaf microbiomes^6^, the molecular mechanisms enabling coexistence, persistence and community stability in such dynamic environments are largely unresolved.

Hierarchical structures often emerge within plant-associated microbial communities, where certain microbes known as keystone or hub taxa exert a disproportionate influence on community dynamics and stability^7^. Microbial interactions, whether positive or negative, are central to this process. While negative interactions, such as competition, antagonism and amensalism, contribute to the exclusion or dominance of certain species^8^, positive interactions, such as mutualism and commensalism, are especially important for community resilience^9^. Currently, keystone species and their interaction networks are typically inferred from in silico predictions^10^. The mechanisms behind microbial interactions and their ecological functions have mostly been validated using synthetic microbial communities in vitro^6,9^. Current models of microbiome assembly in plants primarily focus on the metabolic capacities of bacteria and their resource allocations^6^. Despite these advances, it remains unclear which metabolic interactions influence the establishment or shifts in microbial hierarchies in nature, ultimately leading to a stable microbiome. To address this, systems where individual taxa strongly influence community composition offer strategic entry points.

Plant pathogens are well known for their capacity to shape the microbiome^11^. In wild *Arabidopsis*, although bacteria are considered the most abundant microbial kingdom^4^, the leaf niche can be heavily colonized by the obligate biotrophic oomycete pathogen *Albugo laibachii*, which exhibits global prevalence^12–14^. Obligate biotrophic oomycetes of the genus *Albugo* have long and complex lifecycles across diverse plant hosts and frequently establish asymptomatic infections in nature^15^. Obligate biotrophic parasites rely on living hosts for nutrients and can persist for weeks to years on their hosts^16^. This is much longer than the persistence of necrotrophic or hemi-biotrophic pathogens, which rapidly induce host death. These features suggest that *Albugo* may exert extended ecological influence within the leaf niche. Consistent with this, our previous work identified *Albugo* as a keystone taxon within the *Arabidopsis* leaf microbiome, showing a high degree of interconnectivity and a strong influence on community structure^17,18^. Therefore, the *Albugo-*centred pathosystem represents an ideal model to study the ecologically relevant mechanism of plant microbiome assembly.

The evolution of obligate biotrophy among plant pathogens is often associated with auxotrophy, that is, the loss of essential metabolic pathways such as the biosynthesis of thiamine and molybdopterin^19^, since these metabolites can be obtained from the host after successful infection. However, this process is restricted during the early infection stage, as the leaf surface is a challenging environment with limited resources^20^. Under such conditions, only a handful of stress-tolerant microbes can persist, such as the globally distributed environmental bacterium *Pseudomonas*^21^ and the yeast *Dioszegia*^22^. Whether such co-occurring microbiome members could support auxotrophic obligate biotrophs remains unknown. We hypothesized that specific positive interactions centred around these keystone taxa facilitate their niche co-adaptation and long-term persistence in planta, ultimately contributing to the hierarchical structure and stability of the broader microbial community. Such sustained positive interactions may form a mechanistic foundation for the assembly and homeostasis of stress-resilient plant microbiomes.

Here we performed a European-scale survey of wild *A. thaliana* leaf microbiomes to identify conserved microbial partners associated with *Albugo*, and uncovered a metabolically mediated mutualistic interaction that probably underpins *Albugo*-driven microbiome assembly in nature.

## Results

### A European-scale field survey identifies conserved microbial interactions in the *A. thaliana* phyllosphere

**Fig. 1:**
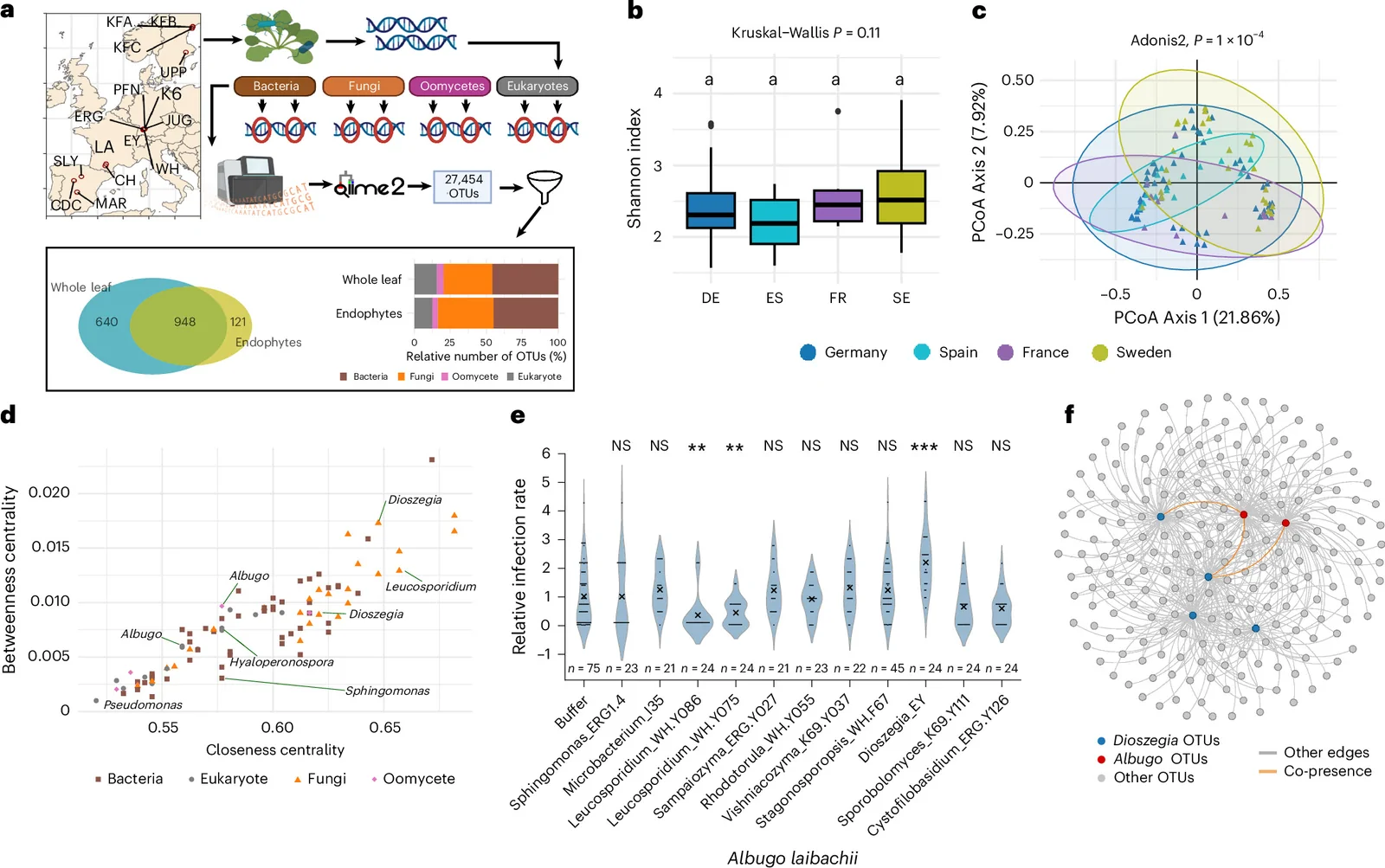
The identification of conserved *Albugo*–*Dioszegia* interactions from a European-scale survey of *A. thaliana* phyllosphere microbiome. **a**, Field sampling workflow and overview of the whole-leaf and endophyte dataset about the relative OTU numbers of each taxon group and overlapping OTUs. **b**, Alpha diversity of endophytic samples from different countries measured using the Shannon index. Boxplots show the median (centre), 25th–75th percentiles (box) and 1.5× interquartile range (IQR) (whiskers); each point is one endophytic sample from an independently field-collected *A. thaliana* plant (biological replicates; *n* = 55, 12, 26, 14 for DE, FR, SE, ES). Countries were compared using Kruskal–Wallis test (*P* = 0.11); pairwise differences were assessed using two-sided Wilcoxon rank-sum test with Hochberg correction, and shared letters denote no significant difference. **c**, PCoA based on Bray–Curtis distances between endophytic samples from different countries. Each colour represents all samples from the same country. The effect of country was tested using non-directional PERMANOVA (adonis, 10,000 permutations, *P* = 1 × 10^−^^4^). A single omnibus test was performed across the four countries, so no multiple-comparison adjustment was required. **d**, Measures of closeness centrality and betweenness centrality of the endophyte OTUs. Only OTUs present in at least 10 samples and containing a minimum of 50 reads were retained for downstream analyses. OTUs known to be part of the *A. thaliana* core microbiome (for example, *Pseudomonas*, *Sphingomonas*) are labelled as reference points to validate network structure, while focal hub taxa (*Albugo*, *Dioszegia*) are highlighted on the basis of their high centrality rankings across sites. **e**, Violin plot showing *Albugo laibachii* infection rate relative to the buffer control when co-inoculated with different interactor strains (mainly basidiomycete yeasts). The common environmental yeasts *Rhodotorula* and *Cystofilobasidium* were not among the interactors and were used as negative controls. The infection rate of each group was calculated on the basis of the average number of infected leaves per plant at 12 days after *Albugo* inoculation (dai; Methods) and normalized to the buffer control of each experiment. Each dot represents one plant, and the cross in the violin plots represents the mean of each dataset. *P* values were determined using two-sided Mann–Whitney *U*-test between each treatment group and the buffer control; ***P < 0.001, **P < 0.01, ^NS^*P* ≥ 0.05. **f**, Co-abundance network subset from the whole-leaf network, containing only *Albugo* and *Dioszegia* OTU nodes and their direct interactors. Red and blue nodes represent *Albugo* and *Dioszegia* OTUs, respectively; orange edges between them represent positive interactions based on co-presence in the network. Panel **a** created in BioRender; Kemen, E. https://BioRender.com/e2ahb4h (2026). Source data

**Extended Data Fig. 1:**
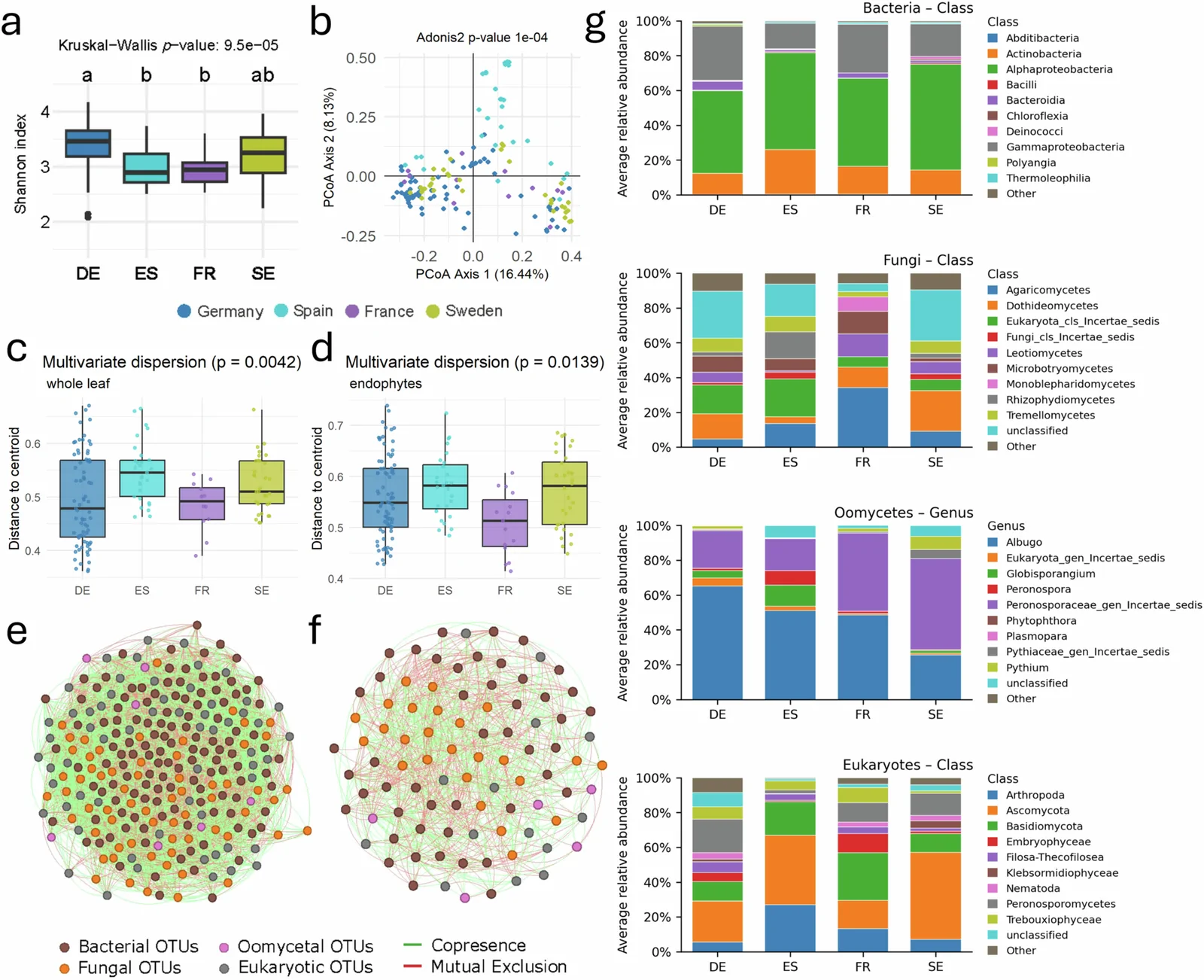
Supporting evidence to the identification of *Albugo*–*Dioszegia* interactions. (**a**) α-diversity measures of whole leaf samples from different countries represented by Shannon index. Each point represents one independently field-collected whole-leaf sample (biological replicates; n = 75, 29, 12 and 33 for DE, ES, FR and SE). Letters depict significant differences across countries (*p* = 9.5×10⁻^5^, Kruskal–Wallis test; pairwise comparisons by two-sided Wilcoxon rank-sum tests with Hochberg correction). (**b**) Principal coordinate analysis (PCoA) based on Bray–Curtis distances between whole leaf samples from different countries. Each color represents all samples from the same country. The effect of country was tested by the non-directional PERMANOVA (adonis, 10,000 permutations, *p* = 1×10⁻^4^). (**c**) and (**d**) Box plots illustrating the multivariate dispersion (measured via betadisper) for the whole leaf (**c**) and endophytic (**d**) microbial communities across countries. The y-axis shows the distance of individual samples to their respective group centroids in multivariate space. *p*-values are derived from permutation tests of significance (10,000 permutations). Sample sizes are the same as in Extended Data Fig. 1a for (**c**) and Fig. 1b for (**d**). In (**a**), (**c**) and (**d**), box plots show the median (center line), the 25th–75th percentiles (box) and whiskers extending to the most extreme values within 1.5× the interquartile range; points beyond the whiskers are outliers. (**e**) whole leaf network and (**f**) endophyte network. Node and edge sizes are unscaled. The edge color represents the type of interaction between two nodes as indicated. (**g**) Relative abundance of the 10 most abundant taxa for each kingdom in whole leaf samples. Bacteria, fungi, and eukaryotes are represented at class level, oomycetes are represented at genus for better illustration since the total number of oomycete OTUs are lower compared to other kingdoms.

To identify conserved interactions in the *A. thaliana* phyllosphere microbiome, we conducted a continent-scale survey across western, middle and northern Europe to capture the maximum microbial diversity. We collected 347 plants from 15 sites harbouring natural *A. thaliana* populations in Spain, France, Germany and Sweden (Fig. 1a). We performed marker gene amplicon sequencing on bacteria (16S), fungi (ITS2), oomycete (ITS1) and other eukaryotes (18S), using half of the plant material as ‘whole-leaf microbiota’ samples and surface sterilizing the other half to obtain the endophytic microbiota. Since species-level resolution was not consistently achievable for ITS and 18S markers, and because our goal was to identify conserved ecological structure, we used operational taxonomic unit (OTU) clustering as a harmonized framework for multikingdom analysis. On the basis of OTU classification, we found that microbial alpha diversities (Shannon index) were comparable across different sampling sites (Fig. 1b for endophytes and Extended Data Fig. 1a for whole leaf). However, beta-diversity analyses, including principal coordinates analysis (PCoA) and multivariate dispersion tests, revealed significant differences in microbial community composition and within-region variation across the four geographical regions (Fig. 1c and Extended Data Fig. 1b–d). These results align with previous reports of host biogeography explaining microbial variation^8,23^, and provide a diverse ecological context for distinguishing universal microbial hubs from site-specific variation. On the basis of the composition of the microbiome in whole leaf samples (Extended Data Fig. 1g), we found the most abundant taxa to be Alphaproteobacteria among bacteria (53.6%), Agaricomycetes among fungi (15.4%), the genus *Albugo* among oomycetes (57.8%) and Peronosporomycetes among eukaryote microbes (40.3%).

We next investigated the intra- and interkingdom microbial OTU relationships using the network inference tool Fastspar^24^. Among the major taxa that were primarily shared across different sites (present in >10 samples, containing a minimum of 50 reads), we observed a similar number of positive and negative correlations from the whole-leaf microbiome (4,457 positive and 4,075 negative, Extended Data Fig. 1e). To reduce the complexity and focus on the microbes that form tight associations with *A. thaliana*^25^, we further constructed a network of endophytes (Extended Data Fig. 1f). To assess the role of each microbial OTU in the community, we calculated closeness centrality, quantifying how easily a microbe can reach other microbes within the network, and betweenness centrality, reflecting its bridging influence as a connector in the network^26^. Among the most conserved OTUs in endophytic samples, we identified microbial OTUs from genera that are commonly found in the *Arabidopsis* leaf microbiome such as the bacterial genera *Pseudomonas* and *Sphingomonas*, and the oomycetal genus *Hyaloperonospora*^14,27^ (Fig. 1d). We also observed that several OTUs from basidiomycete yeasts *Dioszegia* and *Leucosporidium* displayed outstanding betweenness centrality among all OTUs, indicating their connecting role within the microbiome. Across all samples, two OTUs classified as *Albugo* appeared to be among the most prominent hub taxa among leaf-resident oomycete and eukaryote OTUs, as reflected by their high rankings in both closeness centrality and betweenness centrality measures of OTUs within their group (Fig. 1d). Given its global prevalence and capacity for asymptomatic persistence in the phyllosphere^12–15,28^, our result validated the central organizing positions of *Albugo* within the *A. thaliana* leaf microbiome.

### A basidiomycete yeast *Dioszegia* strain promotes the colonization of *A. thaliana* by *A. laibachii* under gnotobiotic conditions

**Extended Data Fig. 2:**
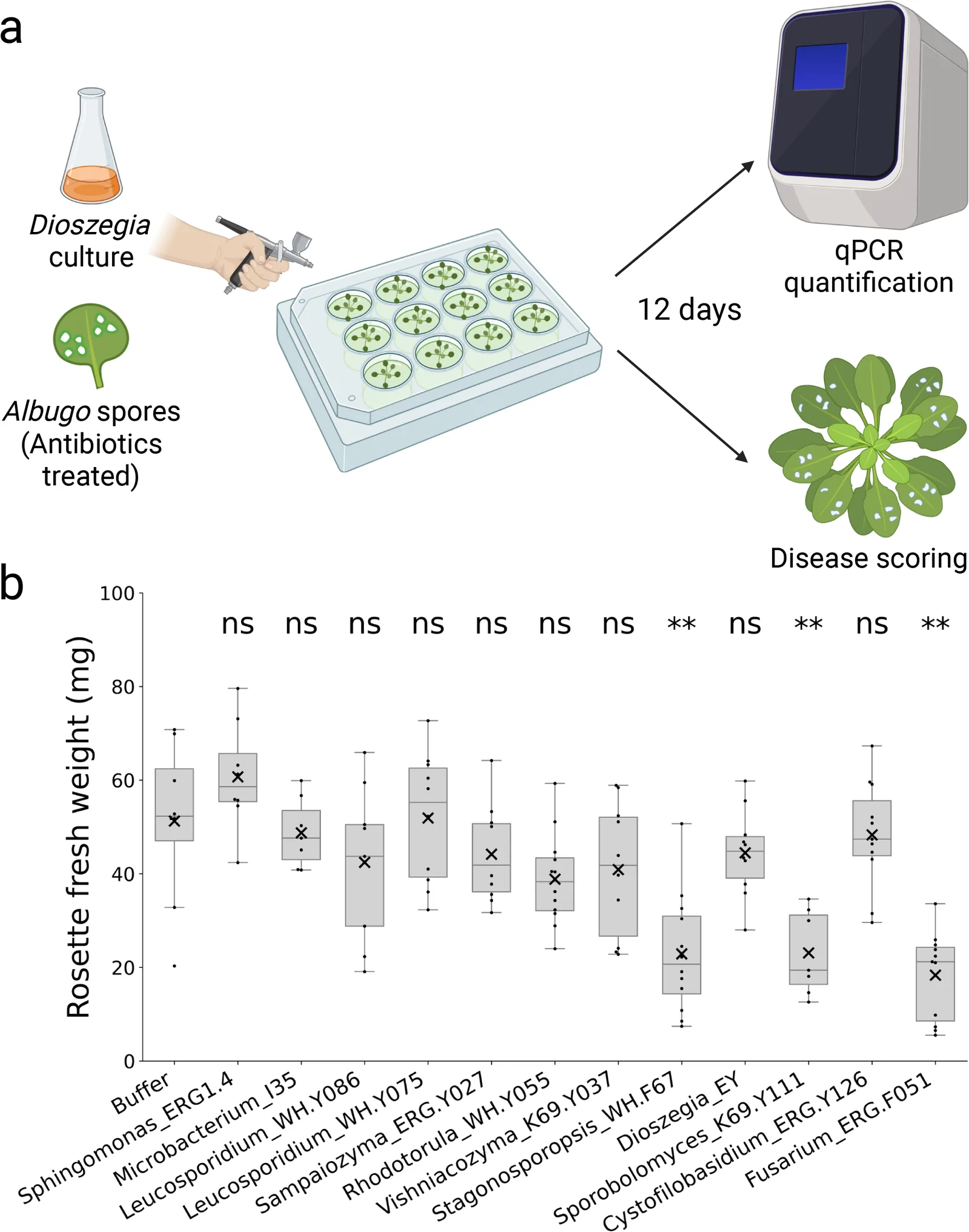
The gnotobiotic system for testing the effect of microbes on the colonization of *A. laibachii*. (**a**) Workflow of the gnotobiotic co-cultivation of *Albugo* with different leaf associated microbes. The disease scoring is counting the average number of infected leaves for each plant. This figure was generated using BioRender. (**b**) *Arabidopsis* biomass measured by rosette fresh weight 12 days after inoculation of different microbes. Seedlings are inoculated at 4 weeks old. Each dot represents one independently inoculated plant (biological replicates); from left to right n = 8, 8, 7, 9, 10, 10, 12, 10, 12, 10, 7, 11 and 11 plants (125 in total). Box plots show the median (center line), the 25th-75th percentiles (box) and whiskers extending to the most extreme values within 1.5x the interquartile range; × marks the group mean. Asterisks or ns represent the *p* value from a two-sided Mann-Whitney U test between each treatment group and the buffer control: *Stagonosporopsis*, **p = 0.0023; *Sporobolomyces*, **p = 0.0065; *Fusarium*, **p = 0.0034; all other treatments, ns (*p* > 0.05). Source data

Given *Albugo’s* central role in the microbial network, we hypothesize that its direct interactions with other microbes play a key role in the assembly of the *Arabidopsis* leaf microbiome. To investigate this, we ranked both predicted positive and negative interactions involving *Albugo* OTUs in the whole-leaf samples based on closeness and betweenness centrality measures (Supplementary Table 1), and attempted to verify these interactions experimentally to unravel the molecular mechanisms of *Albugo-*centred microbial interactions. We adapted a previous gnotobiotic system using sterile *A. thaliana* grown on 12-well plates to screen and quantify the effect of single phyllosphere microbes on the colonization of *Albugo laibachii*. We assessed colonization success on the basis of the number of infected leaves per plant and quantitative PCR (qPCR) quantification of *Albugo* biomass (Extended Data Fig. 2a). Using this system, we tested 12 potential *Albugo* interactors. These interactors were identified from our inferred network and correspond to taxa that had been previously isolated from wild *A. thaliana* near Tübingen (48.45° N, 8.78° E). We found that most of the *Albugo*-interacting microbes were either pathogenic to *A. thaliana* or affected neither *A. laibachii* infection nor *Arabidopsis* biomass (Fig. 1e and Extended Data Fig. 2b). In addition, an OTU classified as the basidiomycete yeast *Leucosporidium* was predicted to form a strong positive interaction with *Albugo* (Supplementary Table 1), but two different strains of this genus significantly inhibited *Albugo* infection under gnotobiotic conditions (Fig. 1e). This result underscores the challenge in translating in silico microbial associations into direct functional outcomes^29^. While network centrality metrics identify hub taxa from thousands of OTUs, inferred microbial interactions can be context dependent and may require additional environmental or microbial factors to manifest in simplified pairwise assays^30^.

In contrast, a *Dioszegia hungarica* strain EY consistently showed a positive interaction with *A. laibachii*, aligning with in silico predictions (Fig. 1e). The basidiomycete yeast *Dioszegia* was identified as a major hub taxon in the *A. thaliana* phyllosphere microbiome in our previous study^17^ and was among the most interconnected fungal OTUs, with the second highest betweenness centrality in the present study (Fig. 1d). To validate the interconnectivity of all *Dioszegia* and *Albugo* OTUs as hub taxa and to establish their interactions, we extracted all direct interactions of *Albugo* and *Dioszegia* OTUs from the whole-leaf network (Fig. 1f). We found three direct connections between *Albugo* and *Dioszegia* OTUs, with additional indirect links through other microbes. Both taxa maintained extensive connections to the broader community, reinforcing their positions as central hubs. The results from both in silico and in planta experiments thus suggest that the positive interaction between *Albugo* and *Dioszegia* represents a key microbial interaction in the phyllosphere microbiome of different *A. thaliana* populations.

### Comparative genomics identifies a *D. hungarica* thiamine permease that is crucial for the *Dioszegia*–*Albugo* interaction

**Fig. 2:**
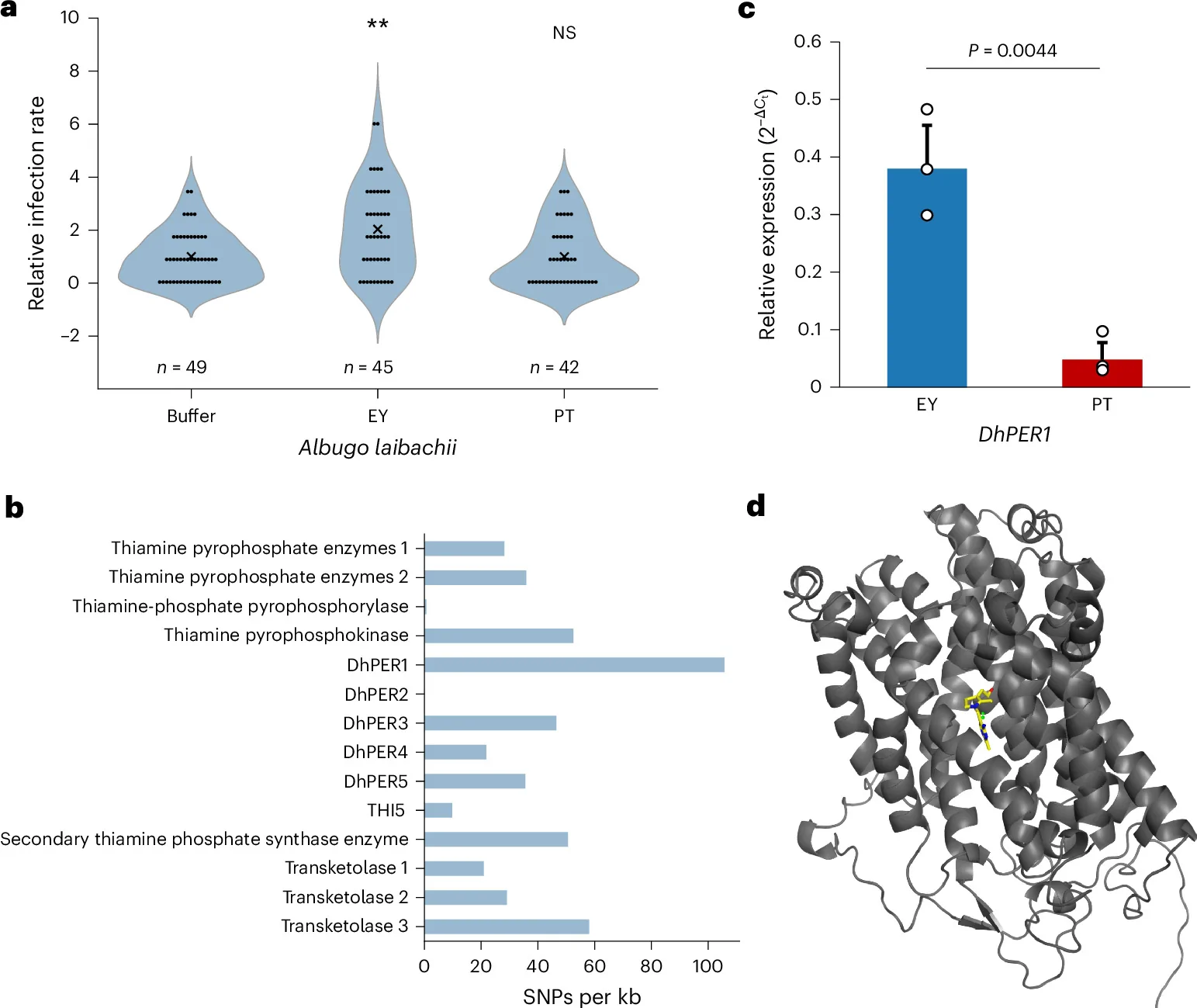
A thiamine permease is differentially expressed in *A. laibachii*-promoting and non-promoting strains of *Dioszegia*. **a**, Violin plot showing the infection rate (number of infected leaves per plant at 12 dai) of *A. laibachii* in the presence of either *Dioszegia* EY or PT as normalized to buffer control. Each dot represents one plant, and the cross represents the mean of each dataset. *P* values were determined using two-sided Mann–Whitney *U*-test between each treatment group and the buffer control; **P = 0.0018, ^NS^*P* = 0.69. This experiment was replicated 6 times and the same result was observed. **b**, Barplot illustrating the variations in coding sequences (CDS) of all thiamine metabolism genes between PT and EY. The variations are shown as SNPs per kilobasepair. **c**, Quantitative PCR with reverse transcription (RT–qPCR) results measuring the relative expression of *DhPER1* between EY and PT in vitro. The expression of *DhPER1* is normalized to the expression of *D. hungarica* elongation factor 1α (*EF1α*) gene. Each data point was calculated from the median of 3 technical replicates. Bars show the mean and error bars represent the 95% confidence interval of the mean. *P* values were calculated using two-sided Student’s *t*-test. **d**, Molecular docking of the thiamine-HCl compound with *DhPER1* (grey colour in ribbon representation) from *Dioszegia* EY using DiffDock (https://github.com/gcorso/DiffDock). Source data

**Extended Data Fig. 3:**
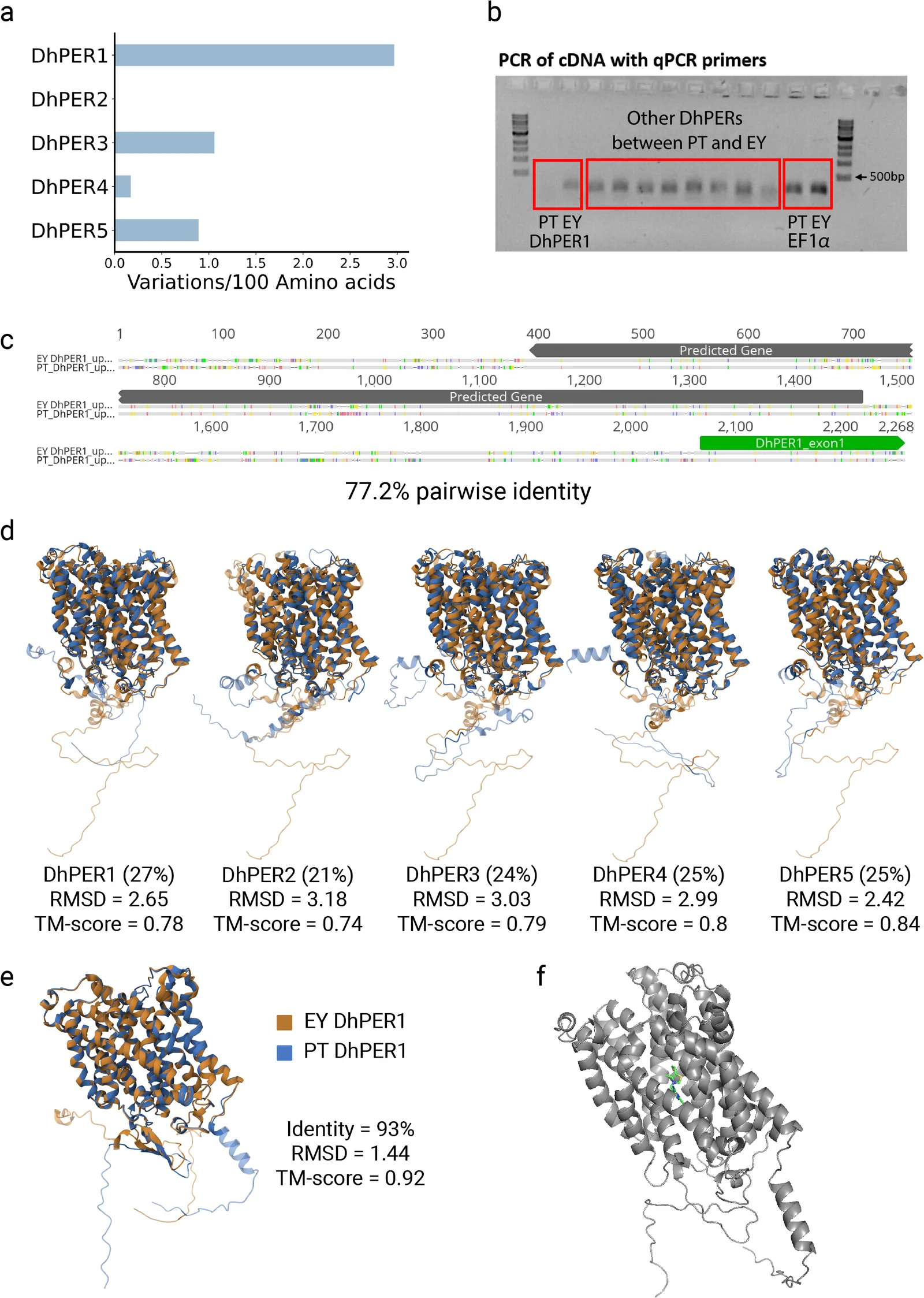
Validation of *DhPER1* as the candidate gene responsible for the Albugo promotion. (**a**) Measurement of pairwise variations per 100 amino acids for predicted thiamine permeases of *Dioszegia* EY compared to PT. *DhPER1* showed the highest variation. (**b**) Agarose gel electrophoresis of RT-PCR for five *DhPERs in vitro* to assess the expression of the *DhPERs*. *DhPER1* showed expression in EY but not in PT, and all other *DhPERs* were expressed in both strains. *EF1a* gene was used as a positive control of the PCR experiment. For each gene, PT and EY were loaded in adjacent lanes, and a DNA size ladder was run on both sides of the gel with the 500 bp band indicated (arrow). This experiment was repeated twice with similar results. (**c**) Pairwise comparison of the 2 kb upstream region of *Dioszegia* EY and PT *DhPER1* genes. The different colors in the alignment indicate the polymorphic nucleotide positions, the green label represents the position of the first exon from *DhPER1*, and the grey label annotates a predicted gene within the 2 kb region upstream of *DhPER1*. Both labels are based on gene predictions from EY strain. (**d**) Alignment of the Alphafold2-predicted structures of different *DhPERs* with that of *THI7* from *Saccharomyces cerevisiae* (UniProt A7A1C0_YEAS7) in ribbon representation. *DhPERs* are in orange and *THI7* is in blue color. (**e**) Alignment of the Alphafold2-predicted structures of *Dioszegia* EY and PT *DhPER1* in ribbon representation. (**f**) Molecular docking of the thiamine-HCl compound with *DhPER1* (grey color in ribbon representation) from *Dioszegia* PT using DiffDock (https://github.com/gcorso/DiffDock). The confidence score is -1.6. Source data

We hypothesized that the metabolic auxotrophy of *Albugo* may be complemented by positive interactors such as *Dioszegia*. We employed comparative genomics to identify genetic differences between *Dioszegia* strains that promote and those that do not promote *Albugo*, focusing on essential metabolic pathways absent in *Albugo*. We first obtained nine strains of *Dioszegia* from the fungal collection centre in the Netherlands (Supplementary Table 2) and tested their influence on *A. laibachii* infection. We identified another *D. hungarica* strain CBS10163 (PT), originating from maple leaves in Portugal, which did not promote *A. laibachii* infection (Fig. 2a). We next generated draft genomes of *Dioszegia* EY and PT strains using a recent ref.^31^ (Supplementary Table 3) and compared all their thiamine-related genes. We focused on this biosynthetic pathway because it is absent in the *Albugo* genome, making thiamine a primary candidate for metabolic complementation in the nutrient-limited phyllosphere. We found 7 functional groups for a total of 14 thiamine-related genes in each genome and manually curated them by PCR with reverse transcription (RT–PCR) and Sanger sequencing. To characterize the genetic variation of these 14 genes, we compared the number and distribution of the single-nucleotide polymorphisms (SNPs) for each thiamine-related gene between *Dioszegia* EY and PT strains. We observed that one gene, *Dioszegia hungarica* PERMEASE 1 (*DhPER1*), has a high density of SNPs (105.7 SNPs per kb) relative to the other thiamine-related genes in EY and PT genomes (Fig. 2b), nearly double that of the second highest genes. *DhPER1* also carries the highest amino acid variation of all 5 permease genes (Extended Data Fig. 3a), possibly resulting in functional diversification.

We also inspected in vitro transcriptional differences related to thiamine metabolism between the two strains in vitro. *DhPER1* expression was more than 20-fold higher in *Dioszegia* EY than in PT (Fig. 2c) and was the only thiamine-related gene with negligible expression in PT (Extended Data Fig. 3b). The expression difference might be related to high levels of nucleotide diversity in the extended promoter region (2 kb upstream) of the coding sequence (Extended Data Fig. 3c), suggesting that this expression difference is due to *DhPER1 cis*- and/or *trans*-regulatory mutations. To determine whether *DhPER1* is the primary permease for thiamine, we predicted the protein structures of all 5 permeases and aligned them with the predicted structure of a known thiamine transporter, *THI7*, from *Saccharomyces cerevisiae*^32^ (Extended Data Fig. 3d). Based on three structural similarity metrics—percentage identity, root mean square deviation (RMSD) and template modelling (TM) score—DhPER1 was one of the two closest matches to THI7. We also performed molecular docking of DhPER1 with thiamine-HCl, the transportable form of thiamine, and thiamine-HCl anchored well with the predicted protein (Fig. 2d, confidence = −0.92). Comparison of *DhPER1* protein structure and molecular docking with thiamine-HCl between EY- and PT-predicted proteins did not show notable structural differences (Extended Data Fig. 3e,f). Therefore, although both structural and regulatory polymorphisms in *DhPER1* might affect its function, the low predicted impact of missense mutations and the near absence of expression in *Dioszegia* PT suggest that the higher expression of *DhPER1* in *Dioszegia* EY underlies its beneficial effect on *Albugo* colonization of *A. thaliana* leaves.

### The *DhPER1*^EY^ is responsible for thiamine-mediated *A. laibachii* promotion in planta

**Fig. 3:**
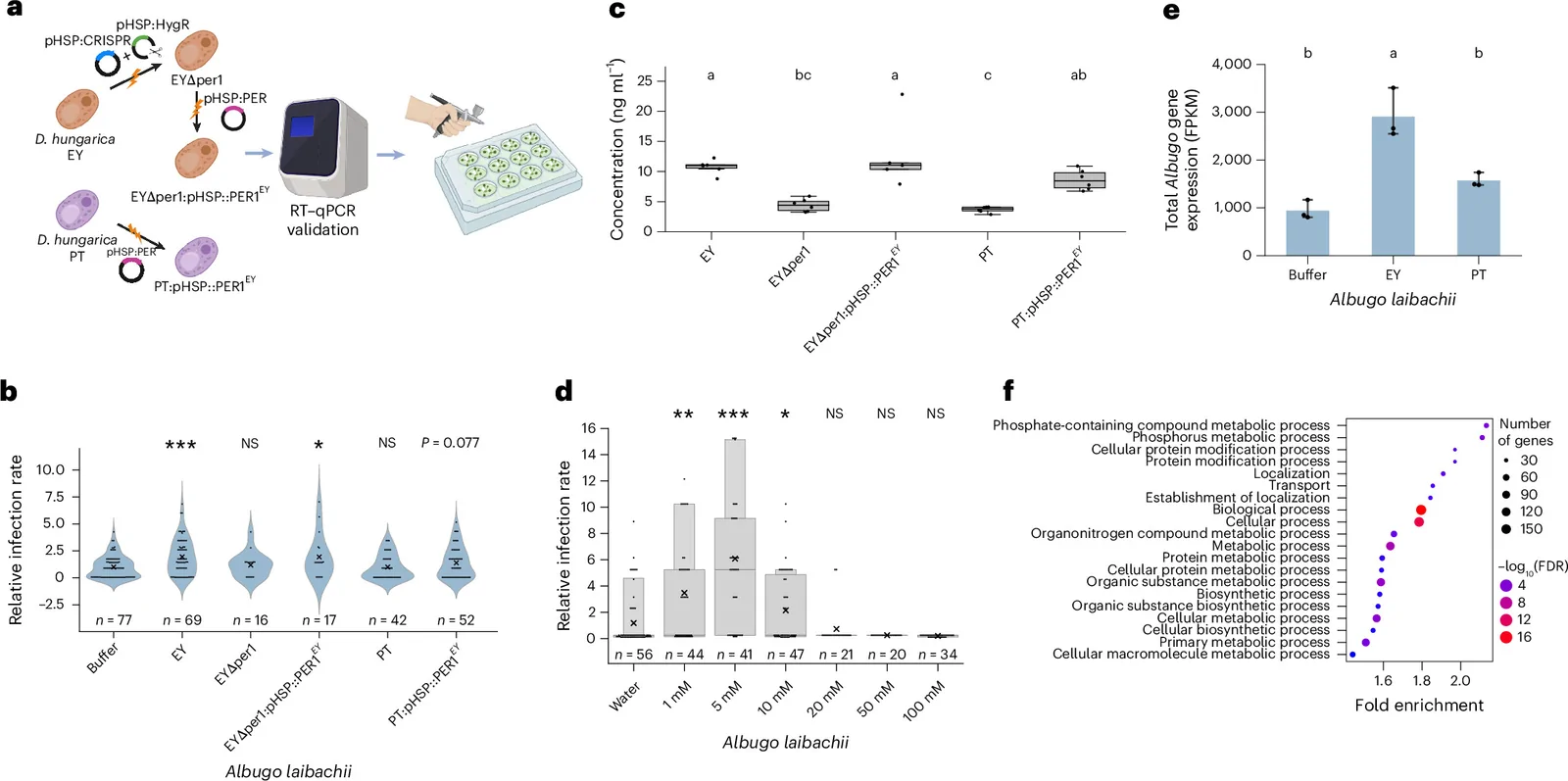
Positive feedback cycle between *Dioszegia* EY and *Albugo* depending on *DhPER1*. **a**, Overview of the strategy for the genetic modification of *Dioszegia* EY and PT strains. The expression of *DhPER1*^EY^ was first validated in all five strains depicted through RT–qPCR, followed by in planta testing using the gnotobiotic infection system. **b**, Violin plot showing the infection rate of *A. laibachii* in the presence of different *Dioszegia* EY and PT strains normalized to the buffer control. EYΔper1:pHSP::PER1^EY^ represents the complementation strain of the EYΔper1 mutant, and PT:pHSP::PER1^EY^ represents the PT complementation strain with overexpressing *DhPER1* gene of EY. Each dot represents one plant, and the cross represents the mean of each dataset. *P* values were calculated using two-sided Student’s *t*-test between each treatment group and the buffer control; ***P = 4.4 × 10^−^^5^, *P = 0.0064, ^NS^*P* ≥ 0.05. **c**, Boxplot showing the extracellular thiamine concentration determined by ELISA. Each dot represents a single biological replicate, and the box represents the middle 50% of the data. The centre line represents the median and the whiskers extend to the most extreme values within 1.5× IQR. All groups contain 6 biological replicates, except EYΔper1:pHSP::PER1 containing 5 due to a mistake during the ELISA sample preparation. Different letters represent significant differences based on two-sided Tukey’s HSD test results (*P* < 0.05). The raw absorbance in PT and EYΔper1 was lower than the detection limit, and was calculated on the basis of the standard curve. **d**, Boxen plot showing the effect of different concentrations of thiamine on *A. laibachii* infection, quantified by the relative infection rate. The bottom dark grey boxes represent the 50% percentile and the upper light grey boxes represent the 75% percentile. *P* values were calculated using two-sided Mann–Whitney *U*-test between each treatment group and the buffer control; ***P = 2.8 × 10^−^^8^, **P = 0.001, *P = 0.036, ^NS^*P* ≥ 0.05. The cross in **b** and **d** represent the mean of each dataset. **e**, The total expression patterns of *A. laibachii* at 2 dai measured by FPKM. The samples are from sterile plants sprayed with *A. laibachii* and *Dioszegia* EY or PT, and harvested at 2 dai. Bars show the mean, error bars represent the 95% confidence interval of the mean, and dots show individual biological replicates (*n* = 3). The different letters represent significant differences from two-sided Tukey’s HSD test results (buffer versus EY, *P* < 0.001; EY versus PT, *P* = 0.0066; buffer versus PT, *P* = 0.13). **f**, Dotplots showing the term enrichment (GO biological process) analysis on all *A. laibachii* DEGs upregulated by *D. hungarica*. The top 20 enriched GO terms are displayed, ranked by fold enrichment. Panel **a** created in BioRender; Kremen, E. https://BioRender.com/ydyuyre (2026). Source data

**Extended Data Fig. 4:**
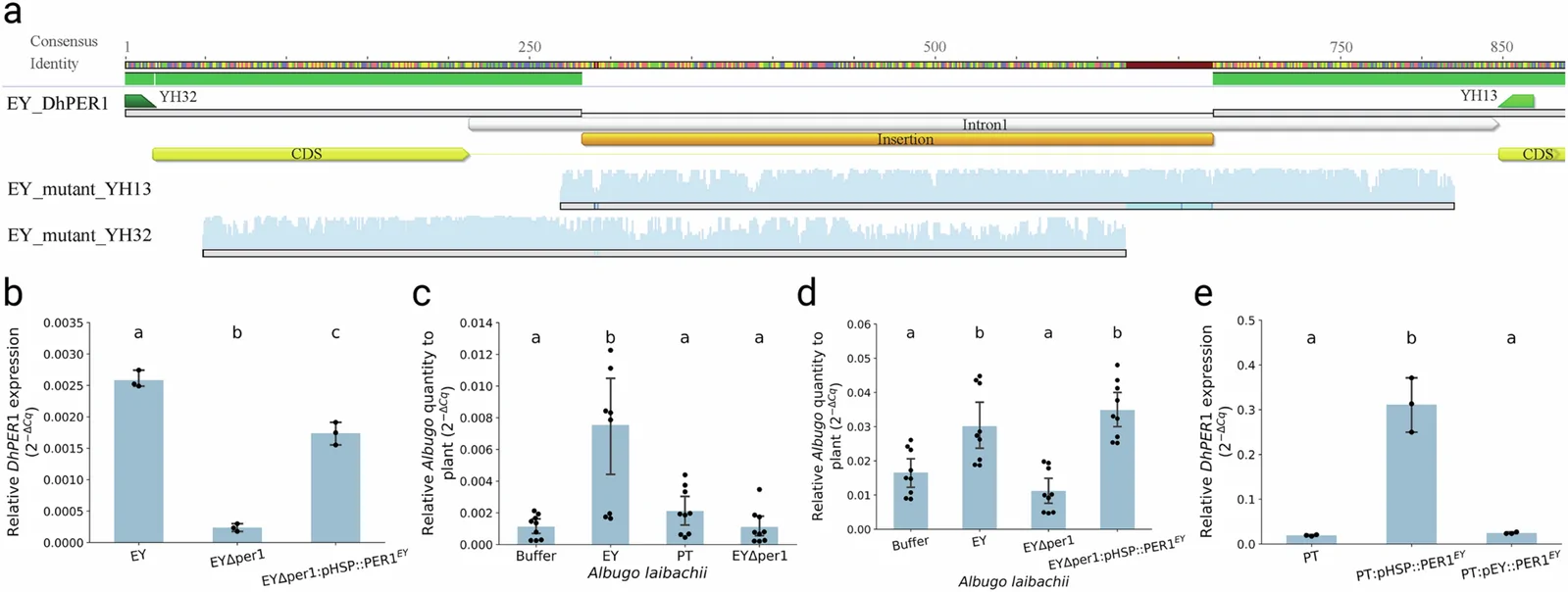
Validation of *DhPER1*^EY^ knock-out and complementation mutants. (**a**) Sanger sequencing validation of the 389 basepair insertion in EYΔper1 mutant. The *DhPER1*^EY^ gene was used as reference and the sequencing primers are annotated. Forward and reverse reads were aligned to the reference, with light blue peaks indicating the quality measure of each base. The insertion is highlighted in orange. (**b**) Quantitative RT-qPCR validation of the *DhPER1* expression in *Dioszegia* EY mutants and complementation strains. The mutant was generated using the pHSP:CRISPR and pHSP:Hyg vectors, and the complementation strain by transforming the pHSP:PER vector into the EYΔper1 background. The *D. hungarica EF1a* gene was used as reference (n = 3 per strain). (**c**) and (**d**) qPCR quantification of *A. laibachii* biomass normalized to *A. thaliana* biomass. In both panels each data point is an independent biological replicate consisting of tissue pooled from 5-12 plants, extracted and measured separately (n = 9 per group). Three experiments were conducted (see Methods). The data in (**d**) correspond to the scoring data from Fig. 3b. (**e**) Quantitative RT-qPCR validation of the *DhPER1* expression in *Dioszegia* PT complementation strains. The PT:pHSP::PER1^EY^ line was transformed using the pHSP:PER vector and the PT:pEY::PER1^EY^ line using the pEY:PER vector, both in the PT background (n = 3 per strain). In (**b**) to (**e**), bars show the mean, error bars represent the 95% confidence interval of the mean, and dots show the individual biological replicates. Different letters denote significant differences between groups from two-sided Tukey’s HSD tests with a family-wise error rate of 0.05. Exact *p* value of each comparison is listed in the Source Data. Source data

We sought to confirm the genetic and functional importance of *DhPER1* in *A. laibachii* promotion. We developed an electroporation-based transformation and CRISPR system for the *Dioszegia* EY strain (Fig. 3a). We identified a mutant (EYΔper1) with a 389 bp insertion in the first intron of *DhPER1*, which abolished the expression of *DhPER1*^EY^ (Extended Data Fig. 4a,b). We complemented the EYΔper1 mutant and subsequently tested the mutant and complemented strains in the gnotobiotic system with *A. laibachii*. Both phenotyping and qPCR analyses revealed that the EYΔper1 mutant had lost its capacity to promote *Albugo* infection, while complementation of the mutant with *DhPER1*^EY^ under a heterologous overexpression promoter restored the phenotype (Fig. 3b and Extended Data Fig. 4c,d). We also ectopically expressed *DhPER1*^EY^ in the PT strain using the same overexpression promoter (Extended Data Fig. 4e). We found that PT overexpressing *DhPER1*^EY^ facilitated *Albugo* infection slightly, but the effect was not statistically significant (Student’s *t*-test, *P* = 0.077, Fig. 3b). This result suggests that the differential expression of *DhPER1*^EY^ between *Dioszegia* EY and PT strains is necessary but not sufficient to fully promote *Albugo* infection. We also transformed PT with *DhPER1*^EY^ under EY’s endogenous promoter, and the transformant did not increase *DhPER1* expression (Extended Data Fig. 4e). Hence, additional *trans* regulation on *DhPER1* expression might also differ between *Dioszegia* EY and PT strains. Together, these results indicate that *DhPER1* is a key *Dioszegia* gene responsible for *Albugo* promotion, and that the *Dioszegia* PT strain lacks multiple genetic components of the *DhPER1* pathway mediating the facilitation of *Albugo* infection.

Next, we attempted to validate the metabolic and physical basis of interactions between *Dioszegia* and *Albugo*. We first assessed the capacity of thiamine exportation in different *Dioszegia* strains with an enzyme-linked immunosorbent assay (ELISA) in vitro. In the thiamine-free yeast nitrogen base medium, all strains expressing *DhPER1*^EY^ exhibited higher extracellular thiamine levels (Fig. 3c), demonstrating that DhPER1 mediates the secretion of thiamine. To test whether thiamine directly facilitates *Albugo* colonization, we applied different concentrations of thiamine together with *A. laibachii* in the gnotobiotic system^33^. We found that low concentrations of thiamine increased the success of *A. laibachii* infection by up to 500% (at 5 mM, Mann–Whitney *U*-test *P* = 2.8 × 10^−08^) as compared to the buffer control (Fig. 3d). The promotion effect sharply dropped when the thiamine concentration was higher than 10 mM, and thiamine became lethal to the plants at 20 mM and higher concentrations (Fig. 3d). To test whether thiamine-mediated infection promotion resulted from direct *Dioszegia*–*Albugo* interaction, we applied *A. laibachii* spores on *Dioszegia* EY cell clumps and tracked the movement of hatched zoospores under the microscope. We observed that once *A. laibachii* zoospores were physically close to *Dioszegia* EY cells, they moved in the direction of EY cells instead of moving freely in water (Supplementary Videos 1–6). These results support the hypothesis that *Dioszegia* EY attracts *A. laibachii* zoospores and cross-feeds thiamine to the zoospores via physical contact.

**Extended Data Fig. 5:**
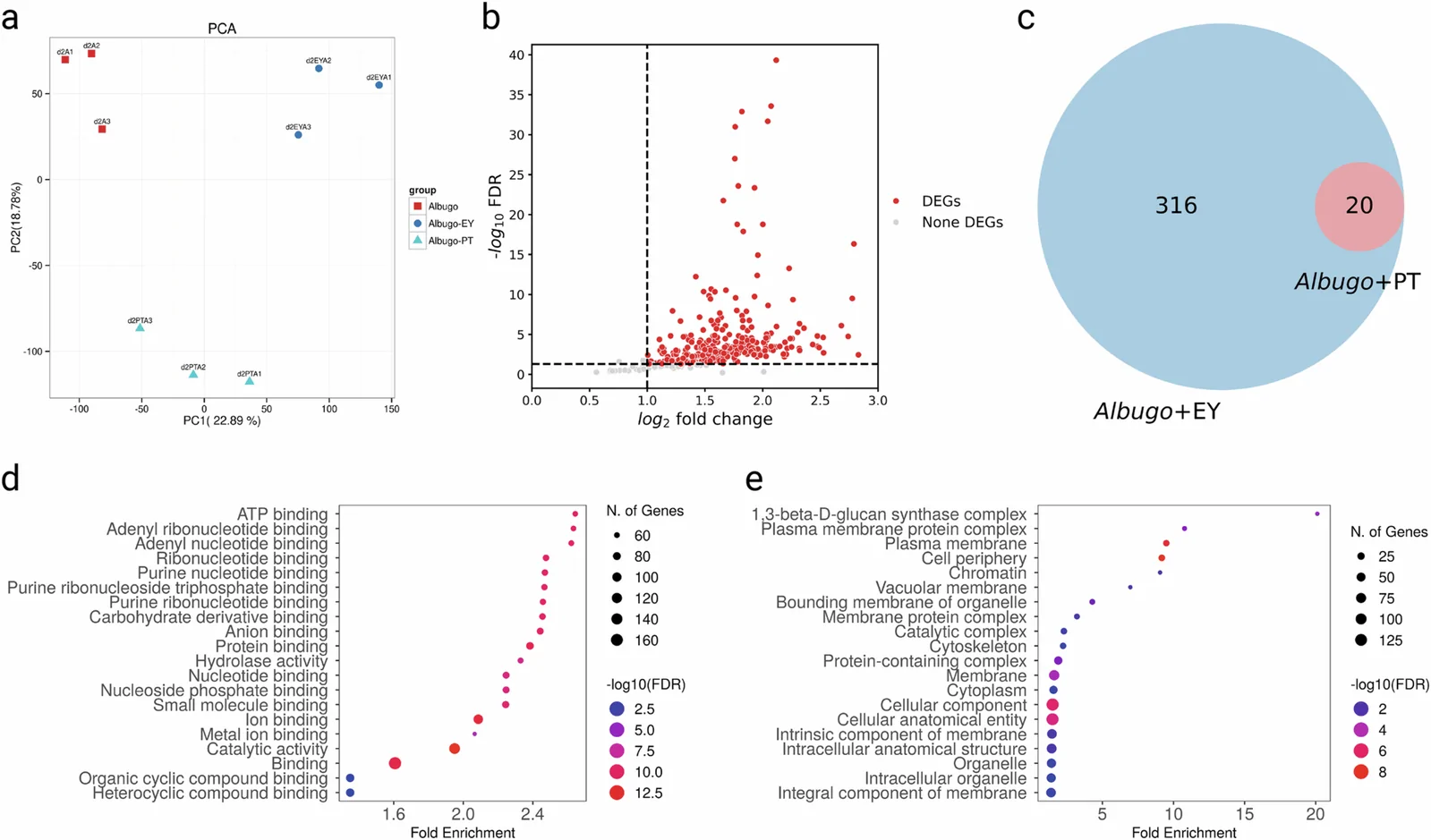
RNA-seq analysis of 2 dai *A. laibachii*-infected plants with *Dioszegia* EY and PT. (**a**) Principal component analysis(PCA) on FPKM of each sample from the RNA-seq experiment. Similarity among samples was displayed by reducing dimensionality into two principal components. (**b**) Volcano plot of all *A. laibachii* DEGs in the *Dioszegia* EY treated groups compared to buffer control. Red color represents all significant differential expression with |log_2_FC|> 1 and FDR < 0.05 cut-off. (**c**) Venn diagram comparing the upregulated *A. laibachii* genes induced by *Dioszegia* EY and PT. (**d**) and (**e**) Dot plots showing the term enrichment analysis of GO: Molecular Function (**d**) and GO: Cellular Component (e) on all *A. laibachii* DEGs upregulated by *Dioszegia*. Top 20 enriched GO terms displayed, ranked by fold enrichment.

The release of zoospores is the very first step of oomycete infection^34^. To verify our hypothesis, we used an RNA-seq approach to test whether adding *Dioszegia* would promote the infectivity of *Albugo* zoospores. We sprayed *A. laibachii* with either *Dioszegia* EY or PT, or a buffer-only control on sterile *A. thaliana* leaves, and collected samples for total RNA-seq 2 days after infection (dai). Sequencing produced mixed plant–microbial transcriptomes, which we mapped simultaneously to the *Arabidopsis* and *Albugo* reference transcriptomes. Reads assigned to the *Albugo* transcriptome were extracted to quantify pathogen activity using fragments per kilobase per million mapped reads (FPKM). Total *Albugo* expression increased 3-fold in the presence of *Dioszegia* EY compared to the buffer control (Tukey’s HSD test *P* < 0.001), whereas *Dioszegia* PT had no significant effect (Fig. 3e). A principal coordinates analysis of the gene expression data revealed distinct expression patterns among the three groups (Extended Data Fig. 5a). Using |log_2_FC| ≥ 1 and false discovery rate (FDR) < 0.05 cut-offs, we identified 316 differentially expressed genes (DEGs) in *A. laibachii* when infected plants were sprayed with *Dioszegia* EY compared to the buffer control, all of which were upregulated (Extended Data Fig. 5b). In contrast, PT treatment upregulated only 20 *A. laibachii* genes, all of which were also present in the EY-induced DEGs (Extended Data Fig. 5c). Gene ontology (GO) term enrichment analysis of the 316 upregulated *A. laibachii* genes showed an overrepresentation of terms related to metabolic processes and membrane transport (Fig. 3f and Extended Data Fig. 5d,e). Thus, the transcriptome analysis showed that *Dioszegia* EY treatment induced *Albugo* metabolism during the early stage of infection, facilitating initial colonization of *A. thaliana* by *Albugo*. Together, our results demonstrate that *DhPER1*^EY^ mediates thiamine cross-feeding from *Dioszegia* EY to *A. laibachii*, supporting its establishment.

### Genetic rearrangements and translational optimization at the *Dioszegia PER1* locus

**Fig. 4:**
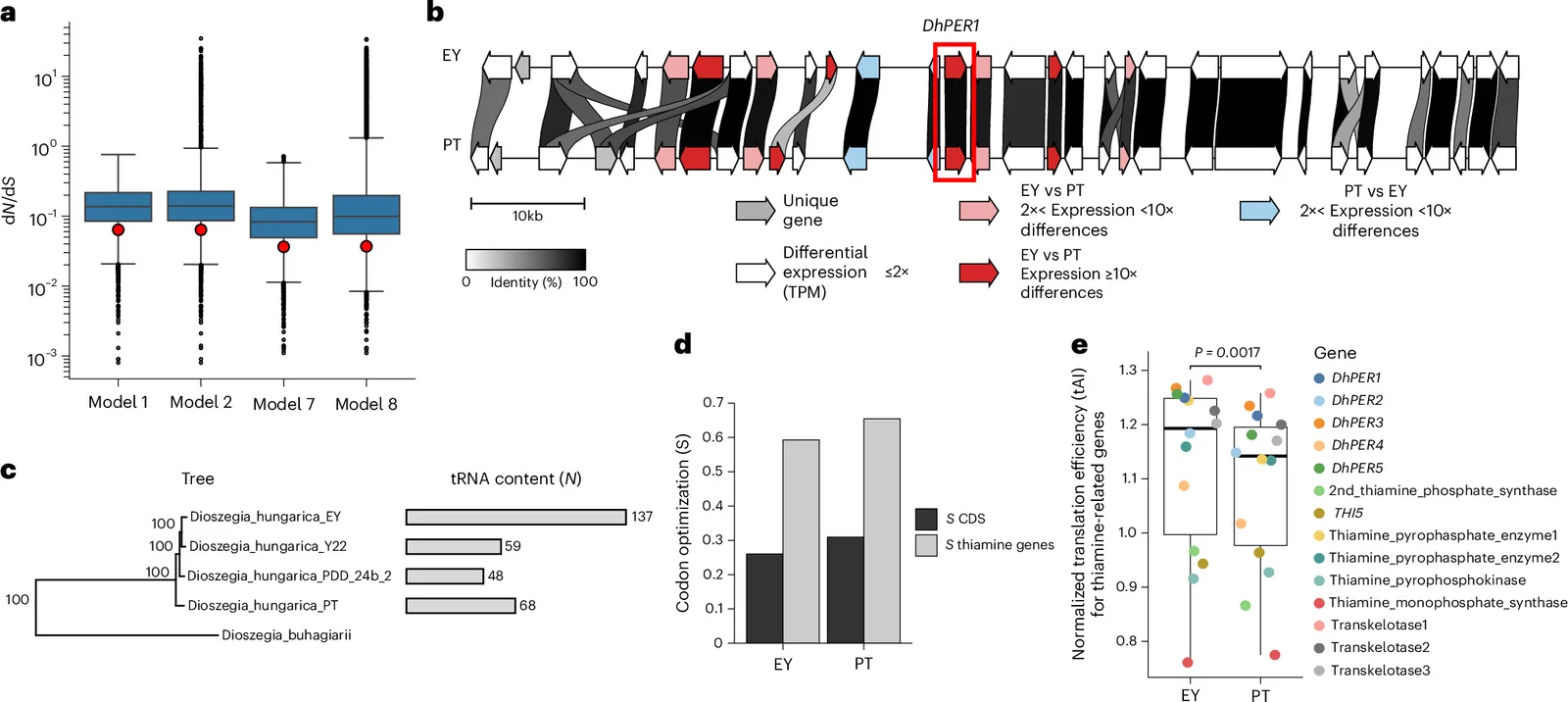
Evidence for selection of the *DhPER1*-driven cross-feeding mechanism. **a**, Evaluation of the conservation of *PER1* orthologues from all 7 *Dioszegia* genomes (*n* = 6,410 genes) carrying permease using phylogenetic analysis by maximum likelihood (PAML). The *x* axis represents different models of d*N*/d*S* analysis used by the programme. Models 1 and 7 are null models, and models 2 and 8 allow for positive selection. Boxplots indicate the median and 25th–75th percentiles. Whiskers show 1.5× IQR, with outliers shown as individual points. Red dots indicate the purifying selection (d*N*/d*S* close to 0) ratio of *DhPER1* under each model. **b**, Global alignment of the 100 kb *DhPER1* region using clinker^96^. Different-sized arrows represent the length of each gene with their direction of expression. Only paralogues with >35% amino acid identity (%) were annotated with linker. The colour of each gene indicates the transcriptional differences between each paralogue within this region, calculated on the basis of transcripts per million (TPM). **c**, tRNA content of each *D. hungarica* genome. The phylogeny is based on whole-genome alignment using the *Dioszegia buhagiarii* genome as outgroup. **d**, Comparison of codon optimization index *S* between thiamine-related genes and the whole-genome CDS in *Dioszegia* EY and PT. **e**, Comparison of the translation efficiency between EY and PT’s thiamine-related genes (*n* = 14) measured via the tRNA adaptation index (tAI) normalized to the mean tAI of the whole-genome CDS. Each dot is one of the 14 thiamine-related genes, coloured by gene; boxplots are defined as in **a**. *P* value was calculated using two-sided paired Wilcoxon signed-rank test. Source data

**Extended Data Fig. 6:**
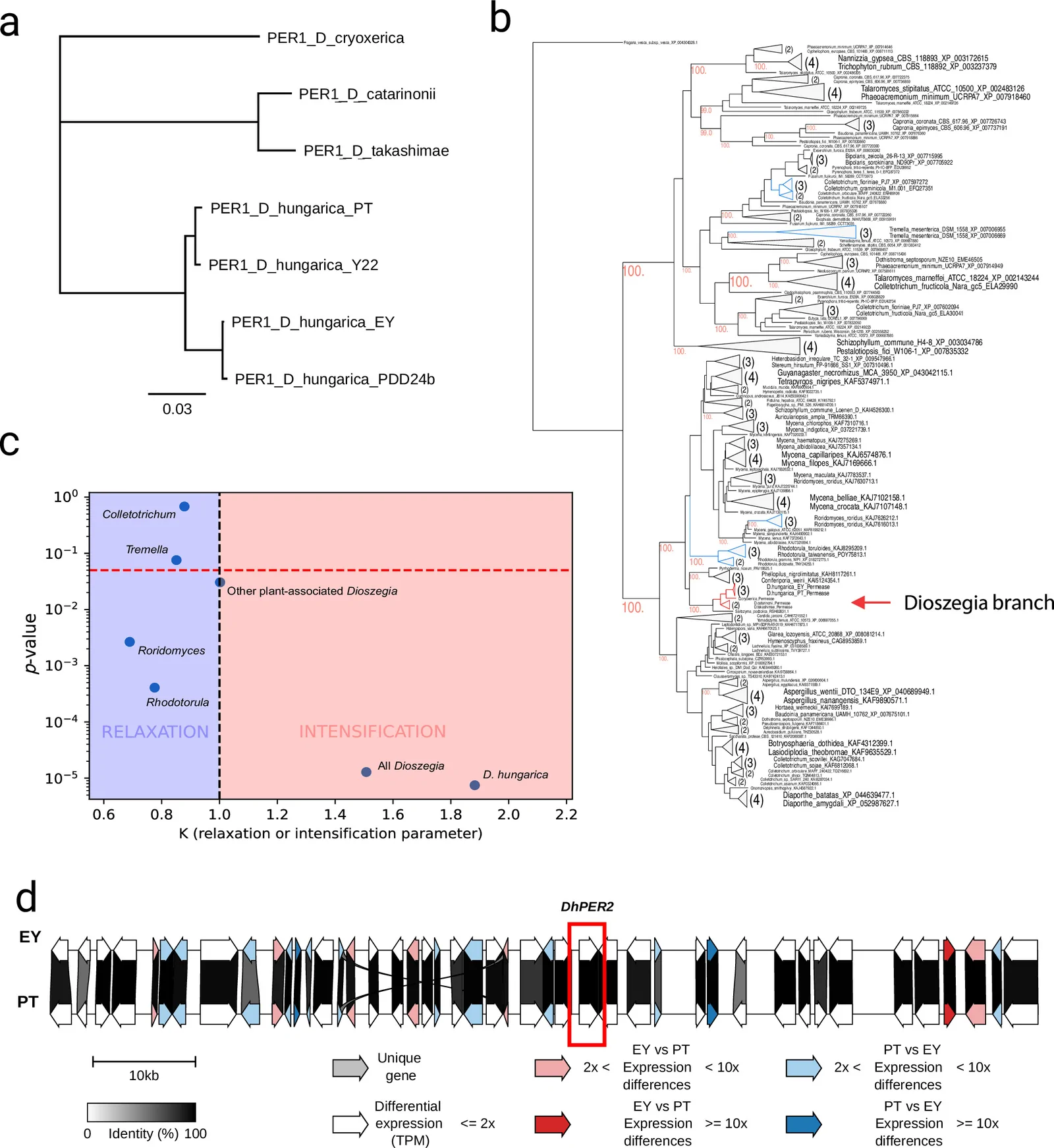
Evolutionary analysis of *Dioszegia PER1* genes. (**a**) Neighbor-joining tree of different *DhPER1* orthologous genes in different *Dioszegia* strains and species. The scale bar indicates 3% nucleotide sequence divergence. (**b**) Maximum likelihood phylogeny of *DhPER1* hits from protein BLAST against a non-redundant database. The branches highlighted in blue represents microbial lineages with permease genes undergoing relaxed selection. Red color branches represents three *Dioszegia* lineages corresponding to (**a**). Sequences with less than 10% similarity are collapsed within each branch. (**c**) RELAX analysis of selection intensity (K) across *DhPER1* homologs. Dots in the red section indicate *Dioszegia* lineages, where K > 1 represents intensified purifying selection, specifically prominent in the *D. hungarica* clade. Dots in the blue section represent *PER1* homologs in other fungal genera (for example, *Tremella*, *Roridomyces* and others) at similar taxonomic depths, which exhibit relaxed selection (K < 1). (**d**) Global alignment of the 100 kb *DhPER2* region using clinker^96^. Different sized arrows represents the length of each gene with their direction of expression. Only paralogs with more than 35% protein identity (%) were annotated with linker. The color of each gene indicates the transcriptional differences between each paralogue within this region calculated based on Transcripts Per Million (TPM).

How did this natural cross-feeding mechanism evolve and how did *DhPER1* differences between *Dioszegia* EY and PT arise? We addressed these questions by comparing the gene sequences of thiamine-related genes and investigating patterns of transcription and translation. We first examined evidence of selection on *DhPER1* by comparing the d*N*/d*S* ratio with all other gene families. We collected different *Dioszegia* strains from *A. thaliana* near Tübingen, and also included different type strains available from the fungal collection centre in our comparisons. In addition to the 4 available *Dioszegia* genomes^31,35,36^, we generated draft genome assemblies for 10 *Dioszegia* strains (Supplementary Table 3) and queried homologous genes to *DhPER1*. We identified *DhPER1* orthologues from 7 *Dioszegia* genomes comprising 4 species (Extended Data Fig. 6a). We aligned the predicted proteomes from all *Dioszegia* strains and mapped their coding sequences (CDS) to the protein alignment before modelling the d*N*/d*S* ratio for all genes. The results indicate that *DhPER1* is under strong purifying selection with a d*N*/d*S* ratio of ~0.05 (top 5% conserved across all genes) in all *Dioszegia* strains that carry it (Fig. 4a). We also investigated the diversification of *DhPER1* within the fungal kingdom. We identified putative orthologues for this gene in 171 fungal species using the NCBI non-redundant protein database. We then constructed a phylogenetic tree (Extended Data Fig. 6b) and assessed whether there was any evidence of selection during the diversification of this thiamine permease. Using the Relaxed Selection test^37^, we observed that this gene has been subject to variable levels of selection across its genealogy, including both relaxation and intensification, with the *Dioszegia* branch showing markedly intensified purifying selection on this gene (*K* = 1.50, Extended Data Fig. 6c). The evidence for intensified selection further increased when considering exclusively the *D. hungarica* branch (*K* = 1.88). In contrast, other fungal clades containing closely related species showed relaxed selection (for example, *Roridomyces* and *Tremella*, Extended Data Fig. 6c), indicating that this intensified selection is a unique signature of the *D. hungarica* lineage rather than a bias from evolutionary relatedness. The conservation and signature of strong selection on *DhPER1* indicate the importance of the thiamine transporter function for the survival of *Dioszegia* within the niche of *A. thaliana* leaves, potentially influenced by *Albugo*.

To further investigate the selection of *DhPER1* differential expression between *Dioszegia* EY and PT, we compared a 100 kb region around the *DhPER1* gene. We found several gene duplications, structural and copy number variations within this region, indicating active chromosomal rearrangements (Fig. 4b). Since chromosome rearrangements are known to be associated with altered gene expression^38^, we performed a long-read RNA-seq experiment from pure EY and PT cultures to assess the gene expression changes at this locus. *Dioszegia* EY showed overall increased gene expression in this region, as 8 genes showed increased expression more than 1-fold compared to PT, including 4 genes that increased more than 10-fold (Fig. 4b). Only two PT alleles showed increased expression in this region, both less than 3-fold. In contrast, another permease gene (*DhPER2*) resides in a less variable region, in which most genes did not show large variations in expression (Extended Data Fig. 6d).

**Extended Data Fig. 7:**
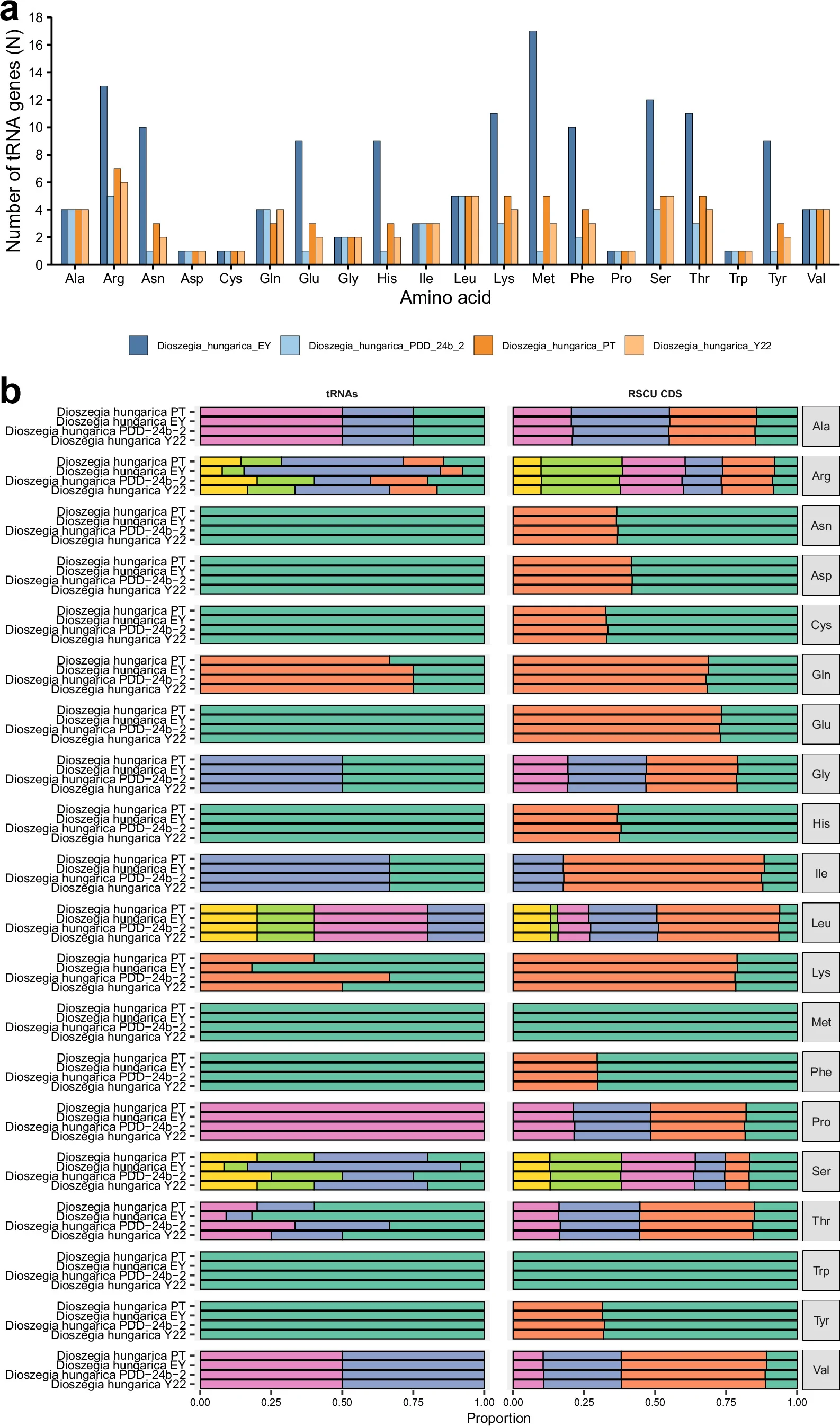
Comparison of tRNA content and relative synonymous codon usage (RSCU) among *D. hungarica* strains. (**a**) Absolute number of tRNAs for each amino acid from genomes of four *D. hungarica* strains. (**b**) Proportion of synonymous codons for each amino acid across the coding sequence of four *D. hungarica* strains. The proportion of tRNA genes decoding the same codon and amino acid are given. For each dataset (tRNA or RSCU) and strain, each codon is color-coded for the same amino acid. However, the colours are independent across amino acids.

In addition to changes in amino acid sequences and gene expression, natural selection may also act at the level of protein translation. To explore the impact of selection on translation in *Dioszegia* strains, we investigated codon usage across the genome. Specifically, we asked whether *Dioszegia* EY optimized its translational machinery correlated with the cross-feeding mechanism. We first screened the transfer RNA (tRNA) gene content of all 4 *D. hungarica* genomes, and found that the *Dioszegia* EY genome encodes a total of 137 tRNA genes, more than twice that of all other strains (Fig. 4c). The excess tRNA genes of *Dioszegia* EY span a total of 10 amino acids, with the methionine-encoding start codon showing the highest difference compared to the number of methionine-encoding tRNA in all other *D. hungarica* genomes (Extended Data Fig. 7a). We also compared the relative tRNA composition and codon usage bias which are largely similar among all 4 *D. hungarica* genomes (Extended Data Fig. 7b). Previous studies have shown that the copy number of tRNA genes in the genome correlates with tRNA abundance in the cytoplasm^39,40^ and that the initiation of translation is the limiting step^41^. With similar codon usage but a higher number of tRNA genes, our results indicate that *Dioszegia* EY can achieve higher protein translation rates than other strains, especially at the start of translation.

**Extended Data Fig. 8:**
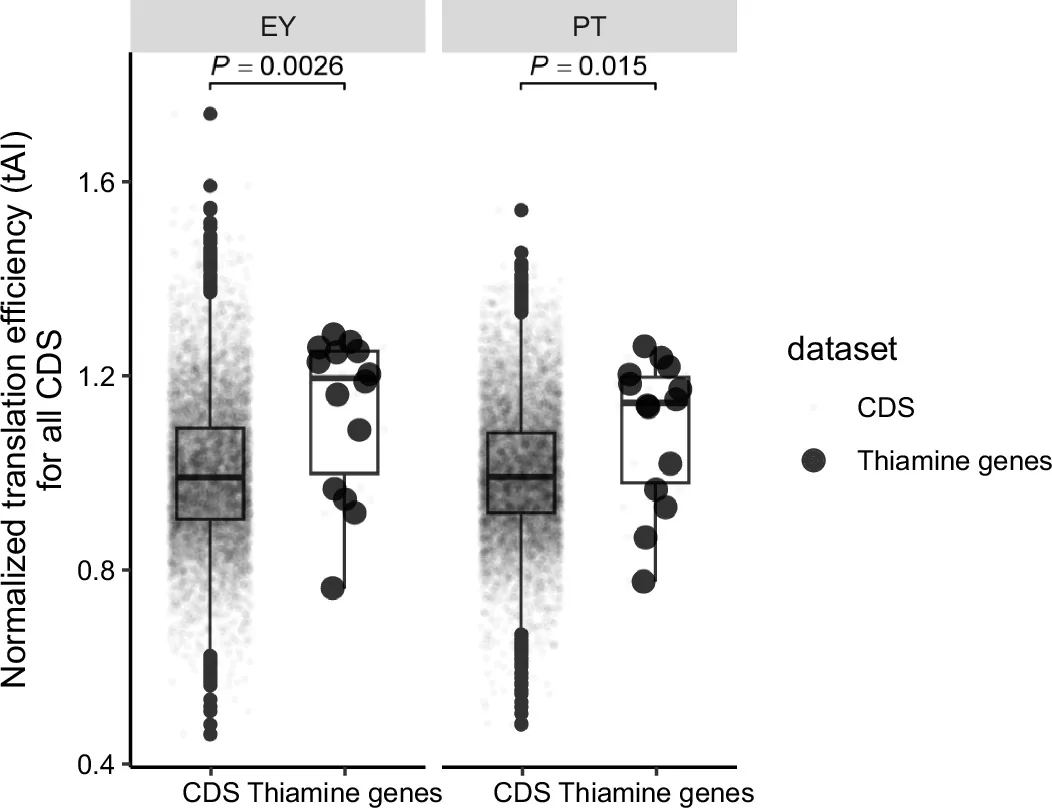
tRNA adaptation index (tAI) values between thiamine biosynthesis genes and the genomic CDS background in EY and PT. Box plots show normalized tAI (gene tAI ÷ mean CDS tAI of the same genome) for all coding sequences ≥300 bp (n = 7,510 for EY, n = 7,674 for PT) and the 14 thiamine-related genes. Box plots indicate the median and 25th–75th percentiles. Whiskers show 1.5x the interquartile range. *p* values were calculated using Wilcoxon signed-rank test. Source data

To determine whether these translational adaptations influence thiamine-related genes, we inferred the intensity of translational selection on the thiamine metabolism genes between *Dioszegia* EY and PT using the codon optimization index *S*. *S* is based on the tRNA gene pool, codon usage bias and the effective number of coding codons^42,43^. We found that the *S* indices of the complete sets of CDS were similar between EY and PT genomes, and the thiamine-related genes had more than twice as high *S* indices compared to all other CDS (Fig. 4d and Supplementary Table 4), suggesting substantial translational optimization in *D. hungarica* thiamine-related genes. We further validated the codon optimization analysis by assessing translation efficiency using a tRNA adaptation index (tAI), measuring the co-evolution between a given CDS and the respective genome tRNA pool^42^. In both EY and PT genomes, thiamine-related genes showed significantly higher translation efficiency (tAI) compared with the average for the rest of the genome (Extended Data Fig. 8). Notably, EY had significantly higher tAI values for these genes, including *DhPER1*, than PT (Fig. 4e and Supplementary Table 5, paired Wilcoxon *P* = 0.0017), indicating translational optimization correlated with the cross-feeding mechanism. Taken together, our results indicate that *Dioszegia* EY strain has experienced selection for an optimized translation of thiamine-related genes, especially *DhPER1*, suggesting that adaptation of *Dioszegia* EY to the leaf environment has involved regulation of the cross-feeding mechanism.

### *DhPER1*^EY^ facilitates the long-term survival of *Dioszegia* with *Albugo*

**Fig. 5:**
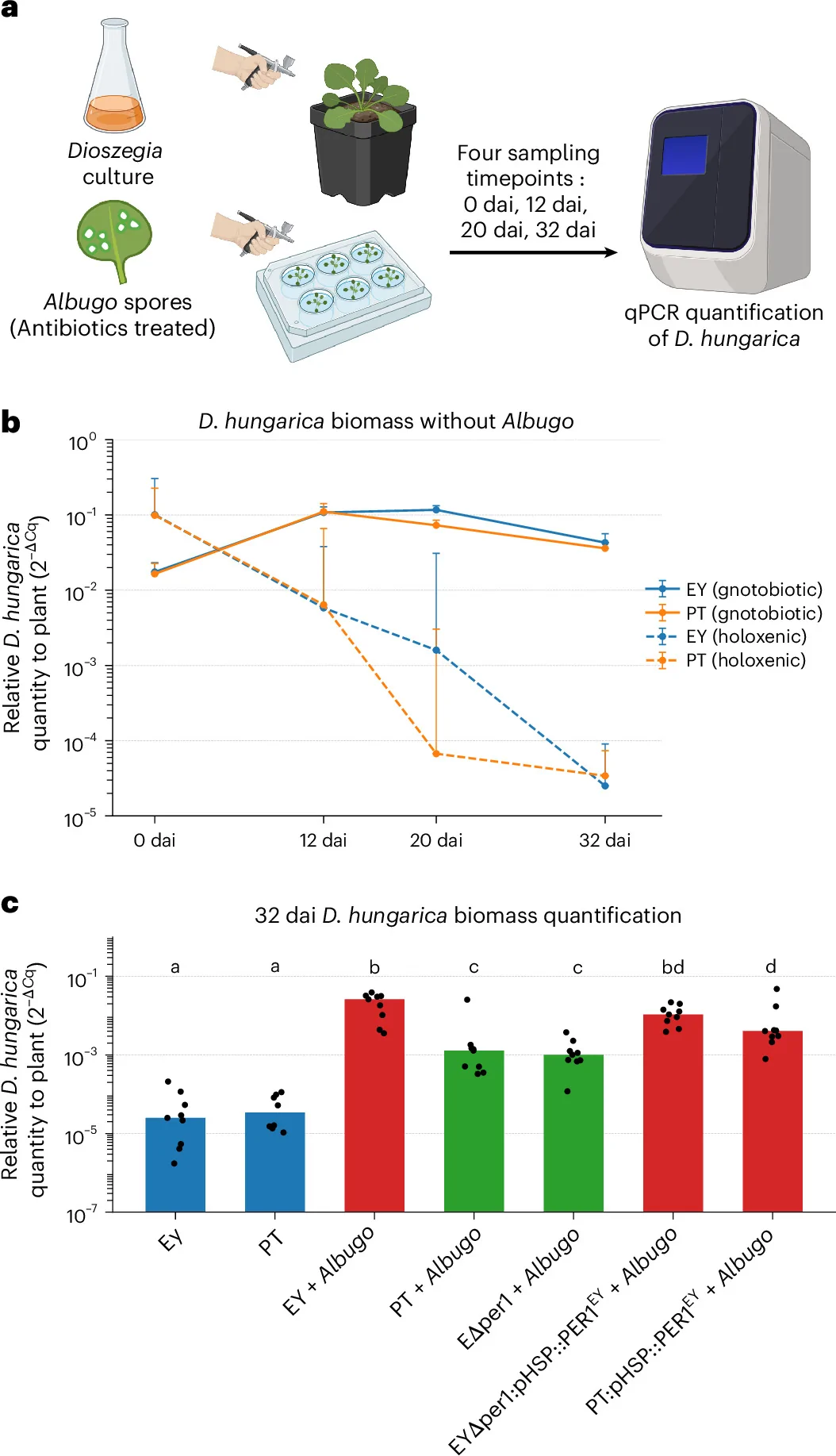
The ecological impact of the cross-feeding mechanism in a laboratory environment. **a**, Workflow of the long-term co-cultivation experiment. **b**, Biomass of *Dioszegia* EY and PT strains under gnotobiotic and holoxenic conditions during a 32-day time course without *Albugo*. qPCR analysis was used for biomass quantification. Each data point shows the median ± s.d. of *Dioszegia* biomass from *n* = 3 plants per time point for each gnotobiotic condition, *n* = 9 plants per time point for each holoxenic condition, and each plant sample contains 3 technical replicates. **c**, Biomass of different *Dioszegia* strains under holoxenic conditions at 32 dai. Bars show the median across 9 biological replicates (except PT with 8 biological replicates). Each dot represents a biological replicate, calculated as the median of 3 technical replicates. Different colours indicate different experiment groups (−*Albugo*, +*DhPER, −DhPER*). Group medians were compared using one-way ANOVA on Δ*C*_q_ (*F*_(6,55)_ = 48.5, *P* = 3.3 × 10^−20^), followed by two-sided pairwise Welch’s *t*-test on the 21 condition pairs with Benjamini–Hochberg FDR correction. Groups sharing the same letter are not significantly different (FDR < 0.05). Exact *P* values for all pairwise comparisons are provided in Source Data. In **b** and **c**, the *DhEF1a* gene was used for qPCR and normalized to the *EF1a* gene from *A. thaliana*. Panel **a** created in BioRender; Kremen, E. https://BioRender.com/rn8ybz6 (2026). Source data

Thiamine is an expensive but essential nutrient to synthesize, so what is the selective advantage to *Dioszegia* of this mechanism? Many in vitro studies have shown that microbes produce ‘public goods’—metabolic products that are costly to produce but benefit individuals in shared environments independently of species^9,44,45^. In addition, synthetic ecology experiments suggest that this lifestyle promotes long-term stability and stress resilience of the public-good producer in a community, especially under transient environmental conditions^9^. To examine this idea, we designed a long-term experiment, in which *Albugo* was co-cultivated with *Dioszegia* EY, PT, EYΔper1 mutant or complementation strains EYΔper1:pHSP::PER1^EY^ and PT:pHSP::PER1^EY^, to track the *Dioszegia* population over most of *A. thaliana*’s vegetative growth stage (Fig. 5a). First, to investigate whether *Dioszegia* EY and PT can successfully colonize *A. thaliana* leaves, we assessed their growth in both gnotobiotic and holoxenic (plants grown on non-sterile soil) conditions without *Albugo* at different time points (12, 20 and 32 dai). Interestingly, both *Dioszegia* EY and PT colonized well under gnotobiotic conditions, whereas their populations gradually became extinct in the presence of an endogenous microbiome under holoxenic condition (Fig. 5b). This result indicates the difficulty for *D. hungarica* to persist alone in an *Albugo-*free community.

**Extended Data Fig. 9:**
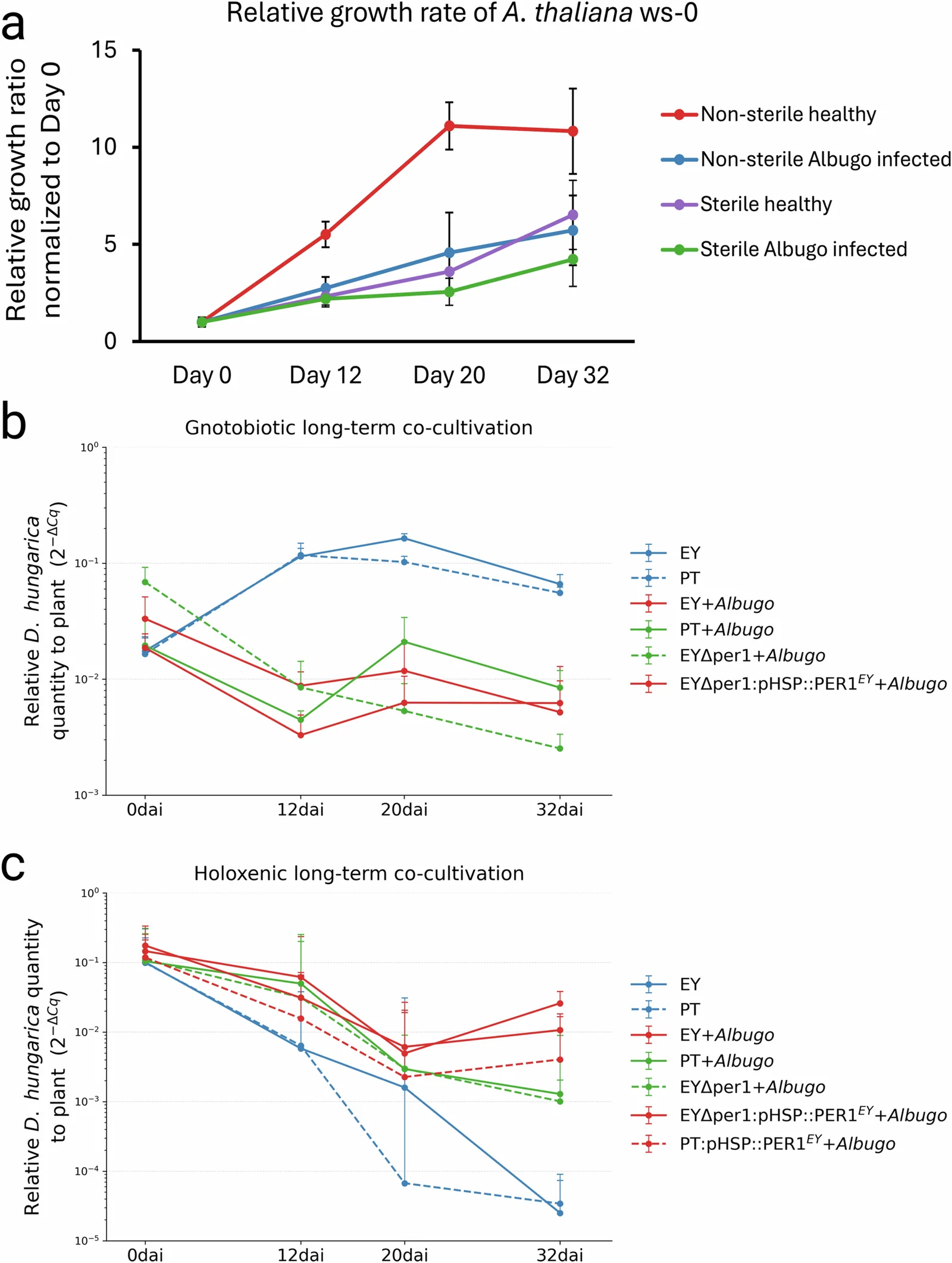
Supplementary data for the long-term co-cultivation experiment. (**a**) Relative growth ratio calculated based on the fresh weight of *A. thaliana* ws-0 ecotype under sterile and non-sterile conditions. (**b**) The relative *Dioszegia* quantity measured by qPCR under gnotobiotic condition in different treatment groups during the 32 day period. In all panels, each time point shows the median with positive standard deviation of 3 samples, and each sample contains three technical replicates. (**c**) The relative *Dioszegia* quantity measured by qPCR under holoxenic condition in different treatment groups during the 32 day period. In all panels (lines), each time point shows the median with positive standard deviation of n = 9 plants per treatment group, except EY+*Albugo* at day 20 (n = 8), and each sample contains three technical replicates. In both (**b**) and (**c**) the *DhEF1a* gene was used for qPCR and normalized to the *EF1a* gene from *A. thaliana*. Source data

In the presence of *Albugo*, the population stabilized over the growing period under both conditions: not only did the biomass change more slowly, but the standard deviation in all treatment groups was also lower in the later compared with earlier sampling dates (Extended Data Fig. 9b,c). On the basis of the results from both conditions, we believe that the 32 dai timepoint in the growth chamber best reflects the long-term stability of different *Dioszegia* strains. Interestingly under holoxenic conditions, *Albugo* infection ‘rescued’ *Dioszegia* from extinction and increased its population by more than 100-fold (Fig. 5c and Extended Data Fig. 9c). The populations of *Dioszegia* strains expressing *DhPER1* (EY, EYΔper1:pHSP::PER1^EY^, PT:pHSP::PER1^EY^) were significantly larger compared with *DhPER1* non-expressing strains (EYΔper1, PT) (one-way ANOVA *P* = 3.3 × 10^−20^). In contrast, we did not observe the same beneficial effects on *Dioszegia* under gnotobiotic conditions where the microbial interaction is primarily pairwise between *Dioszegia* and *Albugo* (Extended Data Fig. 9b). In this condition, *Albugo* quickly overtook and restricted the growth of all *Dioszegia* strains throughout the colonization period. These results align with results from a previous in vitro microbial ecology study^9^ and indicate that thiamine cross-feeding is crucial for the long-term stability of *Dioszegia* with *Albugo*. However, this benefit is evident only within complex microbial communities where strong competition is present among community members.

## Discussion

**Extended Data Fig. 10:**
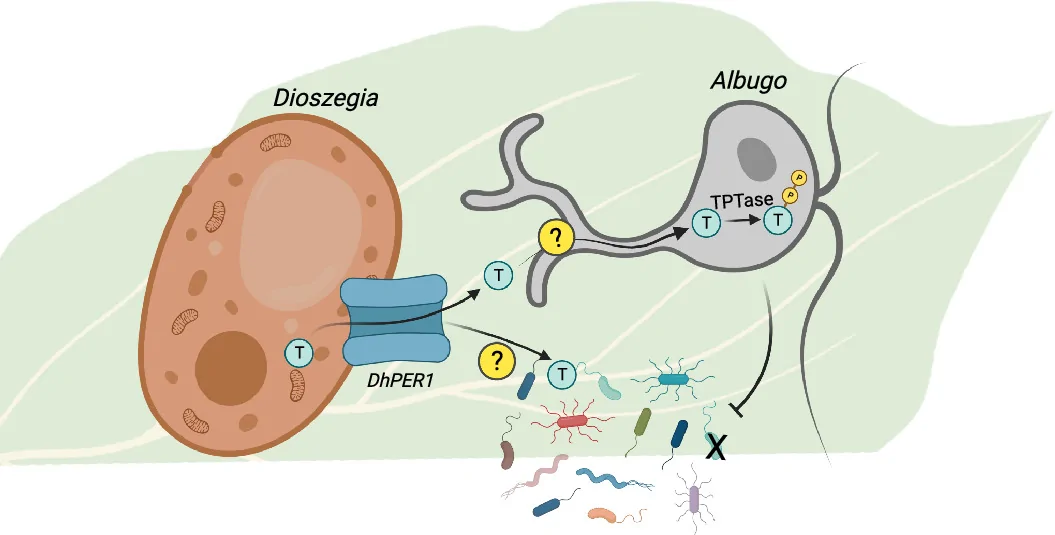
Working model of *Dioszegia*–*Albugo* interactions in an ecological context. *Dioszegia* exports thiamine via the permease DhPER1, which is taken up by *Albugo* through an uncharacterized membrane transporter (question mark in *Albugo* cell). The only thiamine-related gene in the *Albugo* genome encodes a thiamine pyrophosphokinase (TPTase) that irreversibly converts free thiamine into thiamine pyrophosphate — a bioactive form that cannot exit the cell, effectively sequestering the thiamine acquired from *Dioszegia*. A second question mark indicates the uncertain extent to which secreted thiamine may be utilized by other phyllosphere microbes. The inhibitory arrow from *Albugo* to the microbiome reflects previous findings that *Albugo* selectively suppresses certain phyllosphere microbes^18^. This figure is generated using BioRender.

Our study identified two prevalent hub microbial taxa from natural *A. thaliana* leaf microbiome that interact and have adapted to each other in nature via vitamin cross-feeding (Extended Data Fig. 10). The basidiomycete yeast *Dioszegia* invests in surplus thiamine production and secretion to facilitate the colonization of the other hub taxon *Albugo*, whereas *Albugo* establishes an environment conducive to the long-term survival of *Dioszegia*. The persistent presence of *Albugo* across the life cycle of *A. thaliana* further stabilizes the microbiome over time^15^. Combined with previous work showing that *A. laibachii* creates niches by suppressing plant immunity and inhibiting the proliferation of certain microbes within the microbiome^18,46^, we propose that the *Albugo*-centred interaction network is a fundamental component of *A. thaliana* leaf microbiome assembly and stability. Our work complements recent studies focusing on within-microbiota competition^47,48^, providing an integrated view of molecular mechanisms behind the regulation of microbiome assembly by key microbes. In the context of the host-associated microbiome, a majority of microbial cooperations are associated with nutrient exchange, such as the arginine exchange in the human gut^49^, and the carbon exchange between bacteria and arbuscular mycorrhizal fungi^50^. These findings underscore the importance of such mutualistic interactions in mediating microbiome stability and support the hypothesis that nutrient-driven co-dependencies are key drivers of microbiome assembly and sustainability^6^.

Signatures of selection in *DhPER1* and other thiamine-related genes suggest adaptive specialization in basidiomycete yeasts supplying public goods in *A. thaliana* leaves. The observed adaptation in *Dioszegia*, together with the loss of key biosynthetic functions in *Albugo*, aligns with the ‘Black Queen Hypothesis’, in which leaky public goods drive stable metabolic dependencies^51^. Our results also indicate that this mutualistic interaction is context dependent and characterized by a trade-off between competition and facilitation. Under gnotobiotic conditions, *Albugo* restricts the free growth of *Dioszegia* (Extended Data Fig. 9b), probably reflecting niche competition for limited host resources in a simplified system. However, in a natural ecological context (holoxenic conditions), the relationship shifts towards facilitation. While *Dioszegia* alone faces potential competitive exclusion by the native microbiota (Fig. 5b), the presence of *Albugo* enables its long-term persistence (Fig. 5c). This ‘ecological rescue’ suggests that the benefit provided by *Albugo* to *Dioszegia* is primarily indirect, where *Albugo* acts as a niche engineer that modifies the leaf environment—potentially through immune suppression^46^, microbial antagonism^18^ or modulation of host metabolites exuded during infection^52^, sheltering its metabolic partner. The provision of thiamine by *Dioszegia* might fuel the metabolic demands of *Albugo* during these energy-intensive processes of host colonization and immune manipulation. This asymmetric mutualism, combining direct metabolic supply with indirect niche stabilization, indicates that the interaction network extends beyond a single gene-loss dependency and probably involves multiple microbial partners and ecological functions. This interaction complexity opens an intriguing area of fundamental research, highlighting the need to combine computational and experimental approaches to fully understand the direct and indirect metabolic interactions within the microbiome.

Vitamin auxotrophy is a crucial feature in bacterial communities across various environments, including plants, oceans and the human gut, and is considered a driving force in community stability^53–55^. However, in plant-associated obligate microbes, such as downy mildews^56^ (*Hyaloperonospora* spp.), rusts^57^ (*Puccinia* spp.) and arbuscular mycorrhizal fungi^58^, nutrient auxotrophy is primarily regarded as a host-dependent trait, despite their central roles in both leaf and root microbiomes. Previous studies observed that auxotrophic microbes can only store nutrients sufficient for a few population doublings^53^. Given that the successful colonization of *Albugo* and other obligate microbes can take weeks to months^34,59^, these microbes require a steady nutrient supply beyond what is available for just a few population doublings to successfully infect their hosts. Combined with our findings on the *Albugo*–*Dioszegia* mutualism, we speculate that the colonization of obligate biotrophic microbes might be functionally dependent on auxotrophic nutrients from the microbiome, not only from the host. Further exploration of this concept could lead to novel strategies to manage obligate plant pathogens or symbionts by harnessing microbiome-originated nutrient supplies, thereby enhancing disease management and promoting crop production.

## Methods

### Collecting and processing of field *A. thaliana* leaf samples

We shared most parts of the sampling trip with those of ref. ^10^. We took samples from the same 12 sites across Sweden, Spain, France, and replaced three sites from Northern Germany to three sites near Tübingen, Germany (Supplementary Table 6). We harvested *A. thaliana* from February to May at the same developmental stage (bolting/flowering stage) for one (one site), two (one site) and three (13 sites) consecutive years. Using sterilized tweezers, whole leaf samples were prepared by roughly cleaning the leaves of dirt, inserting them into 2 ml tubes and storing the tubes immediately in dry ice. The endophytic samples were instead prepared by subjecting leaves to surface sterilization. The rosette was surface sterilized by washing with 80% ethanol for 15 s, followed by 2% bleach (sodium hypochlorite) for 30 s. Rosettes were rinsed three times with sterile autoclaved water for 10 s before being placed in a screw-cap tube and frozen on dry ice as endophytic samples. All samples were shipped to the Max Planck Institute for Plant Breeding Research in Cologne (Germany) and stored at −80 °C until further processing. We took 6–10 plants from each site, with half processed as whole leaves and the other half as endophytes. In total, 347 samples were collected.

### DNA extraction from field samples and amplicon library preparation

DNA extraction and library preparation of all field samples were carried out with the same previously described method^17^. Briefly, 0.2 g acid-washed and sterilized zirconium beads at different sizes (0.1–0.5 mm diameter) and 500 μl DNA extraction buffer were added to plant samples. The buffer consisted of 0.5% SDS, 50 mM Tris buffer at pH 8, 200 mM NaCl, 2 mM EDTA, 1 mg ml^−1^ lysozyme and 100 mg ml^−1^ proteinase K. The samples/beads were first incubated for 45 min at 37 °C, and were then beaten using a Bertin Precellys 24 (Bertin Technologies) at 6,300 r.p.m. for 2 × 45 s with a 15 s pause. A second incubation was then carried out at 37 °C for 45 min in the presence of 10 µg ml^−1^ RNAse. The tubes were then centrifuged at 10,000 r.p.m. for 2 min and the liquid was transferred to a new tube. The nucleic acids were cleaned up with 600 μl phenol/chloroform/isoamyl alcohol (25:24:1) and chloroform/isoamyl alcohol (24:1), then precipitated by adding 0.1× of the sample volume of 3 M sodium acetate and 1,500 μl 100% ethanol and centrifuging at 4 °C at 15,000 r.p.m. for 40 min. The product was washed twice with 70% ethanol and eluted with 30 μl 10 mM Tris buffer pH 8.0. To avoid potential problems with PCR inhibition, samples were mixed 1:1 with 20% Chelex-100 and allowed a 30-min contact time, after which the liquid fraction was recovered and stored at −20 °C until further use.

PCR amplification was performed in two steps to allow the use of blocking primers to decrease plant plastid contamination that occurs with 16S amplification. The samples were diluted with 10 mM Tris HCl to 50 ng μl^−1^ before being used in the first PCR step. In the first step, universal primers and blocking oligos (Supplementary Table 7) were used together to amplify targeted regions. For each target taxonomic group, two universal primer pairs were used. Each of the 20 μl reactions contained 0.2 μl Q5 high-fidelity DNA polymerase (New England Biolabs (NEB)), 4 μl Q5 High-GC buffer, 4 μl Q5 5× reaction buffer, 0.16 μl of each forward and reverse primer (10 μM), 0.5 μl of each blocking primer, 0.5 μl dNTPs (10 μM), 1 μl template DNA and nuclease-free water to a final volume of 20 μl. Triplicates were run in parallel on 3 independent thermocyclers (Bio-Rad); cycling conditions were 95 °C for 40 s, 10 cycles of 95 °C for 35 s, 55 °C for 45 s, 72 °C for 15 s, and a final elongation at 72 °C for 3 min. The 3 reactions were combined and 10 μl were used for enzymatic cleanup with Antarctic phosphatase and Exonuclease I (NEB; 0.5 μl of each enzyme with 1.22 μl Antarctic phosphatase buffer at 37 °C for 30 min followed by 80 °C for 15 min). The second step of PCR was carried out in a single 50 μl reaction. The reaction included: 0.5 μl Q5 DNA polymerase (NEB), 10 μl Q5 High-GC buffer, 10 μl Q5 5× buffer, 1 μl dNTPs (10 μM), 0.83 μl of each barcoded primer (10 μM) and 26.34 μl nuclease-free water. A volume of 0.5 μl of the cleaned-up first-step PCR product was added to each reaction. The protocol used in the thermocycler was: 95 °C for 40 s, followed by 25 cycles of 95 °C for 35 s, 55 °C for 45 s, 72 °C for 15 s, and 72 °C for 2 min. The barcoded amplicons were then cleaned using AmPure XP beads (Beckman-Coulter) following manufacturer protocol. Magnetic beads solution (40 μl) was added to the 50 μl barcoding reaction, shaken for 5 min at 700 r.p.m. at room temperature, then washed twice with 200 μl 80% ethanol, dried and resuspended in 25 μl 10 mM Tris. Amplicons were quantified in duplicates with the PicoGreen system (Thermo Fisher) and samples were combined in equimolar amounts into one library to reach 8 pmol per library. The final libraries were also cleaned with 0.8× volume AmPure XP purification beads and eluted into 40 μl. Libraries were prepared with the MiSeq Reagent Kit v.3 for 2 × 300 bp paired-end reads (Illumina) with 3% PhiX control. Quality assessment and the eventual presence of contaminants were assessed using a bioanalyzer (Agilent). The final concentration of the pooled libraries was finally assessed using a Quantus Fluorometer (Promega) following the indications of the manufacturer. Since there is a maximum of 50 barcoded primers per locus available, we performed 8 runs in total to cover all the samples. The sequencing libraries were run by the Illumina MiSeq platform in house.

### Amplicon data processing and OTU table filtering

The detailed steps for raw amplicon data processing are summarized in Supplementary Information, with all scripts available at Amplicon_data_analysis/Amplicon_sequencing_processing^60^. The raw OTU tables for each taxonomic group were exported from QIIME (v.2023-07)^61^ and were handled with Python. We first chose the amplicon with the highest number of classified OTUs from each taxon group for further filtering. We next applied several modification steps sequentially: (1) filtering of OTUs that do not belong to the corresponding taxonomic group (for example, non-bacterial OTUs within the BacV5 amplicon); (2) merging all OTU tables from each taxonomic group; (3) filtering of OTUs with fewer than 50 counts and present in only one sample; (4) separating OTU tables by compartment: whole leaf and endophytes; (5) selection of the amplicon with higher number of classified taxa to proceed further for each taxonomic group; (6) normalizing the count of each OTU to the total count of its corresponding taxonomic group of each sample, and calculating the relative abundance; and (7) selection of the samples containing OTUs from all four taxonomic groups. The detailed Python script for this step is available under Amplicon_data_analysis/OTU_table_processing/filter_otu_and_tax_final.ipynb^60^. The final OTU tables were deposited in Amplicon_data_analysis/OTU_table_processing/processed_OTU_tables.

### Diversity analysis

On the basis of the final OTU table, we calculated the Shannon index using the R package vegan^62^ and grouped the OTUs by country of origin for comparison. To control for potential bias introduced by varying sample sizes across different countries, we subsampled the reads from each sample 100 times and conducted Kruskal–Wallis tests for each iteration. We only observed 10 of the 100-times subsampled reads, showing significant differences in the Shannon index across the four countries, supporting the idea that the results without subsampling are unbiased.

For analysing beta diversity, distance matrices using Bray–Curtis distance were calculated for all samples and the subsets of whole-leaf and endophytic samples using the R packages vegan and ade4 (ref. ^63^). Plotting of the results and statistical testing using permutational multivariate analysis of variance (PERMANOVA) were conducted with the first and second principal components using ggplot2. For PERMANOVA analysis, all groups were compared at once for both endophyte and whole-leaf plots using the PERMANOVA function in the vegan R package.

To identify the most occurring taxa from each country, we calculated the relative abundance of the 10 most abundant classes for bacteria, fungi and other eukaryotes. For oomycetes, we calculated the 10 most abundant genera since all samples only contain one (endophytes) or two (whole leaf) oomycete classes. We used the phyloseq package^64^ for this analysis. Samples were first merged by country and subsequently normalized to relative abundance, and OTUs with an undetermined class were grouped in the category ‘unassigned’. All classes with lower abundances than those categorized in the top 10 were grouped into the category ‘other.’ Subsequently, the average relative abundances were plotted using ggplot2 (ref. ^65^).

All R scripts for analysis and figure plotting are summarized in Amplicon_data_analysis/diversity_analysis.

### Network analysis

Before network construction, the OTU table was filtered to only contain OTUs present in 10 or more samples to retain the most conserved taxa among all samples. The subsequent network construction was performed using Fastspar^24^, using the recommended settings for bootstrapping and permutation counts. The resulting *P* value and median correlation tables were transformed into an edge table using R, keeping all interactions with a *P* value lower than 0.1 and a none-zero value. We selected this relatively loose thresholds since we have applied strict filtering for the most conserved OTUs, so we wanted to retain most of their interactions including the weak interactions. We used the built-in function of the network analyser Cytoscape (v.3.9.1)^66^ to calculate degree, betweenness centrality and closeness centrality, and the final visualizations of the networks were constructed utilizing Gephi (v.0.10.1)^67^. The script for constructing the network is available at Amplicon_data_analysis/Network_analysis_and_Albugo-Dioszegia_interaction^60^.

### Microbial growth conditions

All microbial strains used in this study are summarized in Supplementary Table 2. All fungi used were grown on potato dextrose agar (Carl Roth) and bacteria in nutrient broth agar (Carl Roth). The spore collection and inoculation of the *Albugo laibachii* was performed according to ref. ^19^. Briefly, infected leaves with clear white pustules were collected and resuspended in Milli-Q water in 50 ml tubes. The tubes were incubated on ice for 1 h, and the spore suspension was filtered with a 40 μm filter (Greiner Bio-One). The spore suspension was then sprayed on plants using a spray gun with an air pump, and plants were incubated at 8 °C in the dark overnight. Infected plants were kept under 8-h light and 16-h dark cycles, a 20 °C day and 16 °C night temperature, and 60% humidity for 14 days or until infection symptoms appeared.

### Gnotobiotic microbial inoculation on *A. thaliana*

The gnotobiotic microbial inoculation system was adapted from refs. ^68,69^ with some modifications. Briefly, *A. thaliana* ws-0 (Wassilewskija) seeds were sterilized for 6–12 h with chlorine gas. Sterilized seeds were immediately sown on 0.5× Murashige and Skoog (MS) with 0.75% agar and incubated under 8/16-h light/dark cycles with a 20 °C day and 16 °C night temperature, and 60% humidity. After 7 days, the germinated seedlings were placed onto 12-well plates (Greiner Bio-One) with each well filled with 2.5 ml 0.5× MS agar. Plates with seedlings were further incubated for 3 weeks before spraying.

We primarily tested fungi (yeasts) and few bacteria in this study, either because isolation of the predicted *Albugo*-interacting bacteria was unsuccessful or these are slow growers (actinobacteria) and therefore not compatible with co-inoculation with *Albugo*. To spray different bacteria and fungal strains, we directly streaked fresh bacteria or yeast colonies (<5-day growth) from agar plates and resuspended them in 10 mM MgCl_2_ solution. We then diluted the strain resuspensions to reach optical density at 600 nm (OD_600_) of 0.5. To spray *Fusarium* and *Stagonosporopsis* strains, 10 ml 10 mM MgCl_2_ was added into each plate that was fully covered with fungal mycelium and spores (>14-day growth). We then used a 1 ml pipette tip to streak the plate and release a maximum number of spores, which were filtered with a 40 μm filter (Greiner Bio-One). We then counted the spores with a haemocytometer and diluted the spores to reach 10^4^–10^5^ spores per ml. After dilution, all microbial solutions were transferred into airbrush guns (Conrad Electronics) and sprayed two times (~40 μl each) with the air pump (Conrad Electronics) at 0.5 bar pressure. After spraying buffer and different microbial strains, we waited 2–3 h to allow the evaporation of excess water before spraying *A. laibachii*. To minimize the effect of *A. laibachii* spore-associated microbes and ensure a gnotobiotic infection environment, we treated the *A. laibachii* spores with antibiotics. We first incubated the *Albugo-*infected leaves with autoclaved Milli-Q water on ice for 1 h to facilitate the release and hatching of *A. laibachii* sporangia (one full 50 ml tube of infected leaves filling up with water). The spore solution was then filtered with a 40 μm filter (Greiner Bio-One) and centrifuged at 4,000 *g* in 15 ml tubes for 10 min at room temperature. We next removed the supernatant and resuspended the spore pellet gently with a 5 ml antibiotic solution containing 2.5 mg kanamycin, streptomycin and rifampicin, plus 1.25 mg geneticin. We incubated the spores with the antibiotic solution at room temperature for 25 min in the dark, before pelleting again by centrifuging the tube at 4,000 *g* for 10 min. The supernatant was removed and the antibiotic-treated spores were washed twice with autoclaved Milli-Q water and once with 10 mM MgCl_2_ using the same centrifugation conditions. The antibiotic-treated spores were resuspended with 10 mM MgCl_2_ and adjusted to 10^6^–10^7^ spores per ml before spraying on the sterile plants using the same airbrush setup as for other microbes. After spraying the *A. laibachii*, all 12-well plates were sealed with a breathable tape and bagged with transparent bags to keep the humidity at >90%. The bagged plates were incubated at 8 °C in the dark overnight before transferring to the growth chambers. The transparent bag was removed after another day (day 2). The infected plants were scored and harvested for qPCR at 12 dai.

### Quantification of *Albugo* in planta with scoring and qPCR

To score infected plants from gnotobiotic infection systems, we first removed all root tissue of each plant and recorded the number of infected leaves for each plant. We then divided the total number of infected leaves by the total number of plants from each treatment group to calculate the average number of infected leaves per plant (ANIL). Since the efficacy of *A. laibachii* infection varies considerably among individual plants, we tried to ensure the robustness of the experiment by only including results from experiments with 0.5–2 ANIL in the control group (buffer and *A. laibachii*). The *A. laibachii* infection was considered unsuccessful if the ANIL was below 0.5. In addition, the difference between buffer and treatment groups cannot be observed if the infection is too good (ANIL > 2.5), since there were only 4–5 mature leaves in total when *A. thaliana* plants were sprayed. To control for the impact of zeros in statistical analysis and balance results from experiments with varied infection levels, we also added a pseudo-count (0.05) to all scoring data before calculating the ANIL.

After scoring, the rosette was put into 50 ml falcon tubes and divided into 3–6 groups as biological replicates with 3–5 plants per group for DNA extraction and qPCR. The rosette tissues were snap frozen in liquid nitrogen and ground with mortars and pestles. We used the silica column-based DNeasy plant mini kit (Qiagen) following manufacturer protocol. DNA concentration was measured with a NanoDrop spectrophotometer (Thermo Fisher) and adjusted to 5 ng μl^−1^ with Milli-Q water before processing for the qPCR experiment. We used SsoAdvanced Universal SYBR Green Supermix and a CFX Connect real-time PCR detection system (Bio-Rad) for qPCR reaction following manufacturer instructions. We used 20 μl reactions and 5 μl (25 ng) DNA as template. For each sample, we performed two reactions for the *EF1α* genes of both *A. laibachii* and *A. thaliana*. Quantification cycle (*C*_q_) values obtained from *A. laibachii* DNA amplification were first normalized to *A. thaliana* DNA amplicon from the same sample using the formula ${2}^{-\Delta {C}_{q}}$ to calculate the relative *A. laibachii* quantity per sample. Then the relative *Albugo* quantities of each treatment sample were normalized to the average relative *Albugo* quantity of the buffer control to compare among different experiments. Each data point in the figure represents 3 technical replicates.

### ELISA assay

To measure the secretion of thiamine, *Dioszegia* strains (EY, PT, EYΔper1 and the complemented strain) were cultured in thiamine-free Yeast Nitrogen Base (YNB) medium (FORMEDIUM) supplemented with 1% glucose and a complete set of amino acids (FORMEDIUM). After 3 days, the cultures were collected and filtered through a 0.2 μm filter to separate the extracellular fraction from the yeast cells. The resulting cell-free supernatants were analysed immediately using a competitive Vitamin B1 (Thiamine) ELISA kit (Abbexa, abx150384) following manufacturer instructions.

Briefly, 50 μl of the filtered samples or thiamine standards (ranging from 3.7 ng ml^−1^ to 300 ng ml^−1^) were added to a 96-well microplate pre-coated with a thiamine-specific antibody. Biotin-conjugated Detection Reagent A (50 μl) was added to each well, and the plate was incubated for 1 h at 37 °C. Following incubation, the plate was washed three times with 1× Wash Buffer to remove unbound components. Subsequently, 100 μl of HRP-conjugated Detection Reagent B was added to each well and incubated for 30 min at 37 °C. After a second washing step (5 times), 90 μl of TMB substrate was added and incubated at 37 °C for 5 min in the dark. The reaction was terminated by adding 50 μl of Stop Solution.

Optical density was measured at 450 nm using a microplate reader. Thiamine concentrations were calculated by plotting the OD values against a standard curve. The raw data and the standard curve were deposited in the Gitlab repository at /Scripts_for_data_analysis_and_figure_plotitng/scripts_for_figure_ploting/ELISA_data.xlsx^60^.

### *Dioszegia* genome information

The detailed DNA extraction, sequencing, assembly and annotation steps are summarized in Supplementary Information. All sequenced strains and the quality assessment of assemblies are summarized in Supplementary Table 3. All raw data were submitted to NCBI Sequence Read Archive (SRA) under the BioProject PRJNA1190083. All genome assemblies were submitted to GenBank and the accession number for each genome is recorded in Supplementary Table 3. Copies of the assemblies, gene predictions and functional annotation used in this project were posted at /Supplementary_files/Dioszegia_genomes_and_annotations/. Detailed scripts used for all above steps are available at /Scripts_for_data_analysis_and_figure_plotting/Genome_analysis.

### Extraction of thiamine metabolism genes and cloning of *DhPER1* orthologues in different *Dioszegia* strains

We manually inspected the functional annotation result of *Dioszegia* EY and PT genomes and extracted the thiamine metabolism genes by searching the keyword ‘thiamine’ in the functional annotation file. In total, 14 genes were identified from each genome. We paired these genes through alignment and validated them by Sanger sequencing in case the paired genes were predicted to be substantially different in gene size (>10%) or exon borders. After Sanger sequencing validation, all alleles ended up with a similar size and were translated to amino acid (AA) sequence for alignment. The CDS and AA sequence were then aligned to calculate the SNPs per kilobasepair and variations per 100 amino acids.

To search for genes homologous to *DhPER1* from different *Dioszegia* strains, we used two strategies. First, we searched different parts of *DhPER1* in all available *Dioszegia* genome assemblies from both online databases and in-house sequencing using Geneious Prime v.2024.0.5 (https://www.geneious.com). We found six *DhPER1* homologous genes using this method. However, we did not find the *DhPER1* paralogue from the nanopore assembly of *Dioszegia* Y22 strain. Therefore, we performed a PCR test using the *DhPER1* primers, and the PCR test resulted in the identification of the Y22 paralogue of *DhPER1*. This result also indicated that the nanopore assembly was largely inaccurate for this strain, hence we performed the Illumina sequencing and assembly again for Y22 (Supplementary Table 3).

### RNA-seq experiments and data analysis

We performed two RNA-seq experiments. The first is the analysis of *Dioszegia* EY and PT transcriptome to validate the expression profile of *DhPER1* in vitro. The second experiment is the RNA-seq analysis of *A. laibachii* and *D. hungarica* inoculated on sterile *A. thaliana* leaves at 2 dai. For both experiments, the RNeasy plant mini kit (Qiagen) was used for RNA extraction, and the Turbo DNA-free kit (Thermo Fisher) was used for the removal of the remaining DNA. The purified RNA was then quality controlled by agarose gel electrophoresis and bioanalyzer (Agilent) using the corresponding RNA kit before sending to BMKGene for sequencing.

We performed PacBio long-read RNA sequencing (IsoSeq) for the *D. hungarica* comparative transcriptomics experiment, with one sample for each strain. The circular consensus (CCS) reads from *Dioszegia* EY and PT were received and mapped to their corresponding *DhPER1* paralogues using Geneious RNA mapper and visualized within Geneious Prime (v.2024.0.5). The raw CCS reads from EY and PT were deposited at NCBI SRA under accession ID SRR31480791 and SRR31480792.

For the RNA-seq data from *Albugo*- and *Dioszegia*-inoculated sterile *A. thaliana* at 2 dai, we used Illumina sequencing and performed three biological replicates for each of the three treatment groups (*Albugo*, *Albugo* + EY, *Albugo* + PT). We used a standard RNA-seq analysis workflow^70^ to analyse the data from each treatment group separately. First, the raw Illumina fastq data were quality controlled with FastQC^71^ before trimming with fastp (v.0.24.0)^72^. We then concatenated the genome assembly, transcripts and annotation files of *A. thaliana* (TAIR10)^73^ and *A. laibachii*^19^ before mapping the quality-controlled fastq reads to it with HISAT2 (2.2.0) using default parameters^74^. We assembled the mapped reads into transcripts and calculated the fragments per kilobase of transcript per million read pairs (FPKM) and transcripts per million (TPM) for *A*. *thaliana* and *Albugo* genes using StringTie (2.2.0) with default parameters^75^. Principal component analysis (PCA) was performed on the FPKM of each sample. Similarity among samples was displayed by reducing dimensionality into two or three principal components. Then, we used the ggplot2 R package^65^ for visualizing the PCA of the two most related components. We next used DESeq2 (ref. ^76^) to investigate the differentially expressed *Albugo* genes induced by *D. hungarica*. The list of differentially expressed *Albugo* genes was extracted and submitted to ShinyGO (v.0.82)^77^ for the GO analysis with an FDR cut-off of 0.05 and 4 genes per group selection criteria. The Illumina RNA-seq data were deposited at NCBI SRA under the following accession IDs: SRR31531712–SRR31531717 and SRR31512421–SRR31512423. The intermediate HISAT2 and StringTie output files and main scripts used in this step are available at Scripts_for_data_analysis_and_figure_plotting/RNAseq_DEG_analysis/^60^.

### Structure modelling of *DhPER1* and molecular docking

Structure predictions were conducted with AlphaFold2 (v.2.3.0)^78^ using the full databases for multiple sequence alignment (MSA) construction. We produced five models and selected the best model for visualization purposes. The structure of the yeast thiamine permease *THI7* was downloaded from UniProt^79^. Structures of all permeases (.pdb files) and molecular docking files (.sdf) were posted at /Supplementary_files/Permease structure prediction/. We used PyMOL^80^ for visualization of structures.

### Gene cloning and plasmid construction

We used three plasmids in this study, and they were all amplified in *E. coli* DH5α strain. The CRISPR plasmid (pHSP:CRISPR) contains a high-fidelity variant codon optimized Cas9 gene for *Ustilago maydis*^81^. The single guide RNA (sgRNA) was designed using Geneious Prime (v.2024.0.5) and we chose the target close to the 5’ end of *DhPER1*. The plasmid pHSP:CRISPR was linearized with restriction enzyme Acc65I and assembled with sgRNA oligo and ‘scaffold RNA’ fragment with 3’ downstream 20 bp overlap to the plasmid using Gibson Assembly (NEB) following manufacturer protocol.

The hygromycin resistance gene (HygR) and *DhPER1*-expressing vectors were generated using a shuttle vector EC35 from the fungal Golden Gate system developed for *Ustilago maydis* as backbone^82^. To construct the HygR-expressing vector pHSP:HygR, EC35 was first digested with BamHI and XbaI (NEB), and the HSP::HygR cassette was amplified from the pHSP:CRISPR vector using primer YH67 and YH55. The two fragments were assembled together using Gibson Assembly (NEB) following manufacturer protocol. To generate the pHSP:PER plasmid, we first amplified the HSP70 promoter from the pHSP:CRISPR using YH56 and YH57, and the *DhPER1* transcript (including introns) and terminator from *Dioszegia* EY genomic DNA using YH58 and YH64. We then amplified the HSP::HygR cassette from the pHSP:CRISPR vector using YH54 and YH55. The three fragments were assembled into the same EC35 backbone (BamHI and XbaI digested) using Gibson Assembly (NEB) following manufacturer protocol. We also cloned the *DhPER1* with its endogenous promoter from the *Dioszegia* EY genomic DNA into the same plasmid, but we could not identify any PT transformants showing a clear increase in *DhPER1* expression, indicating that in PT this promoter may not be recognized (Extended Data Fig. 4e). All primers used in this process are summarized in Supplementary Table 7. The plasmid maps were posted at /Supplementary_files/Plasmid map/.

### Genetic modification of *D. hungarica* EY and PT strains

We adapted an electroporation method from *Cryptococcus* to transform *D. hungarica*^83^. Briefly, 100 ml overnight culture of *D. hungarica* was centrifuged at 3,500r.c.f. for 10 min (all centrifugation in this experiment was at 4 °C) and washed 3 times with 10 ml ice-cold water using 3,500 r.c.f. for 7 min. The pellet was then washed with 10 ml electroporation buffer (EB buffer, 10 mM Tris, 1 mM MgCl_2_, 270 mM sucrose, pH7.5) at 3,500r.c.f. for 7 min and resuspended in 10 ml EB buffer with 1 mM dithiothreitol (DTT) on ice for 30 min. The solution was then centrifuged at 3,500r.c.f. for 7 min and resuspended in 1 ml EB buffer to wash out DTT. The pellet was resuspended in 600 μl of EB buffer and stored on ice, ready for electroporation.

For electroporation, 100 μl of freshly prepared competent cells were gently mixed with 2 μg plasmid DNA and loaded in a pre-cooled electroporation cuvette (MicroPulser, Bio-Rad) with a 0.2 cm gap. We then used the GenePulser system (Bio-Rad) to perform the electroporation at 0.45 kV, 125 μF and 500 Ω. The time constant value after electroporation should be 30–37 ms. After electroporation, the solution was streaked on a fresh PDA plate with 100 μg ml^−1^ hygromycin for selection. The colonies were screened using colony PCR and further validated by qPCR on *DhPER1* expression (normalized to *DhEF1α*).

### Visualization of physical interaction between *A. laibachii* zoospores with *D. hungarica*

We first hatched *A. laibachii* zoospores using cold treatment, adapting a method from *Phytophthora* with minor modifications^84^. Briefly, 14-day-old infected plants were placed at 8 °C with high humidity (covered in plastic bag) overnight to induce germination of sporangia. The leaves with clear *A. laibachii* pustules were then collected and soaked in Milli-Q water for 1 h on ice to release sporangia and zoospores. The zoospore solution was filtered with a 40μm filter (Greiner Bio-One) to remove contaminating mycelia. The filtered zoospore solution was kept at 8 °C and checked under a light microscope every 15 min to reach an optimal timepoint with the highest number of free zoospores. We normally found the optimal point around 1 h after spore filtering.

During the waiting time, a small colony from a freshly grown plate of *D. hungarica* was picked with a sterile pipette tip and streaked onto a glass microscope slide. Tape was used to increase the gap between the cover slip and the slide holding *D. hungarica* to allow larger space for zoospore to swim. Around 50 μl of the zoospore solution was pipetted on top of *D. hungarica* and observed under a light microscope. Microscopic pictures and recordings were obtained with a Zeiss AxioPhot (512 colour Axio camera, Carl Zeiss). All pictures and videos were taken under either ×40 or ×64 magnification lens.

### Evolutionary divergence of *DhPER1*

First, an orthologue grouping across the predicted proteome of all *Dioszegia* strains (Supplementary Table 3) was performed using Proteinortho^85^. Only orthogroups of 4 or more members were used in downstream analyses. For each of the orthogroups, codon alignments were constructed by mapping the coding sequence onto its corresponding protein alignment using MAFFT with pal2nal^86,87^. On these, we applied the software PAML to estimate selective pressure^88^, using a combination of four codon substitution models, including null models 1 and 7, and models 2 and 8 allowing for positive selection.

### Phylogeny and selective pressures of permease across the fungal kingdom

The dataset of orthologue *PER* genes across the fungal kingdom was created by searching for homologue genes based on similarity with the ones from *Dioszegia* on the NCBI protein and EGGNOG orthologue databases^89^. After alignment with MAFFT and trimming with trimAl^90^, a maximum-likelihood tree was constructed using IQ-TREE^91^ with default codon model search and 1,000 replicates for ultrafast bootstrap.

We applied the RELAX algorithm^37^ from the HyPhy software suite to test for relaxation pressures in various genera from the PER1 tree. As the testing branches, we used all those immediately leading to each gene, and as background all the branches in the complete tree. Scripts and output files for this part are summarized in Scripts_for_data_analysis_and_figure_plotting/Relax_test_tree/^60^.

### Assessment of translational optimization among *Dioszegia* genomes

The whole-genome phylogeny was calculated on the basis of 2,461 single-copy orthologous protein sequences determined with OrthoFinder (v.2.5.4)^92^. The protein sequences were aligned with MAFFT and the maximum-likelihood phylogeny was calculated on the basis of 500 bootstrap replicates with RAxML (v.8.2.12)^93^. The tRNA genes were predicted with tRNAscan-SE (v.2.0.9)^94^ with default parameters for eukaryotic organisms. Predictions with a covariance score <30 or classified as pseudogenes were excluded from further analyses. The synonymous relative codon usage was calculated with BioKIT (v.0.1.1)^95^. The tRNA adaptation index (tAI) and codon optimization (*S*) were calculated with the *tAI* (v.0.2.1) R package^42^. All scripts and source files used in this part were deposited in Scripts_for_data_analysis_and_figure_plotting/Translational_optimization/.

### Long-term co-cultivation of *A. laibachii* and *D. hungarica* and *Dioszegia* quantifications

Both sterile and non-sterile long-term co-cultivation methods are largely similar to the standard 2-week infection experiments described above, with minor optimizations. For the long-term sterile infection experiment, we used 6-well plates instead of 12-well plates since the volume of media and the size of each well were not sufficient to support the growth of *A. thaliana* for the whole experimental period. Under non-sterile conditions, we sprayed 4–6-week-old *A. thaliana* ws-0 seedlings on 6-well seed germination trays (5 × 5cm) with different strains of *Dioszegia* (OD_600_ = 0.5 in 10 mM MgCl_2_). Each *Dioszegia* strain was sprayed on a total of four 6-well trays, corresponding to 4 sampling timepoints. After 1 h of *Dioszegia* spray or when the liquid evaporated and the *Dioszegia* solutions were settled, the 6-well trays were then sprayed with *A. laibachii* spore solution without antibiotic treatment. We adjusted the spore concentration to 10^4^–10^5^ spores per ml and sprayed two times (~40 μl) per plant. After spraying *A. laibachii*, each 6-well tray was individually bagged with transparent cellophane bags and kept in the dark at 8 °C overnight. The plants were moved to the growth chamber and kept bagged for another 24 h. Infected plants were kept under 8-h/16-h light/dark cycles, 20 °C day and 16 °C night temperature, and 60% humidity.

For sampling of gnotobiotic experiments, the roots were removed and the whole rosette was collected and ground in liquid nitrogen as 1 biological replicate. For each treatment group, we took 6 samples at each time point, so we collected 168 samples in total for each gnotobiotic experiment. We used 100 mg tissue for DNA extraction and qPCR quantification. For plants from the holoxenic experiment, which grew on soil, 3 leaves were collected from 1 plant and 2 plants combined as 1 biological replicate. We took 3 samples for each group at each timepoint, so we collected 96 samples for each holoxenic experiment. Samples were frozen in liquid nitrogen and ground for DNA extraction. DNA was extracted using the DNeasy plant mini kit (Qiagen), qPCR was performed with *D. hungarica* EF1α-specific primer (Supplementary Table 7) and data were normalized to plant biomass using the same experimental setup as for *A. laibachii* quantification described above. We performed this experiment twice and similar results were obtained.

### Reporting summary

Further information on research design is available in the Nature Portfolio Reporting Summary linked to this article.

## Supplementary information

Supplementary Information Supplementary Methods for amplicon data processing and whole-genome sequencing.

Reporting Summary

Peer Review File

Supplementary Tables 1–7 Supplementary Table 1 List of interacting OTUs of *A. laibachii* OTU from oomycete amplicons. Table 2 Summary of all microbial strains used in this study. Table 3 *Dioszegia* genomes used and generated in this study. Table 4 Comparison of the codon optimization index (*S*) in all coding sequences and thiamine-related genes between EY and PT. Table 5 List of thiamine-related genes in *Dioszegia* EY and PT and their percent ranks of tAI in each genome. Table 6 GPS coordinates and climatic variables of each site. Table 7 Oligo nucleotides used in this study.

Supplementary Video 1 *A. laibachii* zoospores interacting with *Dioszegia* EY cells. This and Supplementary Video 2 show a few hatched *A. laibachii* zoospores swimming and making contact with *Dioszegia* EY cells in a dense *Dioszegia* population. The *Dioszegia* EY cells were streaked from a fresh PDA plate, and the hatched *A. laibachii* zoospores (resuspended in water) was dropped on top of the *Dioszegia* cells. The time between frames is 0.167 s, and the video is sped up 10 times at 60 frames per second (fps). Scale bar, 5 µm.

Supplementary Video 2 See Supplementary Video 1 (same experiment).

Supplementary Video 3 *A. laibachii* zoospore interacting with three *Dioszegia* EY cells. The video shows one hatched *A. laibachii* zoospore swimming and making contact with three *Dioszegia* EY cells. The *Dioszegia* EY cells were streaked from a fresh PDA plate, and the hatched *A. laibachii* zoospores (resuspended in water) were dropped on top of the *Dioszegia* cells. The videos were taken under the same condition as Supplementary Videos 1 and 2. Scale bar, 5 µm.

Supplementary Video 4 *A. laibachii* zoospore interacting with one *Dioszegia* EY cell. The video shows a hatched *A. laibachii* zoospore swimming towards a *Dioszegia* EY cell. All videos were taken under the same condition. Scale bar, 5 µm.

Supplementary Video 5 Additional evidence of *A. laibachii* zoospores interacting with *Dioszegia* EY cells at various densities. This and Supplementary Video 6 show a total of eight independent cases of *A. laibachii* zoospores interacting with *Dioszegia* EY cells. All videos were taken under the same condition. Scale bar, 5 µm.

Supplementary Video 6 See Supplementary Video 5 (same experiment).

## Source data

Source Data Fig. 1 Source data for Fig. 1e.

Source Data Fig. 2 Source data for Fig. 2a–c.

Source Data Fig. 3 Source data for Fig. 3b–e.

Source Data Fig. 4 Source data for Fig. 4e.

Source Data Fig. 5 Source data for Fig. 5b and source data and exact *P* values for Fig. 5c.

Source Data Extended Data Fig. 2 Source data for Extended Data Fig. 2b.

Source Data Extended Data Fig. 3 Source data for uncropped gel image of Extended Data Fig. 3b.

Source Data Extended Data Fig. 4 Source data and exact *P* values for Extended Data Fig. 4b–e.

Source Data Extended Data Fig. 8 Source data for Extended Data Fig. 8.

Source Data Extended Data Fig. 9 Source data for Extended Data Fig. 9a–c.

## Data Availability

All raw data were uploaded in the NCBI Sequence Read Archive (SRA) under Bioproject PRJNA1190083 and are available at https://www.ncbi.nlm.nih.gov/bioproject/PRJNA1190083. The accessions for each genome generated in this study are summarized in Supplementary Table 3. Source data are provided with this paper.

## Code availability

All scripts for data analysis and figure plotting were deposited in GitLab at https://gitlab.plantmicrobe.de/yiheng/thiaminecrossfed/(ref. ^60^). The folders referred to in Methods are all in this repository. All scripts and source data for plotting figures are stored in /Scripts_for_data_analysis_and_figure_plotting/scripts_for_figure_plotting/(ref. ^60^) if not otherwise indicated in Methods.
