# Supplementary material for: Thiamine cross-feeding drives microbial coexistence in the leaf microbiome
Source: Nat Microbiol. 2026 Oct 5;11(11):3025–40. doi: 10.1038/s41564-026-02499-w (PMC13739736; doi:10.1038/s41564-026-02499-w)
Supplement: Supplementary file 1 [file 41564_2026_2499_MOESM1_ESM.pdf]

nature microbiology

Supplementary information https://doi.org/10.1038/s41564-026-02499-w

Thiamine cross-feeding drives microbial coexistence in the leaf microbiome

In the format provided by the
authors and unedited

Raw amplicon data processing

The raw data were imported into QIIME2 (version 2023-07)[1] as EMP paired-end sequences. Following, the reads were demultiplexed using the QIIME2 demux plugin. In this step, the sequences were separated for each flow cell by the marker gene (see Amplicon_data_analysis/OTU_table_processing/Metadata/Mapfile_Euro.txt). Reads were processed with the DADA2 pipeline[2] for quality filtering, removal of chimeric and non-biological sequences and generation of representative sequences under the default parameter within the QIIME2 documentation. Reads were further truncated at specific lengths for forward and reverse reads for each taxonomic group. These threshold values were manually selected based on the demultiplexing summaries, ensuring the removal of low-quality and non-informative sequences. After denoising, the sequences from all flow cells were combined. Additionally, the sequences from marker genes coding for the same taxonomic group were summarized and further processed as one set. For that, the sequences were clustered to operational taxonomic units (OTUs) using the *de-novo* clustering option using the vsearch plugin[3] with a percent identity of 97%. The OTUs were further assigned with taxonomic information using a sklearn-based taxonomy classifier[4]. Specifically, for the bacterial group, the pre-trained SILVA classifier (Silva 138, 99% OTUs full-length sequences) provided in QIIME2 package was used[5,6]. For fungi and oomycete reads, the UNITE database (Version 9.0) was used[7]. For other eukaryotic OTUs, the PR2 database (Version 5.0.0) was used[8]. For both UNITE and PR2 databases, taxonomic classifiers were trained with the naive-Bayes classifier trainer[4] under the default settings before being applied to the respective OTU groups. The scripts including all parameters until this point are available under Amplicon_data_analysis/Amplicon_sequencing_processing.

***Dioszegia* whole genome sequencing, assembly and annotation**

The DNA of different *Dioszegia* strains was extracted using DNeasy Plant Mini Kit (Qiagen) for Illumina sequencing or the Puregene Yeast/Bact. Kit (Qiagen) for PacBio and Nanopore sequencing, following manufacturers' protocols. The DNA was sent on dry ice to BMKGene (Biomarker Technologies, Münster, Germany) for sequencing. For all Illumina data, raw fastq files were quality filtered using fastp tool[9] and only reads with Phred score > 30 were retained. The quality filtered fastq was error corrected using Musket[10] and removed PCR duplicates with fastuniq[11]. The quality controlled reads were assembled using SPAdes[12]. For PacBio data,

we used Flye (v2.9.2)[13] with the --pacbio-raw flag to assemble the PacBio data. For nanopore data, we received fastq file basecalled with Guppy (v6, Oxford Nanopore Technologies (ONT), Oxford, UK). We then filtered out reads less than quality score 10 and less than 2000 base pairs with ProwlerTrimmer (ONT). Quality filtered nanopore reads were assembled using canu (v2.2)[14]. The reads used for assembly were then mapped back to the assembled contigs using minimap2[15] and polished with racon[16] for nine times. The racon polished assembly was again polished by medaka (ONT) once using the super high accuracy mode. All assemblies were assessed using BUSCO tool[17] to determine their completeness using the Tremellomycetes_odb10 database. We used the funannotate pipeline[18] for gene prediction using the *Dioszegia hungarica* PDD-24b-2 genome[19] as reference. The predicted genes were functionally annotated using both InterProScan[20] and eggnog-mapper (v2.1.8)[21] under the Galaxy EU platform[22]. The global alignments of the 100kb *DhPER1* and *DhPER2* regions from Fig. 3b and Extended Data Fig. 6d were using clinker[23].

References

1. Bolyen, E. *et al.* Reproducible, interactive, scalable and extensible microbiome data science using QIIME 2. *Nature Biotechnology* **37**, 852–857 (2019).
2. Callahan, B. J. *et al.* DADA2: High-resolution sample inference from Illumina amplicon data. *Nature Methods* **13**, 581–583 (2016).
3. Rognes, T., Flouri, T., Nichols, B., Quince, C. & Mahé, F. VSEARCH: a versatile open source tool for metagenomics. *PeerJ* **4**, e2584 (2016).
4. Pedregosa, F. *et al.* Scikit-learn: Machine Learning in Python. *Journal of Machine Learning Research* **12**, 2825–2830 (2011).
5. Bokulich, N. A. *et al.* Optimizing taxonomic classification of marker-gene amplicon sequences with QIIME 2's q2-feature-classifier plugin. *Microbiome* **6**, 90 (2018).
6. Yilmaz, P. *et al.* The SILVA and "All-species Living Tree Project (LTP)" taxonomic frameworks. *Nucleic Acids Research* **42**, D643–D648 (2014).

7. Abarenkov, K. *et al.* The UNITE database for molecular identification and taxonomic communication of fungi and other eukaryotes: sequences, taxa and classifications reconsidered. *Nucleic Acids Research* **52**, D791–D797 (2024).

8. Guillou, L. *et al.* The Protist Ribosomal Reference database (PR2): a catalog of unicellular eukaryote Small Sub-Unit rRNA sequences with curated taxonomy. *Nucleic Acids Research* **41**, D597–D604 (2013).

9. Chen, S., Zhou, Y., Chen, Y. & Gu, J. fastp: an ultra-fast all-in-one FASTQ preprocessor. *Bioinformatics* **34**, i884–i890 (2018).

10. Liu, Y., Schröder, J. & Schmidt, B. Musket: a multistage k-mer spectrum-based error corrector for Illumina sequence data. *Bioinformatics* **29**, 308–315 (2013).

11. Xu, H. *et al.* FastUniq: A Fast De Novo Duplicates Removal Tool for Paired Short Reads. *PLOS ONE* **7**, e52249 (2012).

12. Bankevich, A. *et al.* SPAdes: A New Genome Assembly Algorithm and Its Applications to Single-Cell Sequencing. *Journal of Computational Biology* **19**, 455 (2012).

13. Kolmogorov, M., Yuan, J., Lin, Y. & Pevzner, P. A. Assembly of long, error-prone reads using repeat graphs. *Nature Biotechnology* **37**, 540–546 (2019).

14. Koren, S. *et al.* Canu: scalable and accurate long-read assembly via adaptive k-mer weighting and repeat separation. *Genome Research* **27**, 722 (2017).

15. Li, H. Minimap2: pairwise alignment for nucleotide sequences. *Bioinformatics* **34**, 3094–3100 (2018).

16. Vaser, R., Sović, I., Nagarajan, N. & Šikić, M. Fast and accurate de novo genome assembly from long uncorrected reads. *Genome Res* **27**, 737–746 (2017).

17. Simão, F. A., Waterhouse, R. M., Ioannidis, P., Kriventseva, E. V. & Zdobnov, E. M. BUSCO: assessing genome assembly and annotation completeness with single-copy orthologs. *Bioinformatics* **31**, 3210–3212 (2015).

18. Palmer, J. M. & Stajich, J. Funannotate v1.8.1: Eukaryotic genome annotation. Zenodo https://doi.org/10.5281/zenodo.4054262 (2020).

19. Jarrige, D. *et al.* High-quality genome of the basidiomycete yeast Dioszegia hungarica PDD-24b-2 isolated from cloud water. *G3 Genes|Genomes|Genetics* **12**, jkac282 (2022).

20. Blum, M. *et al.* The InterPro protein families and domains database: 20 years on. *Nucleic Acids Res* **49**, D344–D354 (2021).

21. Cantalapiedra, C. P., Hernández-Plaza, A., Letunic, I., Bork, P. & Huerta-Cepas, J. eggNOG-mapper v2: Functional Annotation, Orthology Assignments, and Domain Prediction at the Metagenomic Scale. *Mol Biol Evol* **38**, 5825–5829 (2021).

22. Afgan, E. *et al.* The Galaxy platform for accessible, reproducible and collaborative biomedical analyses: 2018 update. *Nucleic Acids Research* **46**, W537 (2018).

23. Gilchrist, C. L. M. & Chooi, Y.-H. clinker & clustermap.js: automatic generation of gene cluster comparison figures. *Bioinformatics* **37**, 2473–2475 (2021).
