# Supplementary material for: Thiamine cross-feeding drives microbial coexistence in the leaf microbiome
Source: Nat Microbiol. 2026 Oct 5;11(11):3025–40. doi: 10.1038/s41564-026-02499-w (PMC13739736; doi:10.1038/s41564-026-02499-w)
Supplement: Supplementary file 2 [file 41564_2026_2499_MOESM2_ESM.pdf]

nature portfolio

Corresponding author(s): Dr. Yiheng Hu and Prof. Dr. Eric Kemen

Last updated by author(s): Jul 27, 2026

Reporting Summary

Nature Portfolio wishes to improve the reproducibility of the work that we publish. This form provides structure for consistency and transparency in reporting. For further information on Nature Portfolio policies, see our Editorial Policies and the Editorial Policy Checklist.

Statistics

For all statistical analyses, confirm that the following items are present in the figure legend, table legend, main text, or Methods section.

n/a	Confirmed	
☐	☒	The exact sample size (*n*) for each experimental group/condition, given as a discrete number and unit of measurement
☐	☒	A statement on whether measurements were taken from distinct samples or whether the same sample was measured repeatedly
☐	☒	The statistical test(s) used AND whether they are one- or two-sided<br>*Only common tests should be described solely by name; describe more complex techniques in the Methods section.*
☐	☒	A description of all covariates tested
☐	☒	A description of any assumptions or corrections, such as tests of normality and adjustment for multiple comparisons
☐	☒	A full description of the statistical parameters including central tendency (e.g. means) or other basic estimates (e.g. regression coefficient) AND variation (e.g. standard deviation) or associated estimates of uncertainty (e.g. confidence intervals)
☐	☒	For null hypothesis testing, the test statistic (e.g. *F*, *t*, *r*) with confidence intervals, effect sizes, degrees of freedom and *P* value noted<br>*Give P values as exact values whenever suitable.*
☒	☐	For Bayesian analysis, information on the choice of priors and Markov chain Monte Carlo settings
☐	☒	For hierarchical and complex designs, identification of the appropriate level for tests and full reporting of outcomes
☒	☐	Estimates of effect sizes (e.g. Cohen's *d*, Pearson's *r*), indicating how they were calculated

Our web collection on statistics for biologists contains articles on many of the points above.

Software and code

Policy information about availability of computer code

Data collection	No software was used for direct data collection.
Data analysis	Data analysis was performed using the following software and packages:<br>Amplicon sequence processing and OTU generation: Raw reads were processed in QIIME2 (v2023.7), using DADA2 for denoising, VSEARCH for de-novo OTU clustering (97% identity), and a scikit-learn naïve-Bayes taxonomy classifier, with the SILVA (v138), UNITE (v9.0) and PR2 (v5.0.0) reference databases. OTU tables were filtered and processed in Python (pandas).<br>Diversity analysis: Alpha diversity (Shannon index) and beta diversity (Bray–Curtis dissimilarities, PERMANOVA with Adonis2) were computed with the R packages vegan and ade4. Taxonomic composition/relative abundance was analysed with phyloseq. Group comparisons used Kruskal–Wallis tests, and results were plotted with ggplot2.<br>Network analysis: Microbial co-occurrence networks were inferred with FastSpar (v0.0.10; SparCC algorithm). Network centrality metrics (degree, betweenness centrality, closeness centrality) were calculated in Cytoscape (v3.9.1), and networks were visualized in Gephi (v0.10.1).<br>Genome assembly and annotation: fastp (v0.24.0), Musket, FastUniq, SPAdes, Flye (v2.9.2), Guppy (v6), ProwlerTrimmer, Canu (v2.2), minimap2, Racon, Medaka, BUSCO (Tremellomycetes_odb10), funannotate, InterProScan and eggNOG-mapper (v2.1.8) on the Galaxy Europe platform. Sequence inspection and curation used Geneious Prime (v2024.0.5).<br>RNA-seq analysis: FastQC and fastp (v0.24.0) for quality control/trimming; HISAT2 (v2.2.0) for read mapping; StringTie (v2.2.0) for transcript assembly and FPKM/TPM quantification; DESeq2 for differential expression; ShinyGO (v0.82) for GO enrichment. PCA and figures were generated with ggplot2.<br>Protein structure prediction and molecular docking: AlphaFold2 (v2.3.0) for structure prediction, PyMOL for visualization, and DiffDock for molecular docking.<br>Evolutionary divergence and selection analyses: Proteinortho and OrthoFinder (v2.5.4) for orthology; MAFFT (with pal2nal) for codon-aware alignment; trimAl for trimming; PAML for dN/dS estimation; IQ-TREE for maximum-likelihood phylogeny; RAxML (v8.2.12) for the whole-

genome maximum-likelihood phylogeny; and the RELAX test (HyPhy suite) for relaxed-selection analysis.
Translational optimization: tRNAscan-SE (v2.0.9) for tRNA gene prediction, BioKIT (v0.1.1) for relative synonymous codon usage, and the tAI R package (v0.2.1) for the tRNA adaptation index and codon optimization (S).
Statistical testing: Mann–Whitney U test, Student's t-test, Tukey's HSD, one-way ANOVA followed by pairwise Welch's t-tests with Benjamini–Hochberg FDR correction, and paired Wilcoxon signed-rank test, performed in R and Python.
Figures: Figures were generated with ggplot2 and Python (pandas, Matplotlib); schematic icons were created with BioRender.

For manuscripts utilizing custom algorithms or software that are central to the research but not yet described in published literature, software must be made available to editors and reviewers. We strongly encourage code deposition in a community repository (e.g. GitHub). See the Nature Portfolio guidelines for submitting code & software for further information.

Data

Policy information about availability of data

All manuscripts must include a data availability statement. This statement should provide the following information, where applicable:

- Accession codes, unique identifiers, or web links for publicly available datasets
- A description of any restrictions on data availability
- For clinical datasets or third party data, please ensure that the statement adheres to our policy

All raw data was uploaded into NCBI short read archive under Bioproject PRJNA1190083 and are available at the following URL: https://www.ncbi.nlm.nih.gov/bioproject/PRJNA1190083. Key intermediate data during the analysis was deposited at https://gitlab.plantmicrobe.de/yiheng/thiaminecrossfed/ under each corresponding section.

All scripts for data analysis and figure plotting were deposited at https://gitlab.plantmicrobe.de/yiheng/thiaminecrossfed/. All scripts and source data for figures plotting were stored in https://gitlab.plantmicrobe.de/yiheng/thiaminecrossfed/Scripts_for_data_analysis_and_figure_plotting/scripts_for_figure_plotting/ if not otherwise indicated in the method.

Research involving human participants, their data, or biological material

Policy information about studies with human participants or human data. See also policy information about sex, gender (identity/presentation), and sexual orientation and race, ethnicity and racism.

Reporting on sex and gender	Not applicable
Reporting on race, ethnicity, or other socially relevant groupings	Not applicable
Population characteristics	Not applicable
Recruitment	Not applicable
Ethics oversight	Not applicable

Note that full information on the approval of the study protocol must also be provided in the manuscript.

Field-specific reporting

Please select the one below that is the best fit for your research. If you are not sure, read the appropriate sections before making your selection.

☒ Life sciences ☐ Behavioural & social sciences ☐ Ecological, evolutionary & environmental sciences

For a reference copy of the document with all sections, see nature.com/documents/nr-reporting-summary-flat.pdf

Life sciences study design

All studies must disclose on these points even when the disclosure is negative.

Sample size	No statistical methods were used to pre-determine sample sizes; sample sizes were chosen based on standard practice in the plant-microbiome and plant-pathogen fields and on our previous work. The sample size of the field survey was referred to previous study: https://www.nature.com/articles/s41559-019-1063-3. 6–10 Arabidopsis thaliana plants were collected per site across the European sampling sites (347 samples in total), split between whole-leaf and endophyte compartments. The sample size for Albugo infection assays were determined by referring to the previous studies: https://elifesciences.org/articles/65306 and https://www.pnas.org/doi/abs/10.1073/pnas.2510124122. For the gnotobiotic and holoxenic infection assays, each treatment group comprised at least 3 (typically 6) biological replicates, with 3–5 plants pooled per replicate; the violin/box plots display all individual data points (n = 21–75 per group, as indicated in each figure legend). These sample sizes were sufficient to detect the reported effects with the statistical tests used.
Data exclusions	We took extensive precautions to ensure reproducibility. For infection assays of obligate biotrophic pathogen Albugo, if overall infection efficiency was poor across all treatments (e.g. remaining antibiotic during the washing steps, or contamination in bad quality spores), the

entire batch was discarded and repeated. Hence, the "data exclusion" mentioned in Methods reflects rigorous quality control, not selective reporting. In total, around one quarter of the infection assays were unsuccessful thus discarded during the whole project.

Replication	All key experiments were independently repeated and the findings were reproducible. The Albugo–Dioszegia infection phenotypes were confirmed across independent biological experiments — for example, the EY/PT infection assay (Fig. 2a) was replicated six times with consistent results, and the long-term co-cultivation experiment was performed twice with similar outcomes. Within each experiment, treatment groups comprised at least 3 (typically 6) biological replicates, and each qPCR data point represents the mean of 3 technical replicates. Observations of the physical Albugo–Dioszegia interaction by microscopy were repeated on multiple independent occasions (documented by the supplementary movies). All attempts at replication were successful.
Randomization	In the controlled experiments, plants were randomly assigned to treatment groups, and the agar plates / growth trays were positioned randomly within the growth chambers to control for position and micro-environment effects. For the field survey, samples were collected from natural A. thaliana populations, so allocation into groups (country/site) was defined by sampling location rather than by experimental assignment.
Blinding	Samples were labelled with codes/numbers during data collection and qPCR quantification to reduce bias, and several experiments were performed and scored independently by more than one researcher. Full blinding of group allocation was not always feasible, because treatments (e.g. the presence/absence of a given microbe) had to be tracked during inoculation and harvesting; however, the principal quantitative read-outs (qPCR biomass and sequencing counts) are objective measurements that are not subject to subjective scoring bias.

Reporting for specific materials, systems and methods

We require information from authors about some types of materials, experimental systems and methods used in many studies. Here, indicate whether each material, system or method listed is relevant to your study. If you are not sure if a list item applies to your research, read the appropriate section before selecting a response.

Materials & experimental systems

n/a	Involved in the study
☒	☐ Antibodies
☒	☐ Eukaryotic cell lines
☒	☐ Palaeontology and archaeology
☒	☐ Animals and other organisms
☒	☐ Clinical data
☒	☐ Dual use research of concern
☐	☒ Plants

Methods

n/a	Involved in the study
☒	☐ ChIP-seq
☒	☐ Flow cytometry
☒	☐ MRI-based neuroimaging

Plants

Seed stocks	A. thaliana ecotype Ws-0 (Wassilewskija, Russia) seeds were produced in greenhouse (ZMBP Tübingen Germany).
Novel plant genotypes	None.
Authentication	not applicable.
