# Supplementary material for: Thiamine cross-feeding drives microbial coexistence in the leaf microbiome
Source: Nat Microbiol. 2026 Oct 5;11(11):3025–40. doi: 10.1038/s41564-026-02499-w (PMC13739736; doi:10.1038/s41564-026-02499-w)
Supplement: Supplementary file 3 [file 41564_2026_2499_MOESM3_ESM.pdf]

nature portfolio

Peer Review File

Thiamine cross-feeding drives microbial coexistence in the leaf microbiome

Corresponding Author: Dr Yiheng Hu

Version 0:

Reviewer comments:

Reviewer #1

(Remarks to the Author)
Review of Hu et al, “A vitamin cross-feeding mechanism mediates microbial niche adaptation on leaves” for Nature Microbiology

This manuscript explores the molecular basis of an interaction between two eukaryotic members of the Arabidopsis thaliana phyllosphere microbiome, the oomycete Albugo laibachii and the yeast Dioszegia hungarica. The authors start with a co-occurrence pattern observed in wild Arabidopsis microbiomes, validate the interaction in the lab with cultured isolates, identify and test a putative mechanism with gene knockouts, and explore its evolutionary relevance using genomic data. They present convincing evidence that there is a direct interpecies interaction between Albugo and Dioszegia that is connected to vitamin auxotrophy, in which certain strains of Dioszegia produce and excrete thiamine that is consumed by Albugo and promotes its colonization. Overall, I think this is a well-conceived and nicely done study that will appeal to a broad readership.
I have some additional questions and suggestions that I hope will improve the clarity of the manuscript.
Introduction: The introduction covers microbiome assembly, obligate biotrophic pathogens such as Albugo, and auxotrophy, but does not mention the yeast Dioszegia that is central to the study. While I realize the narrative starts with seeking potential Albugo partners, one of which turns out to be Dioszegia, I think it would still be helpful to say a bit about Dioszegia. Related to this, I found the sentence on lines 340-341, “Dioszegia is an environmental yeast genus with global distribution,” to be out of place in the midst of the results section.
Line 141: “To assess the role of each genus in the community…” Elsewhere it seems to say Figure 1D is based on OTUs rather than genus (consistent with more than one node being labeled “Albugo” or “Dioszegia”) so does this sentence need to be rephrased?
Line 147 / Figure 1D: “…the yeast Dioszegia showed second-highest betweenness centrality among all fungal OTUs.” What is the bacterium with the highest? Also in relation to the note above, is this just one OTU of the genus Dioszegia?
Figure 1 legend / line 161: “The common environmental yeasts Rhodotorula and Cystofilobasidium isolate were not among the interactors…” Is the word “isolate” needed here?
Line 325: “We collected different Dioszegia strains either from A. thaliana near Tubingen, and included also different type strains…” Should a different plant or location be named, or is “either” unnecessary here?
Figure S6C: I had a hard time understanding the meaning of the K parameter here. The text says “…the Dioszegia branch showing significantly increased purifying selection on this gene (fig. S6C). The evidence for intensified selection further increased when considering exclusively the D. hungarica branch.” But the figure has 3 Dioszegia nodes, which are labeled “Plant-associated Dioszegia” (at K=1), “All Dioszegia” (at K=~1.5) and “Arabidopsis-associated Dioszegia” (at K=~1.9). Are “Arabidopsis-associated Dioszegia” equivalent to D. hungarica? And why would plant-associated show such a dramatically different result than Arabidopsis-associated?
Also in S6, are the lineages in panel A also included in the tree in B? It might be helpful to label those better understand the connections.
Figure S1, line 701: “Each color represents all OTUs from the same country.” I think the points on this plot are samples, not OTUs?
Figure S5, line 760: “..into two or three principal components.” I only see two in the figure.

(Remarks on code availability)
I confirmed the availability of the code at the location cited but there are a lot of scripts and testing them was beyond my ability. While the accuracy of the code is important to the results, I didn't see the software as a key element of the publication.

Reviewer #2

(Remarks to the Author)
<<37134_0_merged_1764394038.pdf>>
Reviewer summary

Hu et al. identify the yeast Dioszegia and oomycete Albugo as a central co-occurring 'hub' pair in European Arabidopsis leaf microbiomes. They provide a reasonably compelling set of findings suggesting thiamine cross-feeding from Dioszegia to Albugo. The evidence for Dioszegia improving Albugo abundance and pathogenesis of Arabidopsis is replicated across experiments and consequently well-supported. Knockout of DhPER1EY - a probable thiamine permease - in Dioszegia decreased Albugo infection rate when co-inoculated on Arabidopsis, while complementation of the mutant with DhPER1EY restored infectivity. Genomic and transcriptomic analysis suggested purifying selection on DhPER1 in Dioszegia and increased expression of DhPER1 in the strain (EY) that promoted Albugo growth and Arabidopsis infectivity relative to the PT strain that did not. qPCR analysis of holozenic (with microbiome) Arabidopsis inoculated with Dioszegia alone or with Albugo suggested that Albugo promoted Dioszegia persistence. Although this evidence appears less conclusive and more context-dependent than for the reciprocal effect. Overall, I believe this is an important study into the interaction mechanism between key 'hub' microbes in the Arabidopsis phyllosphere. Additional clarification or evidence of the positive effect of Albugo on Dioszegia, and the physical mechanism of thiamine transfer would further elevate the work, as specified below:
1) Thiamin transfer from Dioszegia to Albugo is proposed as the mechanism of Dioszegia's positive impact on Albugo's abundance (qPCR) and infectivity on Arabidopsis. I believe this claim could be significantly strengthened if extracellular thiamine concentrations were measured from in vitro cultures of Dioszegia EY and PT strains as well the knockout and complemented EY strains. Microbiological and ELISA assays for thiamine are commercially available and 0.2 micron filtration of cultures should allow separation of the extracellular fraction. This should strengthen the connection between increased expression of DhPER1 and promotion of Albugo infection, especially alongside the thiamine-mediated promotion of Albugo.
2) The claim of a mutualistic interaction where Albugo promotes Dioszegia persistence appears only weakly supported. Could the authors address the following concerns:
a) In gnotobiotic conditions, Albugo suppresses Dioszegia rather than enhance its persistence (Fig S8B). Perhaps the authors could clarify that this appears to be an indirect interaction rather than direct mutualism mentioned in the abstract.
b) The significantly higher Dioszegia DNA in the presence vs absence of Albugo (Figure 5C) appears to only be tested at 32dai, with earlier timepoints removed. A claim of persistence would require a higher bar of more longitudinal measurements, especially considering point d below.
c) Since Albugo reduces plant biomass, the relative increase in Dioszegia DNA (normalized to plant DNA) may be a mathematical artifact of host DNA depletion. Normalization relative to total bacterial/fungal DNA may help to confirm whether Dioszegia is specifically positively impacted relative to other microbes.
d) In Figure 5C it appears that Cohen's d > 0.8 threshold was applied to assign significance letters. However, to my understanding, Cohen's d is a measure of effect size, not a statistical significance test.
i. Please provide results for an omnibus test (e.g. ANOVA).
ii. Specify the post-hoc test and false discovery rate correction used to manage the 21-pairwise comparisons.

Minor comments
Line 34: "Microbial" à "Microbiome" may improve clarity as Microbial homeostasis may suggest cellular homeostasis of individual microbes.
Line 41: "D. hungarica" used here while "Dioszegia" used elsewhere. Consistent use of genus or genus + species name may improve comprehension.
Line 74: What question?
Line 131: Was homogeneity of multivariate dispersion confirmed prior to running Adonis2, e.g. using betadisper?
Line 134: Agaricomycetes don't appear to be the most abundant fungal class in Figure S1C panel 2. Dothideomycetes and Microbotromycetes appear more abundant. Is there an inconsistency here between text and figure?
Line 137-138: Were different filtering parameters for samples and reads tried? Were the results reasonably robust to changes in these parameters?
Line 144-146 and Figure 1D: There appears to be an Oomycete point at ~ (0.62, 0.09). Is this not Albugo but some other oomycete? The lower Albugo label also appears to point to a Eukaryote (grey). Is this just a case of overlapping points? Is the data in this plot fully consistent with the statement "Albugo appeared to be the major hub-taxa for oomycete and eukaryote OTUs, as reflected by theirtop ranking in both closeness centrality and betweenness centrality measures of OTUs within their group".
Figure 1C: adding the shaded ovals to the plot makes it nearly impossible to see the actual data, and the only takeaway appears to be that there is not a strong country level effect. Suggest removal.
Figure 1D: Why are Pseudomonas, Sphingomonas, and Hyaloperonospora labeled? They don't appear to be mentioned in the text or figure legend. Conversely, why are some of the bacteria or fungi with the highest closeness and betweenness centrality not labeled? Further, what is the motivation to focus on the two taxa in particular given the wide distribution of these network metrics? This seems a weak argument overall.
Figure 1D: The text discussing this is a bit obscure. Many of the interactions tested in E show no effect. So, is there any evidence that betweenness centrality and closeness centrality tell us something about the interactions we observe in the gnotobiotic experiments in E? If this were the case, wouldn't we see some correlation between these metrics and the data in E? Is there any evidence for this? If not, what are these network metrics telling us about the interactions?
Figure 1F: It would seem that the point of this panel is to show that the Albugo and the Dioszegia are highly connected. It would be better to present this quantitatively rather than this plot of many lines. Can you make distributions of connectivity and then highlight the location of the two taxa of interest? A node-label permutation test would also be useful to compute the statistical significance of this association vs other pairwise associations.
Figure 2B: I may have missed this, but what is plotted on the x-axis of Figure 2B? This could be made clearer as there are no axis labels, only an axis title. Moreover, why is the number of SNPs per kb a metric for importance of a gene? Many of these SNPs could be synonymous, no?
Figure 2D: Does Diffdock report a confidence score? Could that be shown in figure 2D or reported in the text?
Figure 3C: Is there an underlying reason for the change to a Boxen plot here from the earlier choice of using violin plots for this type of measurement? Would a switch to Boxen plots throughout be beneficial?
Line 312: Is a major reason that all differentially expressed genes in Albugo are upregulated with Dioszegia treatment because the transcript abundances are normalized to all reads from all species, and Albugo increases in abundance with Dioszegia treatment? Would it be insightful to analyze just the transcripts from Albugo to see which genes are relatively up and

downregulated with Dioszegia treatment?
Figure 4D: Confidence intervals on the codon optimization index and a test of statistical significance could help to support this finding of codon optimization in thiamine-related genes.
Figure S9: Additional details in the figure legend would be useful. What does the second question mark over thiamine export to the microbiome indicate? Is the inhibitory arrow from Albugo to the microbiome derived from evidence in this paper or earlier work?
Figure 4E: Would a paired t-test be more appropriate here to test whether the thiamine-related genes have higher translation efficiency in EY when compared with their paired PT counterparts (orthologs)? Visually it appears this may be more significant.
Figure 5C: Please see major comment 2 above.

We hope these comments are helpful to the authors in further strengthening their manuscript.

(Remarks on code availability)

Reviewer #3

(Remarks to the Author)
This manuscript explores a yeast-pathogen metabolic mutualism that promotes their coexistence in the leaf microbiome. First this work demonstrates that both the yeast and the pathogen are keystone taxa within the Arabidopsis leaf microbiome. Based on the finding that some genotypes of the yeast are beneficial to the pathogen and others not, the authors then search for mechanisms. It shows that most likely the production of thiamine by the yeast is responsible for the success of the pathogen. In order to further obtain evidence, the authors also developed an electroporation-based transformation and CRISPR system for the negative Dioszegia EY strain and they included a gene (DhPER1) that favours the pathogen and is linked to thiamine producing genes. This confirmed their hypothesis. Overall, this is a very nice manuscript, with complementary results. I do have various questions.

1) Have the authors tried to grow the yeast and the pathogen together on plates (in media) and explore competition/coexistence between both microbes?
2) What is the yeast receiving from the pathogen? Any metabolite? Albugo had 316 genes that were upregulated when sprayed with Dioszegia EY (the compatible strain) compared to only 20 genes with an incompatible strain. That is interesting. It is still not fully clear to me why Dioszegia would benefit. Is it possible that Albugo provides a direct benefit (a metabolite)? Does any of the upregulated genes points to a potential mechanism (e.g. a metabolite or an effector) that promotes Dioszegia?
3) Can it be excluded that the yeast simply benefits from plant metabolites that are exuded upon Albugo infection of the plant (so an indirect benefit). This should be discussed.
4) Would be possible to measure thiamin production by the different yeast strains? Related to this, it is not fully clear to me which physiological/metabolic changes are caused by insertion of the DhPER1 gene. Is more thiamine produced when this gene is included?

Other (minor) comments

L128-132: perhaps move the description of differences among countries to the supplement as it is not clear this is highly relevant information for the main message of the paper.

L182: The effects of the interactors on Arabidopsis is shown in Figure 2. However, it would be good to show biomass data (not only pictures) and also reveal how each microbe affects Aradopsis biomass and Albugo infection (real data).

L182: it is stated that: “different strains of this genus significantly inhibited Albugo infection under gnotobiotic conditions”. Perhaps specify into: two different strains.

According to Figure 1A the number of Arabidopsis endophytes is: 1069 and the number of leave colonizing microbes is: 1588. This difference appears rather small. Has this also been found in other studies? Please provide rarefaction curves for the sequencing (number of taxa versus sequencing depth).

Figure 5B: Would it be possible to verify the abundance of other microbial strain (e.g. to verify that an increase of other microbes caused a decline in the population of Dioszegia?

(Remarks on code availability)

Reviewer #4

(Remarks to the Author)
Key results
This study identifies a conserved metabolic mutualism between the basidiomycete yeast Dioszegia hungarica and the oomycete Albugo laibachii within the Arabidopsis thaliana leaf microbiome. Using a continental-scale survey combined with targeted co-colonization and genetic assays, the authors provide compelling evidence that thiamine cross-feeding underpins the stable coexistence between these two dominant hub taxa. A central and notable finding is that Dioszegia produces and secretes excess thiamine, which directly facilitates Albugo colonisation, thereby promoting microbiome stability. This work convincingly

links metabolic dependency to ecological prevalence and suggests that nutrient exchange is a key organising principle of leaf microbiomes.

Validity
Overall, the data interpretation is sound, and the conclusions are well supported by the experimental evidence. The authors used appropriate controls throughout, and the logic connecting thiamine biosynthesis, secretion, and mutualistic interactions is clear. I did not identify any flaws that would prevent publication. Some interpretations would benefit from clarification, particularly regarding the apparent persistence of Dioszegia strains lacking DhPER1 during long-term cocultivation, despite the demonstrated importance of this gene for Albugo colonisation. This issue can be resolved through a clearer explanation rather than additional experimentation.

Significance
The conclusions are significant for the plant microbiome field and extend beyond it to microbial ecology and host-associated microbiomes. This study provides rare mechanistic evidence for how metabolic mutualism shapes community structure in natural plant systems. The identification of vitamin cross-feeding as a driver of hub taxon stability has clear implications for microbiome engineering and plant resilience strategies. While previous work has shown that Albugo suppresses host defence and thereby facilitates secondary pathogen colonisation (https://doi.org/10.1094/MPMI-21-6-0745), this manuscript adds a complementary microbial metabolic dimension that strengthens, rather than conflicts with, existing literature. Together, these findings elevate the conceptual importance of this work.

Data and methodology
The experimental approach was appropriate and well executed. The continental survey was carefully designed and provided a strong ecological foundation for the subsequent mechanistic work. The genetic, physiological, and colonisation assays were of high quality, and the data were presented clearly in both the main figures and the supplementary materials. The manuscript included relevant controls and sufficient replication. The overall presentation allowed the reader to follow the progression from field observations to molecular mechanisms without ambiguity.

Analytical approach
The analytical framework is robust and suitable for the questions addressed. The statistical analyses appear appropriate and are applied consistently across datasets. The integration of ecological association data with functional genetic analysis is a particular strength. No aspect of the analytical approach falls outside my expertise, and I did not identify omissions or inappropriate statistical treatment that would undermine the conclusions.

Suggested improvements
All suggested improvements fall well within the current scope of the manuscript. First, the rationale for selecting specific thiamine-related genes, particularly those discussed in lines 212–214, should be explained more explicitly. Second, gene nomenclature requires clarification at the first mention, especially for DhPER1, to avoid confusion with unrelated genes in other systems (PER1 refers to a core circadian clock gene in other organisms). Third, the discussion would benefit from modest expansion to address ecological implications more fully, including how thiamine cross-feeding might interact with Albugo-mediated suppression of plant immunity and broader pathogen dynamics. No additional experiments are strictly necessary.

Clarity and context
The manuscript is clearly written, logically organised, and accessible to a broad readership in plant microbiome research. The figures and tables are well chosen and effectively support the narrative. The contextualisation of the results is generally strong; however, the discussion would benefit from a slightly broader framing that includes obligate downy mildew and related Arabidopsis pathogens, which would reinforce the relevance of the findings. These are issues of emphasis rather than clarity.

References
The manuscript appropriately cites the relevant literature and places the findings within the existing body of work. With the inclusion of a brief discussion of prior studies on Albugo-mediated immune suppression, the reference framework would be fully balanced and comprehensive.

Your expertise
My specialization include plant microbiomes, microbial ecology, and interactions between hosts and microbes, which includes designing experiments, interpreting data, and drawing biological conclusions. I did not thoroughly evaluate the underlying computational code or its implementation, as this is beyond my expertise. Consequently, my assessment of the analyses is confined to their conceptual soundness, biological interpretation and alignment with the results presented.

(Remarks on code availability)

Decision Letter:

4th March 2026

*Please ensure you delete the link to your author homepage in this e-mail if you wish to forward it to your co-authors.

Dear Dr Hu,

Thank you for your patience while your manuscript "A vitamin cross-feeding mechanism mediates microbial niche adaptation on

leaves" was under peer-review at Nature Microbiology. It has now been seen by 4 referees, whose expertise and comments you will find at the end of this email. Although they find your work of potential interest, they have raised a number of concerns that will need to be addressed before we can consider publication of the work in Nature Microbiology.

In particular, Reviewer 2 suggested additional experiments which would further strengthen this work, specifically they ask for measurements of thiamine transfer using in vitro cultures and further clarification of observations made from the gnotobiotic experiments along with longitudinal measurements of Dioszegia. We agree with these suggestion and hope to see them addressed in the revised manuscript. The remaining comments from the reviewers are relatively straightforward and should be easily addressable.

Should further experimental data allow you to address these criticisms, we would be happy to look at a revised manuscript.

We are committed to providing a fair and constructive peer-review process. Please do not hesitate to contact us if there are specific requests from the reviewers that you believe are technically impossible or unlikely to yield a meaningful outcome.

We strongly support public availability of data. Please place the data used in your paper into a public data repository, if one exists, or alternatively, present the data as Source Data or Supplementary Information. If data can only be shared on request, please explain why in your Data Availability Statement, and also in the correspondence with your editor. For some data types, deposition in a public repository is mandatory - more information on our data deposition policies and available repositories can be found at https://www.nature.com/nature-research/editorial-policies/reporting-standards#availability-of-data.

Please include a data availability statement as a separate section after Methods but before references, under the heading "Data Availability". This section should inform readers about the availability of the data used to support the conclusions of your study. This information includes accession codes to public repositories (data banks for protein, DNA or RNA sequences, microarray, proteomics data etc…), references to source data published alongside the paper, unique identifiers such as URLs to data repository entries, or data set DOIs, and any other statement about data availability. At a minimum, you should include the following statement: "The data that support the findings of this study are available from the corresponding author upon request", mentioning any restrictions on availability. If DOIs are provided, we also strongly encourage including these in the Reference list (authors, title, publisher (repository name), identifier, year). For more guidance on how to write this section please see: http://www.nature.com/authors/policies/data/data-availability-statements-data-citations.pdf

If revising your manuscript:

* Include a "Response to referees" document detailing, point-by-point, how you addressed each referee comment. If no action was taken to address a point, you must provide a compelling argument. This response will be sent back to the referees along with the revised manuscript.

* If you have not done so already we suggest that you begin to revise your manuscript so that it conforms to our Article format instructions at http://www.nature.com/nmicrobiol/info/final-submission. Refer also to any guidelines provided in this letter.

* Include a revised version of any required reporting checklist. It will be available to referees (and, potentially, statisticians) to aid in their evaluation if the manuscript goes back for peer review. A revised checklist is essential for re-review of the paper.

When submitting the revised version of your manuscript, please pay close attention to our href="https://www.nature.com/nature-portfolio/editorial-policies/image-integrity">Digital Image Integrity Guidelines. and to the following points below:

-- that unprocessed scans are clearly labelled and match the gels and western blots presented in figures.
-- that control panels for gels and western blots are appropriately described as loading on sample processing controls
-- all images in the paper are checked for duplication of panels and for splicing of gel lanes.

EXTENDED DATA FIGURES

When re-submitting your manuscript, please ensure that any supplementary figures and tables that are crucial to the manuscript's conclusions are converted into Extended Data figures and tables to increase visibility of these data. Extended Data figures and tables are online-only (present in the online PDF and full-text HTML versions of the paper), peer-reviewed display items that provide essential background to the article but are not included in the main article due to space constraints. A maximum of ten Extended Data display items (figures and tables) is permitted.

Finally, please ensure that you retain unprocessed data and metadata files after publication, ideally archiving data in perpetuity, as these may be requested during the peer review and production process or after publication if any issues arise.

Please use the link below to submit a revised paper:

Link Redacted

Note: This url links to your confidential homepage and associated information about manuscripts you may have submitted or be reviewing for us. If you wish to forward this e-mail to co-authors, please delete this link to your homepage first.

Nature Microbiology is committed to improving transparency in authorship. As part of our efforts in this direction, we are now requesting that all authors identified as ‘corresponding author’ on published papers create and link their Open Researcher and Contributor Identifier (ORCID) with their account on the Manuscript Tracking System (MTS), prior to acceptance. This applies to primary research papers only. ORCID helps the scientific community achieve unambiguous attribution of all scholarly contributions. You can create and link your ORCID from the home page of the MTS by clicking on ‘Modify my Springer Nature account’. For more information please visit please visit www.springernature.com/orcid.

If you wish to submit a suitably revised manuscript we would hope to receive it within 6 months.

In the meantime we hope that you find our referees' comments helpful.

Yours sincerely,

****
Reviewer Expertise:

Referee #1: Sequence-based approaches in microbial community assembly, function and dynamics in terrestrial ecosystems
Referee #2: Microbial community structure and function
Referee #3: Agroecology and plant-microbiome interactions
Referee #4: Plant-oomycete interactions

Reviewer Comments:

Reviewer #1 (Remarks to the Author):

Review of Hu et al, “A vitamin cross-feeding mechanism mediates microbial niche adaptation on leaves” for Nature Microbiology

This manuscript explores the molecular basis of an interaction between two eukaryotic members of the Arabidopsis thaliana phyllosphere microbiome, the oomycete Albugo laibachii and the yeast Dioszegia hungarica. The authors start with a co-occurrence pattern observed in wild Arabidopsis microbiomes, validate the interaction in the lab with cultured isolates, identify and test a putative mechanism with gene knockouts, and explore its evolutionary relevance using genomic data. They present convincing evidence that there is a direct interpecies interaction between Albugo and Dioszegia that is connected to vitamin auxotrophy, in which certain strains of Dioszegia produce and excrete thiamine that is consumed by Albugo and promotes its colonization. Overall, I think this is a well-conceived and nicely done study that will appeal to a broad readership.

I have some additional questions and suggestions that I hope will improve the clarity of the manuscript.

Introduction: The introduction covers microbiome assembly, obligate biotrophic pathogens such as Albugo, and auxotrophy, but does not mention the yeast Dioszegia that is central to the study. While I realize the narrative starts with seeking potential Albugo partners, one of which turns out to be Dioszegia, I think it would still be helpful to say a bit about Dioszegia. Related to this, I found the sentence on lines 340-341, “Dioszegia is an environmental yeast genus with global distribution,” to be out of place in the midst of the results section.

Line 141: “To assess the role of each genus in the community…” Elsewhere it seems to say Figure 1D is based on OTUs rather than genus (consistent with more than one node being labeled “Albugo” or “Dioszegia”) so does this sentence need to be rephrased?

Line 147 / Figure 1D: “…the yeast Dioszegia showed second-highest betweenness centrality among all fungal OTUs.” What is the bacterium with the highest? Also in relation to the note above, is this just one OTU of the genus Dioszegia?

Figure 1 legend / line 161: “The common environmental yeasts Rhodotorula and Cystofilobasidium isolate were not among the interactors…” Is the word “isolate” needed here?

Line 325: “We collected different Dioszegia strains either from A. thaliana near Tubingen, and included also different type strains…” Should a different plant or location be named, or is “either” unnecessary here?

Figure S6C: I had a hard time understanding the meaning of the K parameter here. The text says “…the Dioszegia branch showing significantly increased purifying selection on this gene (fig. S6C). The evidence for intensified selection further increased when considering exclusively the D. hungarica branch.” But the figure has 3 Dioszegia nodes, which are labeled “Plant-associated Dioszegia” (at K=1), “All Dioszegia” (at K=~1.5) and “Arabidopsis-associated Dioszegia” (at K=~1.9). Are “Arabidopsis-associated Dioszegia” equivalent to D. hungarica? And why would plant-associated show such a dramatically different result than Arabidopsis-associated?

Also in S6, are the lineages in panel A also included in the tree in B? It might be helpful to label those better understand the connections.

Figure S1, line 701: “Each color represents all OTUs from the same country.” I think the points on this plot are samples, not OTUs?

Figure S5, line 760: “..into two or three principal components.” I only see two in the figure.

Reviewer #1 (Remarks on code availability):

I confirmed the availability of the code at the location cited but there are a lot of scripts and testing them was beyond my ability. While the accuracy of the code is important to the results, I didn't see the software as a key element of the publication.

Reviewer #2 (Remarks to the Author):

<<37134_0_merged_1764394038.pdf>>
Reviewer summary
Hu et al. identify the yeast Dioszegia and oomycete Albugo as a central co-occurring 'hub' pair in European Arabidopsis leaf microbiomes. They provide a reasonably compelling set of findings suggesting thiamine cross-feeding from Dioszegia to Albugo. The evidence for Dioszegia improving Albugo abundance and pathogenesis of Arabidopsis is replicated across experiments and consequently well-supported. Knockout of DhPER1EY - a probable thiamine permease - in Dioszegia decreased Albugo infection rate when co-inoculated on Arabidopsis, while complementation of the mutant with DhPER1EY restored infectivity. Genomic and transcriptomic analysis suggested purifying selection on DhPER1 in Dioszegia and increased expression of DhPER1 in the strain (EY) that promoted Albugo growth and Arabidopsis infectivity relative to the PT strain that did not. qPCR analysis of holozenic (with microbiome) Arabidopsis inoculated with Dioszegia alone or with Albugo suggested that Albugo promoted Dioszegia persistence. Although this evidence appears less conclusive and more context-dependent than for the reciprocal effect. Overall, I believe this is an important study into the interaction mechanism between key ‘hub’ microbes in the Arabidopsis phyllosphere. Additional clarification or evidence of the positive effect of Albugo on Dioszegia, and the physical mechanism of thiamine transfer would further elevate the work, as specified below:
1) Thiamin transfer from Dioszegia to Albugo is proposed as the mechanism of Dioszegia’s positive impact on Albugo’s abundance (qPCR) and infectivity on Arabidopsis. I believe this claim could be significantly strengthened if extracellular thiamine concentrations were measured from in vitro cultures of Dioszegia EY and PT strains as well the knockout and complemented EY strains. Microbiological and ELISA assays for thiamine are commercially available and 0.2 micron filtration of cultures should allow separation of the extracellular fraction. This should strengthen the connection between increased expression of DhPER1 and promotion of Albugo infection, especially alongside the thiamine-mediated promotion of Albugo.
2) The claim of a mutualistic interaction where Albugo promotes Dioszegia persistence appears only weakly supported. Could the authors address the following concerns:
a) In gnotobiotic conditions, Albugo suppresses Dioszegia rather than enhance its persistence (Fig S8B). Perhaps the authors could clarify that this appears to be an indirect interaction rather than direct mutualism mentioned in the abstract.
b) The significantly higher Dioszegia DNA in the presence vs absence of Albugo (Figure 5C) appears to only be tested at 32dai, with earlier timepoints removed. A claim of persistence would require a higher bar of more longitudinal measurements, especially considering point d below.
c) Since Albugo reduces plant biomass, the relative increase in Dioszegia DNA (normalized to plant DNA) may be a mathematical artifact of host DNA depletion. Normalization relative to total bacterial/fungal DNA may help to confirm whether Dioszegia is specifically positively impacted relative to other microbes.
d) In Figure 5C it appears that Cohen’s d > 0.8 threshold was applied to assign significance letters. However, to my understanding, Cohen’s d is a measure of effect size, not a statistical significance test.
i. Please provide results for an omnibus test (e.g. ANOVA).
ii. Specify the post-hoc test and false discovery rate correction used to manage the 21-pairwise comparisons.

Minor comments
Line 34: “Microbial” à “Microbiome” may improve clarity as Microbial homeostasis may suggest cellular homeostasis of individual microbes.
Line 41: “D. hungarica” used here while “Dioszegia” used elsewhere. Consistent use of genus or genus + species name may improve comprehension.
Line 74: What question?
Line 131: Was homogeneity of multivariate dispersion confirmed prior to running Adonis2, e.g. using betadisper?
Line 134: Agaricomycetes don’t appear to be the most abundant fungal class in Figure S1C panel 2. Dothideomycetes and Microbotromycetes appear more abundant. Is there an inconsistency here between text and figure?
Line 137-138: Were different filtering parameters for samples and reads tried? Were the results reasonably robust to changes in these parameters?
Line 144-146 and Figure 1D: There appears to be an Oomycete point at ~ (0.62, 0.09). Is this not Albugo but some other oomycete? The lower Albugo label also appears to point to a Eukaryote (grey). Is this just a case of overlapping points? Is the data in this plot fully consistent with the statement “Albugo appeared to be the major hub-taxa for oomycete and eukaryote OTUs, as reflected by theirtop ranking in both closeness centrality and betweenness centrality measures of OTUs within their group”.
Figure 1C: adding the shaded ovals to the plot makes it nearly impossible to see the actual data, and the only takeaway appears to be that there is not a strong country level effect. Suggest removal.
Figure 1D: Why are Pseudomonas, Sphingomonas, and Hyaloperonospora labeled? They don’t appear to be mentioned in the text or figure legend. Conversely, why are some of the bacteria or fungi with the highest closeness and betweenness centrality not labeled? Further, what is the motivation to focus on the two taxa in particular given the wide distribution of these network metrics? This seems a weak argument overall.
Figure 1D: The text discussing this is a bit obscure. Many of the interactions tested in E show no effect. So, is there any evidence that betweenness centrality and closeness centrality tell us something about the interactions we observe in the gnotobiotic experiments in E? If this were the case, wouldn't we see some correlation between these metrics and the data in E? Is there any evidence for this? If not, what are these network metrics telling us about the interactions?
Figure 1F: It would seem that the point of this panel is to show that the Albugo and the Dioszegia are highly connected. It would be better to present this quantitatively rather than this plot of many lines. Can you make distributions of connectivity and then highlight the location of the two taxa of interest? A node-label permutation test would also be useful to compute the statistical significance of this association vs other pairwise associations.
Figure 2B: I may have missed this, but what is plotted on the x-axis of Figure 2B? This could be made clearer as there are no axis labels, only an axis title. Moreover, why is the number of SNPs per kb a metric for importance of a gene? Many of these SNPs could be synonymous, no?
Figure 2D: Does Diffdock report a confidence score? Could that be shown in figure 2D or reported in the text?
Figure 3C: Is there an underlying reason for the change to a Boxen plot here from the earlier choice of using violin plots for this type of measurement? Would a switch to Boxen plots throughout be beneficial?

Line 312: Is a major reason that all differentially expressed genes in Albugo are upregulated with Dioszegia treatment because the transcript abundances are normalized to all reads from all species, and Albugo increases in abundance with Dioszegia treatment? Would it be insightful to analyze just the transcripts from Albugo to see which genes are relatively up and downregulated with Dioszegia treatment?
Figure 4D: Confidence intervals on the codon optimization index and a test of statistical significance could help to support this finding of codon optimization in thiamine-related genes.
Figure S9: Additional details in the figure legend would be useful. What does the second question mark over thiamine export to the microbiome indicate? Is the inhibitory arrow from Albugo to the microbiome derived from evidence in this paper or earlier work?
Figure 4E: Would a paired t-test be more appropriate here to test whether the thiamine-related genes have higher translation efficiency in EY when compared with their paired PT counterparts (orthologs)? Visually it appears this may be more significant.
Figure 5C: Please see major comment 2 above.

We hope these comments are helpful to the authors in further strengthening their manuscript.

Reviewer #3 (Remarks to the Author):

This manuscript explores a yeast-pathogen metabolic mutualism that promotes their coexistence in the leaf microbiome. First this work demonstrates that both the yeast and the pathogen are keystone taxa within the Arabidopsis leaf microbiome. Based on the finding that some genotypes of the yeast are beneficial to the pathogen and others not, the authors then search for mechanisms. It shows that most likely the production of thiamine by the yeast is responsible for the success of the pathogen. In order to further obtain evidence, the authors also developed an electroporation-based transformation and CRISPR system for the negative Dioszegia EY strain and they included a gene (DhPER1) that favours the pathogen and is linked to thiamine producing genes. This confirmed their hypothesis. Overall, this is a very nice manuscript, with complementary results. I do have various questions.

1) Have the authors tried to grow the yeast and the pathogen together on plates (in media) and explore competition/coexistence between both microbes?
2) What is the yeast receiving from the pathogen? Any metabolite? Albugo had 316 genes that were upregulated when sprayed with Dioszegia EY (the compatible strain) compared to only 20 genes with an incompatible strain. That is interesting. It is still not fully clear to me why Dioszegia would benefit. Is it possible that Albugo provides a direct benefit (a metabolite)? Does any of the upregulated genes points to a potential mechanism (e.g. a metabolite or an effector) that promotes Dioszegia?
3) Can it be excluded that the yeast simply benefits from plant metabolites that are exuded upon Albugo infection of the plant (so an indirect benefit). This should be discussed.
4) Would be possible to measure thiamin production by the different yeast strains? Related to this, it is not fully clear to me which physiological/metabolic changes are caused by insertion of the DhPER1 gene. Is more thiamine produced when this gene is included?

Other (minor) comments

L128-132: perhaps move the description of differences among countries to the supplement as it is not clear this is highly relevant information for the main message of the paper.

L182: The effects of the interactors on Arabidopsis is shown in Figure 2. However, it would be good to show biomass data (not only pictures) and also reveal how each microbe affects Aradopsis biomass and Albugo infection (real data).

L182: it is stated that: “different strains of this genus significantly inhibited Albugo infection under gnotobiotic conditions”. Perhaps specify into: two different strains.

According to Figure 1A the number of Arabidopsis endophytes is: 1069 and the number of leave colonizing microbes is: 1588. This difference appears rather small. Has this also been found in other studies? Please provide rarefaction curves for the sequencing (number of taxa versus sequencing depth).

Figure 5B: Would it be possible to verify the abundance of other microbial strain (e.g. to verify that an increase of other microbes caused a decline in the population of Dioszegia?

Reviewer #4 (Remarks to the Author):

Key results
This study identifies a conserved metabolic mutualism between the basidiomycete yeast Dioszegia hungarica and the oomycete Albugo laibachii within the Arabidopsis thaliana leaf microbiome. Using a continental-scale survey combined with targeted co-colonization and genetic assays, the authors provide compelling evidence that thiamine cross-feeding underpins the stable coexistence between these two dominant hub taxa. A central and notable finding is that Dioszegia produces and secretes excess thiamine, which directly facilitates Albugo colonisation, thereby promoting microbiome stability. This work convincingly links metabolic dependency to ecological prevalence and suggests that nutrient exchange is a key organising principle of leaf microbiomes.

Validity
Overall, the data interpretation is sound, and the conclusions are well supported by the experimental evidence. The authors used appropriate controls throughout, and the logic connecting thiamine biosynthesis, secretion, and mutualistic interactions is clear. I did not identify any flaws that would prevent publication. Some interpretations would benefit from clarification, particularly regarding the apparent persistence of Dioszegia strains lacking DhPER1 during long-term cocultivation, despite the demonstrated importance of this gene for Albugo colonisation. This issue can be resolved through a clearer explanation rather than additional experimentation.

Significance
The conclusions are significant for the plant microbiome field and extend beyond it to microbial ecology and host-associated microbiomes. This study provides rare mechanistic evidence for how metabolic mutualism shapes community structure in natural plant systems. The identification of vitamin cross-feeding as a driver of hub taxon stability has clear implications for microbiome engineering and plant resilience strategies. While previous work has shown that Albugo suppresses host defence and thereby facilitates secondary pathogen colonisation (https://doi.org/10.1094/MPMI-21-6-0745), this manuscript adds a complementary microbial metabolic dimension that strengthens, rather than conflicts with, existing literature. Together, these findings elevate the conceptual importance of this work.

Data and methodology
The experimental approach was appropriate and well executed. The continental survey was carefully designed and provided a strong ecological foundation for the subsequent mechanistic work. The genetic, physiological, and colonisation assays were of high quality, and the data were presented clearly in both the main figures and the supplementary materials. The manuscript included relevant controls and sufficient replication. The overall presentation allowed the reader to follow the progression from field observations to molecular mechanisms without ambiguity.

Analytical approach
The analytical framework is robust and suitable for the questions addressed. The statistical analyses appear appropriate and are applied consistently across datasets. The integration of ecological association data with functional genetic analysis is a particular strength. No aspect of the analytical approach falls outside my expertise, and I did not identify omissions or inappropriate statistical treatment that would undermine the conclusions.

Suggested improvements
All suggested improvements fall well within the current scope of the manuscript. First, the rationale for selecting specific thiamine-related genes, particularly those discussed in lines 212–214, should be explained more explicitly. Second, gene nomenclature requires clarification at the first mention, especially for DhPER1, to avoid confusion with unrelated genes in other systems (PER1 refers to a core circadian clock gene in other organisms). Third, the discussion would benefit from modest expansion to address ecological implications more fully, including how thiamine cross-feeding might interact with Albugo-mediated suppression of plant immunity and broader pathogen dynamics. No additional experiments are strictly necessary.

Clarity and context
The manuscript is clearly written, logically organised, and accessible to a broad readership in plant microbiome research. The figures and tables are well chosen and effectively support the narrative. The contextualisation of the results is generally strong; however, the discussion would benefit from a slightly broader framing that includes obligate downy mildew and related Arabidopsis pathogens, which would reinforce the relevance of the findings. These are issues of emphasis rather than clarity.

References
The manuscript appropriately cites the relevant literature and places the findings within the existing body of work. With the inclusion of a brief discussion of prior studies on Albugo-mediated immune suppression, the reference framework would be fully balanced and comprehensive.

Your expertise
My specialization include plant microbiomes, microbial ecology, and interactions between hosts and microbes, which includes designing experiments, interpreting data, and drawing biological conclusions. I did not thoroughly evaluate the underlying computational code or its implementation, as this is beyond my expertise. Consequently, my assessment of the analyses is confined to their conceptual soundness, biological interpretation and alignment with the results presented.

****

Version 1:

Reviewer comments:

Reviewer #1

(Remarks to the Author)
I appreciate the detailed work the authors put into responding to the reviewers' concerns. I believe all of my questions and concerns have been addressed.

(Remarks on code availability)

Reviewer #2

(Remarks to the Author)
The authors have addressed my concerns satisfactorily. In particular, the new ELISA data substantially strengthen the proposed thiamine-export mechanism, and the revised framing of the Dioszegia–Albugo relationship as context-dependent is more consistent with the data. The additional experiments for Figure 5C and the revised statistical treatment of this data also addresses my concerns and reveals a highly significant result consistent with the earlier figures. A few requested analyses were not performed exactly as suggested, especially the microbial-DNA normalization and the quantitative permutation-based treatment of Fig. 1F, but the authors provide good explanations and have appropriately clarified the associated claims.

Minor comments
Line 243: “b, Scatter plot” Change to ‘Bar plot’.
Line 176: “Dioszegia and Leucosporidium displayed outstanding betweenness centrality among all OTUs”. You might consider also labeling Leucosporidium in Figure 1d, especially considering it is the only other species that has a significant effect on Albugo infectivity (Figure 1e).

(Remarks on code availability)

Reviewer #3

(Remarks to the Author)
The reviewers properly addressed most of my comments (reviewr 3). In their response, it is stated that it cannot be excluded that the yeast simply benefits from plant metabolites that are exuded upon Albugo infection of the plant (so an indirect benefit).

The following answer is given by the authors: We agree with the reviewer that this is a very plausible mechanism and cannot be excluded. The 'modulation of host metabolites' is likely a key component of the indirect benefit Albugo provides to Dioszegia. We have now explicitly incorporated the 'host exudate' hypothesis into our revised Discussion (Paragraph 2) in our response to point (2) above.

This is okay with me. However, the abstract for instance sounds very conclusive. It states: We show that Dioszegia facilitates Albugo colonization by supplying thiamine via a dedicated membrane permease, alleviating Albugo’s auxotrophy. Genomic and transcriptomic analyses reveal that natural selection has acted on thiamine production in Dioszegia,shaping this metabolic complementation.

If the authors are not sure, it is better to soften the conclusions a bit.

(Remarks on code availability)

Reviewer #4

(Remarks to the Author)
I thank the authors for their thorough and careful responses to my comments. All points have been addressed to my satisfaction. The revisions, particularly the expanded discussion on the asymmetric nature of the Dioszegia-Albugo interaction, the clarification of DhPER1 nomenclature, and the broader framing of ecological implications, have strengthened the manuscript. I have no further comments.

Mahmut Tor

(Remarks on code availability)

Decision Letter:

Our ref: NMICROBIOL-25124806A

12th June 2026

Dear Dr. Hu,

Thank you for submitting your revised manuscript "A vitamin cross-feeding mechanism mediates microbial niche adaptation on leaves" (NMICROBIOL-25124806A). It has now been seen by the original referees and their comments are below. The reviewers find that the paper has improved in revision, and therefore we'll be happy in principle to publish it in Nature Microbiology, pending minor revisions to satisfy the referees' final requests and to comply with our editorial and formatting guidelines.

If the current version of your manuscript is in a PDF format, please email us a copy of the file in an editable format (Microsoft

Word or LaTex)-- we can not proceed with PDFs at this stage.

We are now performing detailed checks on your paper and will send you a checklist detailing our editorial and formatting requirements in a couple of weeks. Please do not upload the final materials and make any revisions until you receive this additional information from us.

Thank you again for your interest in Nature Microbiology Please do not hesitate to contact me if you have any questions.

Sincerely,

Reviewer #1 (Remarks to the Author):

I appreciate the detailed work the authors put into responding to the reviewers' concerns. I believe all of my questions and concerns have been addressed.

Reviewer #2 (Remarks to the Author):

The authors have addressed my concerns satisfactorily. In particular, the new ELISA data substantially strengthen the proposed thiamine-export mechanism, and the revised framing of the Dioszegia–Albugo relationship as context-dependent is more consistent with the data. The additional experiments for Figure 5C and the revised statistical treatment of this data also addresses my concerns and reveals a highly significant result consistent with the earlier figures. A few requested analyses were not performed exactly as suggested, especially the microbial-DNA normalization and the quantitative permutation-based treatment of Fig. 1F, but the authors provide good explanations and have appropriately clarified the associated claims.

Minor comments
Line 243: “b, Scatter plot” Change to ‘Bar plot’.
Line 176: “Dioszegia and Leucosporidium displayed outstanding betweenness centrality among all OTUs”. You might consider also labeling Leucosporidium in Figure 1d, especially considering it is the only other species that has a significant effect on Albugo infectivity (Figure 1e).

Reviewer #3 (Remarks to the Author):

The reviewers properly addressed most of my comments (reviewr 3). In their response, it is stated that it cannot be excluded that the yeast simply benefits from plant metabolites that are exuded upon Albugo infection of the plant (so an indirect benefit).

The following answer is given by the authors: We agree with the reviewer that this is a very plausible mechanism and cannot be excluded. The 'modulation of host metabolites' is likely a key component of the indirect benefit Albugo provides to Dioszegia. We have now explicitly incorporated the 'host exudate' hypothesis into our revised Discussion (Paragraph 2) in our response to point (2) above.

This is okay with me. However, the abstract for instance sounds very conclusive. It states: We show that Dioszegia facilitates Albugo colonization by supplying thiamine via a dedicated membrane permease, alleviating Albugo’s auxotrophy. Genomic and transcriptomic analyses reveal that natural selection has acted on thiamine production in Dioszegia,shaping this metabolic complementation.

If the authors are not sure, it is better to soften the conclusions a bit.

Reviewer #4 (Remarks to the Author):

I thank the authors for their thorough and careful responses to my comments. All points have been addressed to my satisfaction. The revisions, particularly the expanded discussion on the asymmetric nature of the Dioszegia-Albugo interaction, the clarification of DhPER1 nomenclature, and the broader framing of ecological implications, have strengthened the manuscript. I have no further comments.

Mahmut Tor

INTERNAL USE ONLY AIP modules required: ["Life Science"]

Version 2:

Decision Letter:

26th August 2026

Dear Dr Hu,

I am pleased to accept your Article "Thiamine cross-feeding drives microbial coexistence in the leaf microbiome" for publication in Nature Microbiology. Thank you for having chosen to submit your work to us and many congratulations.

Over the next few weeks, your paper will be copyedited to ensure that it conforms to Nature Microbiology style. We look particularly carefully at the titles of all papers to ensure that they are relatively brief and understandable.

Once your paper is typeset, you will receive an email with a link to choose the appropriate publishing options for your paper and our Author Services team will be in touch regarding any additional information that may be required. Once your paper has been scheduled for online publication, the Nature press office will be in touch to confirm the details.

You may wish to make your media relations office aware of your accepted publication, in case they consider it appropriate to organize some internal or external publicity. Once your paper has been scheduled you will receive an email confirming the publication details. This is normally 3-4 working days in advance of publication. If you need additional notice of the date and time of publication, please let the production team know when you receive the proof of your article to ensure there is sufficient time to coordinate. Further information on our embargo policies can be found here:
https://www.nature.com/authors/policies/embargo.html

After the grant of rights is completed, you will receive a link to your electronic proof via email with a request to make any corrections within 48 hours. If, when you receive your proof, you cannot meet this deadline, please inform us at rjsproduction@springernature.com immediately. You will not receive your proofs until the publishing agreement has been received through our system

Due to the importance of these deadlines, we ask you please us know now whether you will be difficult to contact over the next month. If this is the case, we ask you provide us with the contact information (email, phone and fax) of someone who will be able to check the proofs on your behalf, and who will be available to address any last-minute problems.

Acceptance of your manuscript is conditional on all authors' agreement with our publication policies (see https://www.nature.com/nmicrobiol/editorial-policies). In particular your manuscript must not be published elsewhere.

Authors may need to take specific actions to achieve compliance with funder and institutional open access mandates. If your research is supported by a funder that requires immediate open access (e.g. according to Plan S principles or the NIH public access policy) then you should select the gold OA route, and we will direct you to the compliant route where possible. Because authors warrant under our subscription licensing terms that they haven’t committed to licensing any version of their article under a licence inconsistent with the terms of our agreement – including the applicable embargo period – publication under the subscription model isn’t suitable for authors whose funders require no embargo.

If you have any questions about our publishing options, costs, Open Access requirements, or our legal forms, please contact ASJournals@springernature.com

An online order form for reprints of your paper is available at https://www.nature.com/reprints/author-reprints.html. All co-authors, authors' institutions and authors' funding agencies can order reprints using the form appropriate to their geographical region.

We welcome the submission of potential cover material (including a short caption of around 40 words) related to your manuscript; suggestions should be sent to Nature Microbiology as electronic files (the image should be 300 dpi at 210 x 297 mm in either TIFF or JPEG format). Please note that such pictures should be selected more for their aesthetic appeal than for their scientific content, and that colour images work better than black and white or grayscale images. Please do not try to design a cover with the Nature Microbiology logo etc., and please do not submit composites of images related to your work. I am sure you will understand that we cannot make any promise as to whether any of your suggestions might be selected for the cover of the journal.

You can now use a single sign-on for all your accounts, view the status of all your manuscript submissions and reviews, access usage statistics for your published articles and download a record of your refereeing activity for the Nature journals.

To assist our authors in disseminating their research to the broader community, our SharedIt initiative provides you with a unique shareable link that will allow anyone (with or without a subscription) to read the published article. Recipients of the link with a subscription will also be able to download and print the PDF.

As soon as your article is published, you will receive an automated email with your shareable link.

With kind regards,

P.S. Click on the following link if you would like to recommend Nature Microbiology to your librarian
http://www.nature.com/subscriptions/recommend.html#forms

** Visit the Springer Nature Editorial and Publishing website at www.springernature.com/editorial-and-publishing-jobs for more information about our career opportunities. If you have any questions please click here.**

Open Access This Peer Review File is licensed under a Creative Commons Attribution 4.0 International License, which permits use, sharing, adaptation, distribution and reproduction in any medium or format, as long as you give appropriate credit to the original author(s) and the source, provide a link to the Creative Commons license, and indicate if changes were made.
In cases where reviewers are anonymous, credit should be given to 'Anonymous Referee' and the source.
The images or other third party material in this Peer Review File are included in the article's Creative Commons license, unless indicated otherwise in a credit line to the material. If material is not included in the article's Creative Commons license and your intended use is not permitted by statutory regulation or exceeds the permitted use, you will need to obtain permission directly from the copyright holder.
To view a copy of this license, visit https://creativecommons.org/licenses/by/4.0/

Reviewer Comments:

Reviewer #1 (Remarks to the Author):

Review of Hu et al, “A vitamin cross-feeding mechanism mediates microbial niche adaptation on leaves” for Nature Microbiology

This manuscript explores the molecular basis of an interaction between two eukaryotic members of the Arabidopsis thaliana phyllosphere microbiome, the oomycete Albugo laibachii and the yeast Dioszegia hungarica. The authors start with a co-occurrence pattern observed in wild Arabidopsis microbiomes, validate the interaction in the lab with cultured isolates, identify and test a putative mechanism with gene knockouts, and explore its evolutionary relevance using genomic data. They present convincing evidence that there is a direct interpecies interaction between Albugo and Dioszegia that is connected to vitamin auxotrophy, in which certain strains of Dioszegia produce and excrete thiamine that is consumed by Albugo and promotes its colonization. Overall, I think this is a well-conceived and nicely done study that will appeal to a broad readership.

We thank the reviewer for the nice summary and positive evaluation of our manuscript.

I have some additional questions and suggestions that I hope will improve the clarity of the manuscript.
Introduction: The introduction covers microbiome assembly, obligate biotrophic pathogens such as Albugo, and auxotrophy, but does not mention the yeast Dioszegia that is central to the study. While I realize the narrative starts with seeking potential Albugo partners, one of which turns out to be Dioszegia, I think it would still be helpful to say a bit about Dioszegia. Related to this, I found the sentence on lines 340-341, “Dioszegia is an environmental yeast genus with global distribution,” to be out of place in the midst of the results section.

We thank the reviewer for this valuable suggestion, and we agree that briefly introducing *Dioszegia* in the Introduction would benefit the readers. We have now added several sentences to the second last paragraph of the Introduction, explaining the rationale for investigating the *Albugo*-interacting taxa, and mentioning *Dioszegia* as an example of stress-tolerant environmental yeasts.

Line 107-114: However, this process is restricted during the early infection stage, as the leaf surface is a challenging environment with limited resources[25,26]. Under such conditions, only a handful of stress-tolerant microbes can persist, such as the globally distributed environmental bacterium *Pseudomonas*[27] and the yeast *Dioszegia*[28]. Whether such co-occurring microbiome members could support auxotrophic obligate biotrophs remains unknown. We hypothesized that specific positive interactions centered around these keystone taxa facilitate their niche co-adaptation and long-term persistence *in planta*, ultimately contributing to the hierarchical structure and stability of the broader microbial community.

We deliberately kept the introduction of *Dioszegia* concise, as the narrative is centred on *Albugo* and an extended introduction of *Dioszegia* at this stage may divert readers from the main storyline. To further strengthen the flow of the Results section, we removed the sentence "*Dioszegia* is an environmental yeast genus with global distribution" and the duplicated introduction of auxotrophy from the opening paragraph of the third Results section, retaining only

the hypothesis statement 'We hypothesized that the metabolic auxotrophy of *Albugo* may be complemented by positive interactors such as *Dioszegia*.'.

Line 141: "To assess the role of each genus in the community…" Elsewhere it seems to say Figure 1D is based on OTUs rather than genus (consistent with more than one node being labeled "Albugo" or "Dioszegia") so does this sentence need to be rephrased?

We apologize for our mistake and thank the reviewer for pointing it out. We have changed the 'genus' to 'microbial OTU'.

Line 147 / Figure 1D: "…the yeast Dioszegia showed second-highest betweenness centrality among all fungal OTUs." What is the bacterium with the highest? Also in relation to the note above, is this just one OTU of the genus Dioszegia?

We thank the reviewer for these questions. The bacterial OTU with the highest betweenness and closeness centrality was *Tardiphaga*. We did not focus on this taxon in the current study as it was predicted to be a negative interactor of *Albugo* (Supplementary Table 1) and we did not have isolates available from our sampling.

The fungal OTU with the highest betweenness centrality was *Cryptococcus* and *Vishniacozyma*. *Cryptococcus* did not show any interaction, and *Vishniacozyma* only showed a weak positive interaction with *Albugo* in the network. We tried *Vishniacozyma* in the gnotobiotic experiment, and it did not promote *Albugo* colonization (Fig. 1e). Although these other taxa represent interesting directions for future study of the *Albugo* interactome, we prioritized the interaction that was both structurally significant in the network and functionally confirmed in planta (*Albugo* and *Dioszegia*).

The 'second-highest' ranking refers to a single OTU of the genus *Dioszegia*. We have revised our language to consistently use 'OTU' rather than 'genus' when discussing these network rankings. To improve the clarity, we also added more description of other labelled OTUs including *Pseudomonas*, *Sphingomonas*, and *Hyaloperonospora*, which are well-known members of the *Arabidopsis* core leaf microbiome. Their high centrality in our European-scale network validates that our model correctly identifies ecologically relevant taxa. We have now clarified this in the Result and Figure Legend:

Line 172-175: Among the most conserved OTUs in endophytic samples, we identified microbial OTUs from genera that are commonly found in the *Arabidopsis* leaf microbiome such as the bacterial genera *Pseudomonas*, *Sphingomonas*, and the oomycetal genus *Hyaloperonospora*[18,33] (Fig. 1d).

Line 151-154: OTUs known to be part of the *A. thaliana* core microbiome (e.g., *Pseudomonas*, *Sphingomonas*) are labeled as reference points to validate network structure, while focal hub taxa (*Albugo*, *Dioszegia*) are prioritized based on their high centrality rankings across sites.

Figure 1 legend / line 161: "The common environmental yeasts Rhodotorula and Cystofilobasidium isolate were not among the interactors…" Is the word "isolate" needed here?

We thank the reviewer for catching this detail. We agree that the word 'isolate' is unnecessary in this context and have removed it from the Fig. 1 legend to improve the clarity and conciseness of the sentence.

Line 325: “We collected different Dioszegia strains either from A. thaliana near Tubingen, and included also different type strains…” Should a different plant or location be named, or is “either” unnecessary here?

We thank the reviewer for pointing out this unclear description. We have now deleted the word ‘either’ from the sentence.

Figure S6C: I had a hard time understanding the meaning of the K parameter here. The text says “…the Dioszegia branch showing significantly increased purifying selection on this gene (fig. S6C). The evidence for intensified selection further increased when considering exclusively the D. hungarica branch.” But the figure has 3 Dioszegia nodes, which are labeled “Plant-associated Dioszegia” (at K=1), “All Dioszegia” (at K=~1.5) and “Arabidopsis-associated Dioszegia” (at K=~1.9). Are “Arabidopsis-associated Dioszegia” equivalent to D. hungarica? And why would plant-associated show such a dramatically different result than Arabidopsis-associated?

We apologize for our unclear description of results and for the ambiguity in our labeling in the Extended Data Fig. 6c. The selection intensification analysis (RELAX) was used to validate if the strong purifying selection of the *DhPER1* gene is unique to the *D. hungarica* lineage, or if it is just a common feature for this gene across all fungi.

The "*Arabidopsis*-associated" branches are indeed equivalent to the *D. hungarica*, while the "Plant-associated" branches represent a separate clade comprising *D. catarinonii* and *D. takashimae*. The dramatic difference in selection intensity between these two lineages likely reflects their distinct ecological niches. The *D. hungarica* lineage is the primary group involved in the thiamine cross-feeding mutualism with the thiamine-auxotrophic hub-taxon *Albugo* in the *Arabidopsis* phyllosphere. In contrast, other plant-associated *Dioszegia* species occupy different niches where this specific mutualism may not be a primary driver of fitness, resulting in selection patterns similar to the background (*K*=1). The genus-level result (*K*=1.5 for ‘All *Dioszegia*) is an average primarily driven by the strong intensification signal within the *D. hungarica* clade.

We further verified that this intensification was a unique feature of the *D. hungarica* lineage rather than an artifact of comparing closely related sequences. We analysed other fungal branches containing *PER1* homologs at a similar taxonomic depth (Blue branches in Fig. S6b, including *Tremella*, *Roridomyces*, and *Rhodotorula*), all of which exhibited relaxed selection (K<1). This result suggests that the intense selection on *DhPER1* is not a general property of the gene or a consequence of comparing closely related sequences, but rather a unique evolutionary signature of *DhPER1* in the *D. hungarica* lineage, likely driven by its specific ecological niche and interaction with *Albugo*.

In response to these points, we have revised the labelling of the *Dioszegia* branches in Extended Data Fig. 6c and highlighted all *Dioszegia* lineages in Extended Data Fig. 6b. Additionally, we have updated the Results and figure legends to improve the clarity of our rationale and the interpretation of these evolutionary findings.

Line 359-366: Using the Relaxed Selection test[43], we observed that this gene has been subject to variable levels of selection across its genealogy, including both relaxation and intensification, with the *Dioszegia* branch showing significantly intensified purifying selection on this gene (K=1.50, Extended Data Fig. 6c). The evidence for intensified selection further increased when considering exclusively the *D. hungarica* branch (K=1.88). In contrast, other fungal clades containing closely related species showed relaxed selection (e.g. *Roridomyces* and *Tremella*, Extended Data Fig. 6c), indicating that this intensified selection is a unique signature of the *D. hungarica* lineage rather than a bias from taxonomic relatedness.

Line 787-791: (c) RELAX analysis of selection intensity (K) across *DhPER1* homologs. Dots in the red section indicate *Dioszegia* lineages, where K>1 represents intensified purifying selection, specifically prominent in the *D. hungarica* clade. Dots in the blue section represent *PER1* homologs in other fungal genera (e.g., *Tremella*, *Roridomyces* and others) at similar taxonomic depths, which exhibit relaxed selection (K<1).

Also in S6, are the lineages in panel A also included in the tree in B? It might be helpful to label those better understand the connections.

We thank the reviewer for the suggestion. We have now labelled all *Dioszegia* branches and highlighted them in Extended Data Fig. 6b.

Figure S1, line 701: "Each color represents all OTUs from the same country." I think the points on this plot are samples, not OTUs?

We thank the reviewer for pointing this out and apologize for our mistake. We have now changed the text 'OTUs' to 'samples'.

Figure S5, line 760: "..into two or three principal components." I only see two in the figure.
We apologize for this mistake. We have now deleted the word 'three' in the legend.

Reviewer #1 (Remarks on code availability):

I confirmed the availability of the code at the location cited but there are a lot of scripts and testing them was beyond my ability. While the accuracy of the code is important to the results, I didn't see the software as a key element of the publication.

We thank the reviewer for verifying the code availability. We appreciate the suggestions provided throughout this review process, which have helped us significantly improve the clarity of the manuscript.

Reviewer #2 (Remarks to the Author):

<<37134_0_merged_1764394038.pdf>>
Reviewer summary
Hu et al. identify the yeast Dioszegia and oomycete Albugo as a central co-occurring 'hub' pair in European Arabidopsis leaf microbiomes. They provide a reasonably compelling set of findings

suggesting thiamine cross-feeding from Dioszegia to Albugo. The evidence for Dioszegia improving Albugo abundance and pathogenesis of Arabidopsis is replicated across experiments and consequently well-supported. Knockout of DhPER1EY - a probable thiamine permease - in Dioszegia decreased Albugo infection rate when co-inoculated on Arabidopsis, while complementation of the mutant with DhPER1EY restored infectivity. Genomic and transcriptomic analysis suggested purifying selection on DhPER1 in Dioszegia and increased expression of DhPER1 in the strain (EY) that promoted Albugo growth and Arabidopsis infectivity relative to the PT strain that did not. qPCR analysis of holozenic (with microbiome) Arabidopsis inoculated with Dioszegia alone or with Albugo suggested that Albugo promoted Dioszegia persistence. Although this evidence appears less conclusive and more context-dependent than for the reciprocal effect. Overall, I believe this is an important study into the interaction mechanism between key 'hub' microbes in the Arabidopsis phyllosphere. Additional clarification or evidence of the positive effect of Albugo on Dioszegia, and the physical mechanism of thiamine transfer would further elevate the work, as specified below:

1) Thiamin transfer from Dioszegia to Albugo is proposed as the mechanism of Dioszegia's positive impact on Albugo's abundance (qPCR) and infectivity on Arabidopsis. I believe this claim could be significantly strengthened if extracellular thiamine concentrations were measured from in vitro cultures of Dioszegia EY and PT strains as well the knockout and complemented EY strains. Microbiological and ELISA assays for thiamine are commercially available and 0.2 micron filtration of cultures should allow separation of the extracellular fraction. This should strengthen the connection between increased expression of DhPER1 and promotion of Albugo infection, especially alongside the thiamine-mediated promotion of Albugo.

We thank the reviewer for this excellent suggestion. We have now performed the ELISA experiment, and we were able to detect significantly higher level of extracellular thiamine from all strains expressing *DhPER1*^{EY}. We have added the ELISA result as Fig. 3c and replaced the original Fig. 3d (the thiamine concentration gradient experiment). We only kept the supplementary movie and removed the images, since they were not mentioned in the result section. Here is the updated result:

Line 307-310: We first assessed the capacity of thiamine exportation in different *Dioszegia* strains with an Enzyme-Linked Immunosorbent Assay (ELISA) *in vitro*. In the thiamine-free yeast nitrogen base medium, all strains expressing *DhPER1*^{EY} exhibited higher extracellular thiamine levels (Fig. 3c), demonstrating that DhPER1 mediates the secretion of thiamine.

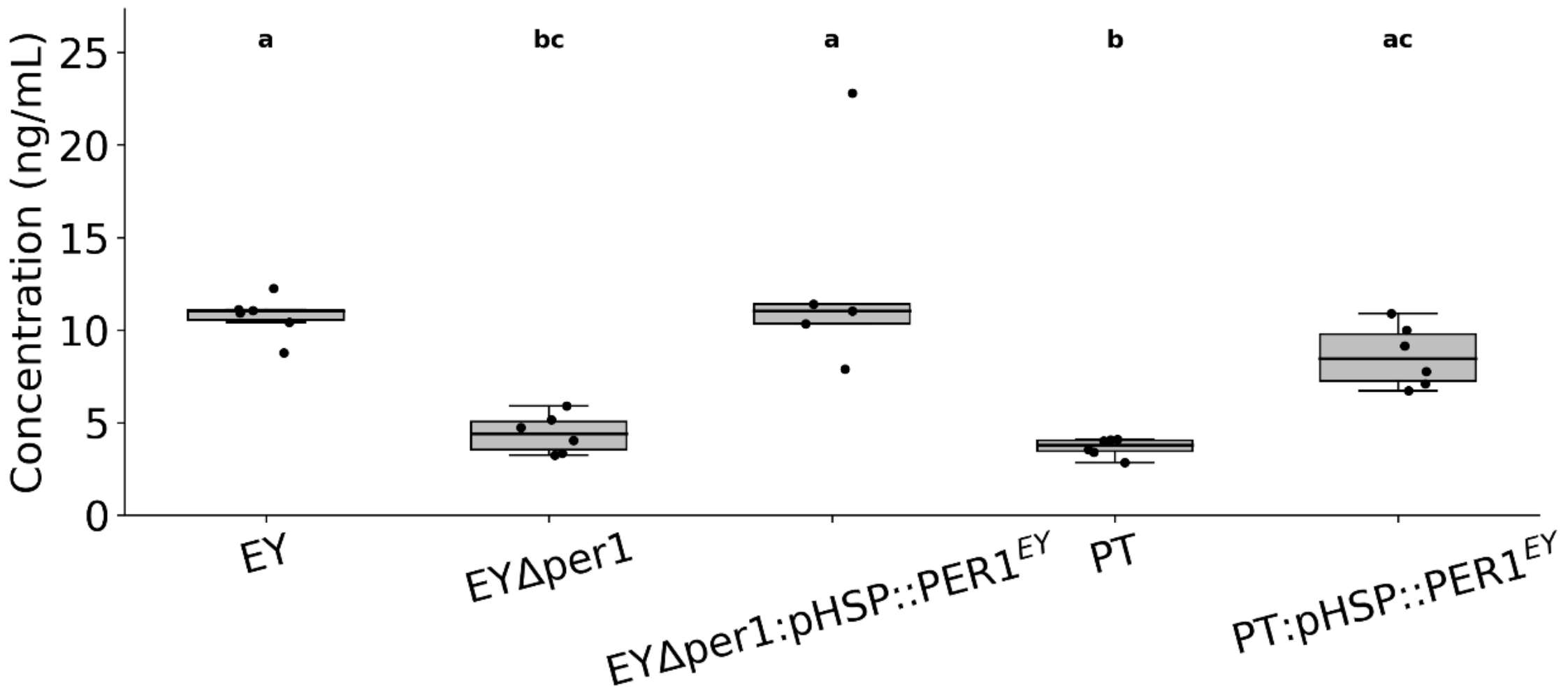

We also added the corresponding method section and uploaded the raw data and data analysis scripts to the Gitlab repository: /Scripts_for_data_analysis_and_figure_plotitng/scripts_for_figure_ploting/scripts_for_figure_plotting_and_statistical_analysis.ipynb

2) The claim of a mutualistic interaction where Albugo promotes Dioszegia persistence appears only weakly supported. Could the authors address the following concerns:
a) In gnotobiotic conditions, Albugo suppresses Dioszegia rather than enhance its persistence (Fig S8B). Perhaps the authors could clarify that this appears to be an indirect interaction rather than direct mutualism mentioned in the abstract.

We thank the reviewer for this insightful comment. We agree that the beneficial effect from *Albugo* to *Dioszegia* is indirect and context-dependent. We have therefore revised the manuscript to describe the relationship more cautiously as an "asymmetric mutualism."

In holoxenic (natural) conditions, *Dioszegia* cannot persist efficiently in the absence of *Albugo*, indicating that *Albugo's* presence is beneficial for its long-term survival. Under gnotobiotic conditions, the interaction shifts toward competition, likely reflecting a trade-off between facilitation and niche competition within a simplified system. We have revised the Discussion to clarify these differences and maintained a more neutral interpretation of the interaction throughout the manuscript.

Line 488-502: Our results also indicate that this mutualistic interaction is context-dependent and characterized by a trade-off between competition and facilitation. Under gnotobiotic conditions, *Albugo* restricts the free growth of *Dioszegia* (Extended Data Fig. 9b), likely reflecting niche competition for limited host resources in a simplified system. However, in a natural ecological context (holoxenic conditions), the relationship shifts toward facilitation. While *Dioszegia* alone faces competitive exclusion by the native microbiota (Fig. 5b), the presence of *Albugo* enables its long-term persistence (Fig. 5c). This 'ecological rescue' suggests that the benefit provided by *Albugo* to *Dioszegia* is primarily indirect, where *Albugo* acts as a niche engineer that modifies the leaf environment—potentially through immune suppression[54], microbial antagonism[22], or modulation of host metabolites exuded during infection[60,61]—to shelter its metabolic partner. The provision of thiamine by *Dioszegia* might fuel the metabolic demands of *Albugo* during these energy-intensive processes of host colonization and immune manipulation. This asymmetric mutualism, combining direct metabolic supply with indirect niche stabilization, indicates that the interaction network extends beyond a single gene-loss dependency and likely involves multiple microbial partners and layered ecological functions.

We also revised the abstract to clarify the 'asymmetric mutualism' concept and rephrased the previous claims on the mutualism:

Line 35-42: Here, we uncover a conserved yeast-oomycete association that promotes their coexistence in the leaf microbiome. Using a continental-scale microbiome survey, we identified an asymmetric mutualistic interaction between two eukaryotic hub microbes: the yeast *Dioszegia hungarica* and the obligate oomycete *Albugo laibachii*. We show that *Dioszegia* facilitates *Albugo* colonization by supplying thiamine via a dedicated membrane permease, alleviating *Albugo*'s auxotrophy. Genomic and transcriptomic analyses reveal that natural selection has acted on thiamine production in *Dioszegia*, shaping this metabolic complementation.

b) The significantly higher Dioszegia DNA in the presence vs absence of Albugo (Figure 5C) appears to only be tested at 32dai, with earlier timepoints removed. A claim of persistence would require a higher bar of more longitudinal measurements, especially considering point d below.

We thank the reviewer for the insightful suggestions. While Fig. 5c only illustrated the data at 32dai, longitudinal data for *Dioszegia* quantification at 0, 12, and 20 dai were included in Extended Data Figures 9b (gnotobiotic) and 9c (holoxenic). To ensure more robust statistical tests, we repeated the long term co-cultivation experiment in the holoxenic condition and re-analyzed all longitudinal measurements.

Specifically, we performed a one-way ANOVA on ΔCq values to determine if *Dioszegia* quantities differed significantly among treatment groups. The results ($F(6, 55) = 48.5$, $p = 3.3e-20$) confirmed significant differences. We subsequently performed pairwise Welch's t-tests with Benjamini–Hochberg false-discovery-rate (FDR) correction for all 21 pairwise comparisons. Consistent with our previous Cohen's d analysis, the results demonstrate that *Dioszegia* strains expressing *DhPER1* maintain significantly higher biomass than non-expressing strains. Interestingly, this more sensitive statistical approach revealed that the *PT* complementation strain (PT:pHSP::PER1^{EY}) showed lower abundance than the *EY* strain in the presence of *Albugo*. This aligns with our findings in Figure 3b, where this specific strain did not promote *Albugo* colonization. We would not like to highlight this result in the main text to maintain the focus on the primary experimental logic, as the underlying mechanism for this observation remains speculative.

We also redesigned Fig. 5b/c and the Extended Data Fig. 9b/c to incorporate the new data and improve clarity. We now use a logarithmic scale for the y-axis, and only used three colors to represent three groups that are significantly different to each other. Here is updated Fig. 5c as an example:

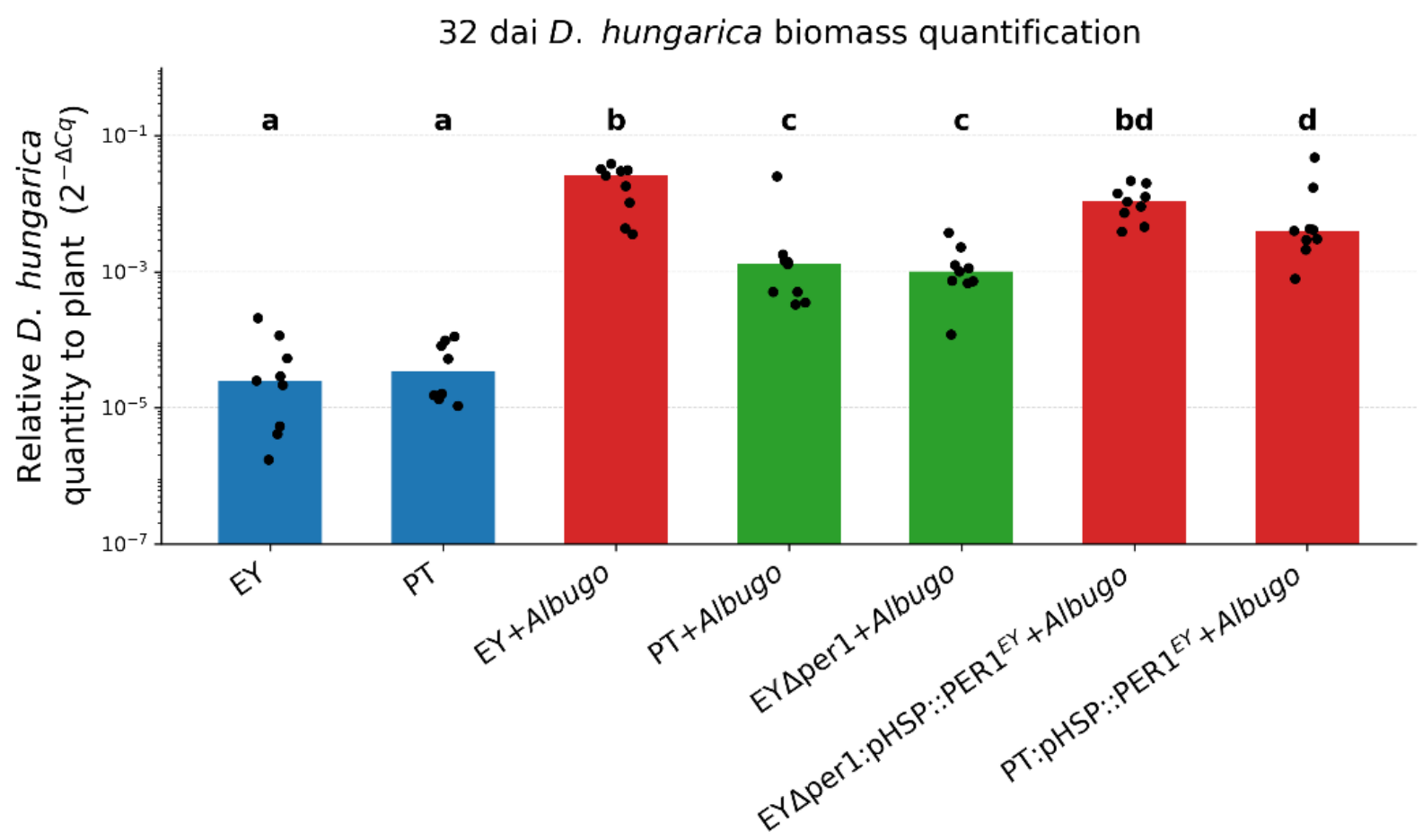

c) Since Albugo reduces plant biomass, the relative increase in Dioszegia DNA (normalized to plant DNA) may be a mathematical artifact of host DNA depletion. Normalization relative to total bacterial/fungal DNA may help to confirm whether Dioszegia is specifically positively impacted relative to other microbes.

We thank the reviewer for the thoughtful consideration of potential mathematical artifacts. To address this, we compared the reduction in host biomass with the increase in *Dioszegia* DNA. While *Albugo* infection reduces plant weight by approximately 50% (Extended Data Fig. 9a), the

corresponding increase in *Dioszegia* is several orders of magnitude higher (60- to 1400-fold). This suggests a true biological promotion rather than an artifact of host DNA depletion.

Regarding the suggested normalization against total microbial DNA, we choose to use host DNA as it is the standard reference for quantifying colonization. Because disease-induced dysbiosis significantly shifts total microbial loads ([Vandeputte et al. 2017, PMID: 29143816]), a microbial baseline can be highly variable between samples. Normalizing against such an inconsistent pool might mask specific ecological effects and increase experimental noise. We therefore believe host DNA provides the most stable and robust comparison for assessing *Dioszegia* persistence across our treatments.

d) In Figure 5C it appears that Cohen's d > 0.8 threshold was applied to assign significance letters. However, to my understanding, Cohen's d is a measure of effect size, not a statistical significance test.
i. Please provide results for an omnibus test (e.g. ANOVA).
ii. Specify the post-hoc test and false discovery rate correction used to manage the 21-pairwise comparisons.
We thank the reviewer for this critique and agree that Cohen's d is a measure of effect size rather than a direct test of statistical significance. We have now replaced that analysis with a more appropriate statistical framework as described in our response to point (b).

(i) We have now provided results for a one-way ANOVA on ∆Cq values (($F(6, 55) = 48.5$, $p = 3.3e-20$) as the omnibus test.

(ii) For post-hoc analysis, we employed pairwise Welch's t-tests across the 21 comparison pairs, applying Benjamini–Hochberg (BH) false-discovery-rate (FDR) correction to account for multiple testing.

While the statistical methods have been updated, the core biological conclusions remain consistent with the previous version. We therefore updated the statistical test result (Line 459) in the last Result paragraph, and rewrote the figure legend:

Line 441-450: b, Biomass of *Dioszegia* EY and PT strains under gnotobiotic and holoxenic conditions during a 32-day time course without *Albugo*. qPCR analysis was used for biomass quantification. Each data point shows the median plus standard error of *Dioszegia* biomass from 3-9 plants, and each plant sample contains three technical replicates. c, Biomass of different *Dioszegia* strains under holoxenic conditions at 32 dai. Bars show the median across biological replicates. Each dot represents a biological replicate, calculated as the median of three technical replicates. Different colors indicate different experiment groups (-*Albugo*, +*DhPER, -DhPER*). Group medians were compared by one-way ANOVA on ΔCq ($F(6, 55) = 48.5$, $p = 3.3e-20$), followed by pairwise Welch's t-tests on the 21 condition pairs with Benjamini–Hochberg false-discovery-rate (FDR) correction. Groups sharing the same letter are not significantly different (FDR < 0.05). In both (b) and (c) the *DhEF1a* gene is used for qPCR and normalized to the *EF1a* gene from *A. thaliana.*

We also updated the raw Cq data for the holoxenic long term co-cultivation experiment in the Gitlab repository and the corresponding figure plotting/statistical analysis scripts: Scripts_for_data_analysis_and_figure_plotitng/scripts_for_figure_ploting/scripts_for_figure_plotting_and_statistical_analysis.ipynb

Minor comments
Line 34: “Microbial” à “Microbiome” may improve clarity as Microbial homeostasis may suggest cellular homeostasis of individual microbes.

We thank the reviewer for this suggestion. We have now changed the wording to ‘microbiome homeostasis’.

Line 41: “D. hungarica” used here while “Dioszegia” used elsewhere. Consistent use of genus or genus + species name may improve comprehension.

This is a good point. We have now changed all names to ‘Dioszegia’ in the abstract.

Line 74: What question?

We have revised the sentence at Line 73 to more explicitly indicate the open question: "...the extent to which microbe–microbe interactions contribute to host-associated microbiome assembly remains unclear’, which provides a clear antecedent for "This question" in the following sentence.

Line 131: Was homogeneity of multivariate dispersion confirmed prior to running Adonis2, e.g. using betadisper?

We thank the reviewer for this important suggestion. We tested homogeneity of multivariate dispersion using betadisper, and it was found to be significant for all datasets (p= 0.0139 for endophytes and p= 0.0042 for whole leaf). These results indicate that the significant differences identified by PERMANOVA (Adonis2) reflect a combination of both location effects (shifts in community centroids) and dispersion effects (differences in within-group variation). This heterogeneity is likely a biological feature of our large-scale geographical dataset, where different regions exhibit varying levels of complexity in the microbiome.

We have now added the betadisper result to the Extended Data Fig. 1 (c&d), and revised the Results section as follows:

Line 134-139: However, beta-diversity analyses, including principal coordinate analysis and multivariate dispersion tests, revealed significant differences in microbial community composition and within-region variation across the four geographical regions (Fig. 1c, Extended Data Fig. 1b-d). These results align with previous reports of host biogeography explaining microbial variation[10,29], and provide a diverse ecological context for distinguishing universal microbial hubs from site-specific variation.

Line 134: Agaricomycetes don’t appear to be the most abundant fungal class in Figure S1C panel 2. Dothideomycetes and Microbotromycetes appear more abundant. Is there an inconsistency here between text and figure?

We thank the reviewer for pointing this out and sincerely apologize for the mistake. We have now re-examined the raw data and we identified that the labeling of the figure was mistaken. We have now updated the figure with as new Extended Data Fig. 1g. The updated script were deposited at the Gitlab repository:

Scripts_for_data_analysis_and_figure_plotitng/scripts_for_figure_ploting/scripts_for_figure_plotting_and_statistical_analysis.ipynb

Line 137-138: Were different filtering parameters for samples and reads tried? Were the results reasonably robust to changes in these parameters?

We thank the reviewer for raising this point. We have tested different filtering on the minimum number of samples for each OTU, and our results are robust based on two aspects: 1. The ratio between positive and negative correlations; 2. The ratio between number of observed edges and all possible edges (Density). Here is a summary of the results:

Min. samples per OTU	Positive edges (%)	Negative edges (%)	Density (%)
5	52.86%	47.14%	29.52%
10	49.68%	50.32%	33.69%
13	49.26%	50.74%	34.67%
20	49.70%	50.30%	36.53%

We chose the threshold 10 since it is consistent with several other thresholds (13/20), and it also retains the higher number of OTUs for further analysis.

Regarding read filtering, we applied a threshold of 50 total reads across the entire dataset. We chose this specific value to remain consistent across all four taxonomic groups (bacteria, fungi, oomycetes, and eukaryotes). A lower threshold (e.g., 20 reads) risked the inclusion of sequencing artifacts that could create spurious correlations, while a higher threshold (e.g., 100 reads) would have been too stringent for the less abundant eukaryotes and oomycetes, potentially removing biologically relevant taxa. Given that our results were robust to sample-prevalence changes, we used this standard 50-read filter to maintain consistency across different taxonomic groups.

Line 144-146 and Figure 1D: There appears to be an Oomycete point at ~ (0.62, 0.09). Is this not Albugo but some other oomycete? The lower Albugo label also appears to point to a Eukaryote (grey). Is this just a case of overlapping points? Is the data in this plot fully consistent with the statement "Albugo appeared to be the major hub-taxa for oomycete and eukaryote OTUs, as reflected by theirtop ranking in both closeness centrality and betweenness centrality measures of OTUs within their group".

We thank the reviewer for this detailed observation. The oomycete point mentioned at (0.62, 0.09) corresponds to a Pythium OTU. While *Pythium* exhibits high closeness centrality in this dataset, it is primarily a soil-borne root pathogen. Therefore, we prioritized *Albugo* for further analysis due to its established role as a specialized leaf pathogen.

The lower *Albugo* label is from a eukaryote OTU. Among all eukaryote OTUs, *Albugo* ranked sixth in centrality. The only taxa ranking higher are either unclassified (3) or belong to the genera *Hyaloperonospora* and *Adineta*. Together with its position among oomycete OTUs, these results confirm that *Albugo* is among the most influential and conserved taxa in the phyllosphere. We have slightly revised the text to more accurately reflect its position:

Line 177-180: Across all samples, two OTUs classified as *Albugo* appeared to be among the most prominent hub-taxa among leaf-resident oomycete and eukaryote OTUs, as reflected by their high rankings in both closeness centrality and betweenness centrality measures of OTUs within their group (Fig. 1d).

We agree that the interpretation of the network analysis requires further detail, and we provide additional clarification regarding the prioritization of *Albugo* in our responses to the specific comments on Fig. 1d below.

Figure 1C: adding the shaded ovals to the plot makes it nearly impossible to see the actual data, and the only takeaway appears to be that there is not a strong country level effect. Suggest removal.

We thank the reviewer for this suggestion. We have now removed the shaded oval from Fig. 1c and Extended Data Fig. 1b. We have also revised the related text in the result section which included additional variation resulting in the significant variation of the beta-diversity (Line 134-139).

Figure 1D: Why are Pseudomonas, Sphingomonas, and Hyaloperonospora labeled? They don't appear to be mentioned in the text or figure legend. Conversely, why are some of the bacteria or fungi with the highest closeness and betweenness centrality not labeled? Further, what is the motivation to focus on the two taxa in particular given the wide distribution of these network metrics? This seems a weak argument overall.

We thank the reviewer for this critique, which has helped us clarify the logic regarding the interpretation of Fig. 1d and the subsequent prioritization of focal taxa.

The labels for *Pseudomonas*, *Sphingomonas*, and *Hyaloperonospora* were included to serve as reference points. As these are well-known members of the *Arabidopsis* core leaf microbiome, their high centrality in our European-scale network validates that our model correctly identifies ecologically relevant taxa. We have now clarified this in the Result and Figure Legend:

Line 172-174: Among the most conserved OTUs in endophytic samples, we identified microbial OTUs from genera that are commonly found in the *Arabidopsis* leaf microbiome such as the bacterial genera *Pseudomonas*, *Sphingomonas*, and the oomycetal genus *Hyaloperonospora*[18,33] (Fig. 1d).

Line 151-154: OTUs known to be part of the *A. thaliana* core microbiome (e.g., *Pseudomonas*, *Sphingomonas*) are labeled as reference points to validate network structure, while focal hub taxa (*Albugo*, *Dioszegia*) are prioritized based on their high centrality rankings across sites.

We apologize for the unclear logic flow about the selection of *Albugo* and *Dioszegia*. In our revised manuscript, we have clarified that our prioritization followed a two-step approach:

Step 1: Our initial focal microbe based on the network analysis was only *Albugo*, and we focused more on *Dioszegia* after the functional test with gnotobiotic experiment (Fig. 1e). We have now revised the result section and only highlighted the central role of *Albugo* from the interpretation of Fig. 1d:

Line 172-182: Among the most conserved OTUs in endophytic samples, we identified microbial OTUs from genera that are commonly found in the *Arabidopsis* leaf microbiome such as the bacterial genera *Pseudomonas*, *Sphingomonas*, and the oomycetal genus *Hyaloperonospora*[18,33] (Fig. 1d). We also observed that several OTUs from basidiomycete yeasts *Dioszegia* and *Leucosporidium* displayed outstanding betweenness centrality among all OTUs, indicating their connecting role within the microbiome[34]. Across all samples, two OTUs classified as *Albugo* appeared to be among the most prominent hub-taxa among leaf-resident oomycete and

eukaryote OTUs, as reflected by their high rankings in both closeness centrality and betweenness centrality measures of OTUs within their group (Fig. 1d). Given its global prevalence and capacity for asymptomatic persistence in the phyllosphere[15–19], our result validated the central organizing positions of *Albugo* within the *A. thaliana* leaf microbiome.

Step 2: To identify partners for *Albugo*, we prioritized the network's top-ranking interactors for experimental testing. This included the fungal genera with the highest betweenness centrality, *Leucosporidium* and *Vishniacozyma* (Supplementary Table 1). Our initial hypothesis focused on *Leucosporidium*. However, while *Leucosporidium* was predicted to be a strong positive interactor *in silico*, multiple strains inhibited *Albugo* in planta (Fig. 1e). In contrast, *Dioszegia*—which was also a high-ranking hub—demonstrated robust promotion of *Albugo*.

By emphasizing *Dioszegia* only after its functional confirmation, we aim to provide a clearer narrative. We also mentioned in the Discussion that we need to combine both approaches to fully understand the microbial interactions within microbiome (Line 502-505).

Regarding the specific question about the OTUs with the highest centralities, they were *Tardiphaga* and *Vishniacozyma*. We did not mention *Tardiphaga* since we focus more on the positive interactors of *Albugo* (*Tardiphaga* is a negative interactor as shown in Supplementary Table 1), and we did not have good strain collection. We tested *Vishniacozyma* in the gnotobiotic experiment but it does not promote *Albugo* and the interaction predicted with *Albugo* is weak. We would be grateful to follow up these results and investigate other directions of the *Albugo* interactome, but for this manuscript we thought to keep the storyline more straightforward.

Figure 1D: The text discussing this is a bit obscure. Many of the interactions tested in E show no effect. So, is there any evidence that betweenness centrality and closeness centrality tell us something about the interactions we observe in the gnotobiotic experiments in E? If this were the case, wouldn't we see some correlation between these metrics and the data in E? Is there any evidence for this? If not, what are these network metrics telling us about the interactions?

We agree that the 'predicted interaction' are often inconsistent with what we observed from the gnotobiotic experiment. We have now strengthened the logic and expanded the discussion regarding the discrepancies between the network analysis and the gnotobiotic experiments:

1. We primarily used network metrics as a discovery filter to identify "hub" taxa from a dataset containing thousands of OTUs. As noted in the result section (Line 169-172), high betweenness centrality indicates that a microbe serves as a 'bridge' in the community. This could be due to many reasons: metabolic cross-feeding, immune modulation, or simply occupying a common niche. Therefore, not every positive interactor of *Albugo* predicted from network analysis is expected to promote its colonization, and some might be indirect interaction which require additional microbe not present in our gnotobiotic assay. We revised the Result section (Line 200-204) to highlight this conclusion:

Line 200-204: This result underscores the challenge in translating *in silico* microbial associations into direct functional outcomes[35]. While network centrality metrics identify hub taxa from thousands of OTUs, inferred microbial interactions can be context-dependent and may require additional environmental or microbial factors to manifest in simplified pairwise assays[26,36].

2. Network correlations are non-directional, whereas biological interactions are often asymmetric. For example, obligate biotrophic pathogens such as *Albugo* can facilitate opportunistic microbes

indirectly through immune suppression or leaking nutrient from infected plant tissue (resulting in a positive correlation). However, those microbes do not necessarily provide a reciprocal benefit to *Albugo*. Our study specifically sought microbes that directly support *Albugo* (Line 185), and we found such positive feedbacks are critical for niche co-adaptation and long-term community stability. In this description of Fig. 1, we would prefer to not deviate the readers into the other direction of interaction, and primarily focus on the microbes directly benefiting *Albugo*. To further clarify this asymmetric *Dioszegia-Albugo* interaction, especially the indirect benefit from *Albugo* to *Dioszegia*, we revised Discussion section as mentioned above at major concern #2 (a) (Line 488-502).

Figure 1F: It would seem that the point of this panel is to show that the Albugo and the Dioszegia are highly connected. It would be better to present this quantitatively rather than this plot of many lines. Can you make distributions of connectivity and then highlight the location of the two taxa of interest? A node-label permutation test would also be useful to compute the statistical significance of this association vs other pairwise associations.

We thank the reviewer for this comment. Our rationale for this panel was to validate in detail that *Albugo* and *Dioszegia* are interconnected at the single OTU level, and more importantly, they are all very well-connected within the whole community. This sub-network shows that *Albugo* and *Dioszegia* are not only central members of the community, but they may also directly interact with each other. Because mathematical significance in a network does not always match biological outcomes—as seen with *Leucosporidium*—we believe this panel is most useful as a qualitative confirmation of the links we chose to test experimentally. We have revised the Result section to clarify this purpose:

Line 209-213: **To validate the interconnectivity of all *Dioszegia* and *Albugo* OTUs as hub taxa and to establish their interactions, we extracted all direct interactions of *Albugo* and *Dioszegia* OTUs the whole leaf network (Fig. 1f). We found three direct connections between *Albugo* and *Dioszegia* OTUs, with additional indirect links through other microbes. Both taxa maintained extensive connections to the broader community, reinforcing their positions as central hubs.**

In summary, Figure 1d identifies *Albugo* as a key organizer of the microbiome; Figure 1e provides an experimental screen showing that the hub taxon *Dioszegia* promotes *Albugo* colonization; and Figure 1f validates that these two microbes are directly linked in whole leaf microbiome data.

We sincerely thank the reviewer again for helping us tighten the logic flow among Fig. 1d-f, which lays the foundation for further investigation into the crossfeeding mechanism.

Figure 2B: I may have missed this, but what is plotted on the x-axis of Figure 2B? This could be made clearer as there are no axis labels, only an axis title. Moreover, why is the number of SNPs per kb a metric for importance of a gene? Many of these SNPs could be synonymous, no?

We thank the reviewer for this suggestion. We have now changed the Fig. 2b and the relevant Extended Data Fig. 3a to a bar plot for clearer illustration of the results. The corresponding code is also updated in the Gitlab repository:
Scripts_for_data_analysis_and_figure_plotitng/scripts_for_figure_ploting/scripts_for_figure_plotting_and_statistical_analysis.ipynb

While dN/dS is a common metric for selection, we chose to present SNP density and AA variation separately to provide a transparent view of the absolute degree of divergence. We think this approach could better support *DhPER1* as the primary candidate based on its unique standing as a high-divergence outlier at both the DNA and protein levels, which correlated with its striking differential expression (Fig 2c).

Figure 2D: Does Diffdock report a confidence score? Could that be shown in figure 2D or reported in the text?

We apologize for the omission of this detail. The confidence score was recorded in the raw data, which we have now uploaded in the github repository (Supplementary_files/Permease_structure_prediction). We have also added the confidence score in the main text (Line260) and the figure legend of Extended Data Fig. 3f. While the confidence scores of the best binding models are moderate, we consider this to be biologically and technically reasonable given the inherent challenges of *in silico* docking using AlphaFold-predicted structures of dynamic membrane proteins. Despite these constraints, the consistent localization of the ligand in the predicted binding pocket provides valuable support for the functional role of DhPER1.

Figure 3C: Is there an underlying reason for the change to a Boxen plot here from the earlier choice of using violin plots for this type of measurement? Would a switch to Boxen plots throughout be beneficial?

We thank the reviewer for this suggestion. We chose a boxen plot for Fig. 3c since this dataset contains several zero groups, and boxen plot provides better illustration for it. Changing others figures about infection assay to boxen plot does not look better than violin plot, especially for Fig. 1e, which contain many treatment groups. We prefer to keep this plotting style and only use boxen plot for Fig. 3c.

Line 312: Is a major reason that all differentially expressed genes in Albugo are upregulated with Dioszegia treatment because the transcript abundances are normalized to all reads from all species, and Albugo increases in abundance with Dioszegia treatment? Would it be insightful to analyze just the transcripts from Albugo to see which genes are relatively up and downregulated with Dioszegia treatment?

We thank the reviewer for raising this important methodological detail. The potential for compositional bias (where an increase in the biomass of one species shifts the relative abundance of all its transcripts) is a well-known challenge in multi-species RNA-seq. We have carefully validated our results and believe our normalization strategy remains the most appropriate for this dataset for the following reasons:

1. Early sampling minimizes biomass shifts:
We collected samples at the zoospore releasing stage (2 days after inoculation). At this early time point, we do not expect much increase in *Albugo* biomass.

2. Stability of Housekeeping Genes:
To validate if the results were driven by biomass differences, we examined seven conserved *Albugo* housekeeping genes: EF1-α (ALNC14_047230), 60S ribosomal proteins L44/L37a (ALNC14_052750/054140), 40S ribosomal protein S4 (ALNC14_052900), α/β-Tubulin

(ALNC14_062890/040420), and Actin (ALNC14_062060). Compared to the buffer control, all of them do not show difference in expression in either EY or PT treated samples, excepting one gene (β-Tubulin) slightly upregulated in both yeast treatments. This confirms that the observed upregulation of 316 genes is a targeted biological response, not a global biomass artifact.

3. Specificity of the response:
Out of approximately 13,000 genes in the *Albugo* genome, only 316 (~2.4%) were significantly upregulated. If the results were driven by a biomass-induced normalization artifact, we would expect a global upward shift across the transcriptome, which is not what we observe.

4. Technical robustness in low-coverage data:
At 2 dai, *Albugo* transcripts represent a very small fraction of the total library. Despite generating >9Gb of data per replicate, *Albugo* transcriptome coverage remains relatively low (10-20X). In such "sparse" datasets, normalizing exclusively to the *Albugo* read pool (intra-species normalization) can significantly amplify stochastic noise and sequencing biases. Normalizing to the total transcript pool provides a more stable baseline and reduces within-group variation among replicates (as seen in Fig. 3e).

We have updated the GitLab repository (RNAseq_DEG_analysis) to include full gene annotations.

Figure 4D: Confidence intervals on the codon optimization index and a test of statistical significance could help to support this finding of codon optimization in thiamine-related genes.

We thank the reviewer for this suggestion. The codon optimization index S is a single value that represents the co-adaptation between the tRNA gene pool and the codon usage bias of a given dataset, and further accounts for the effective number of codons and GC content at the 3rd position. As such, confidence intervals based on sampling theory are not applicable to S. We have instead added an additional statistical comparison of the tRNA adaptation index (tAI) values between thiamine biosynthesis genes and the full genomic CDS background (new Extended Data Fig. 8). Thiamine genes show significantly higher normalized translation efficiency than the genomic average in both species (Wilcoxon test: EY, *p* = 0.0026; PT, *p* = 0.015), providing statistical support for the elevated codon optimization observed in this gene set.

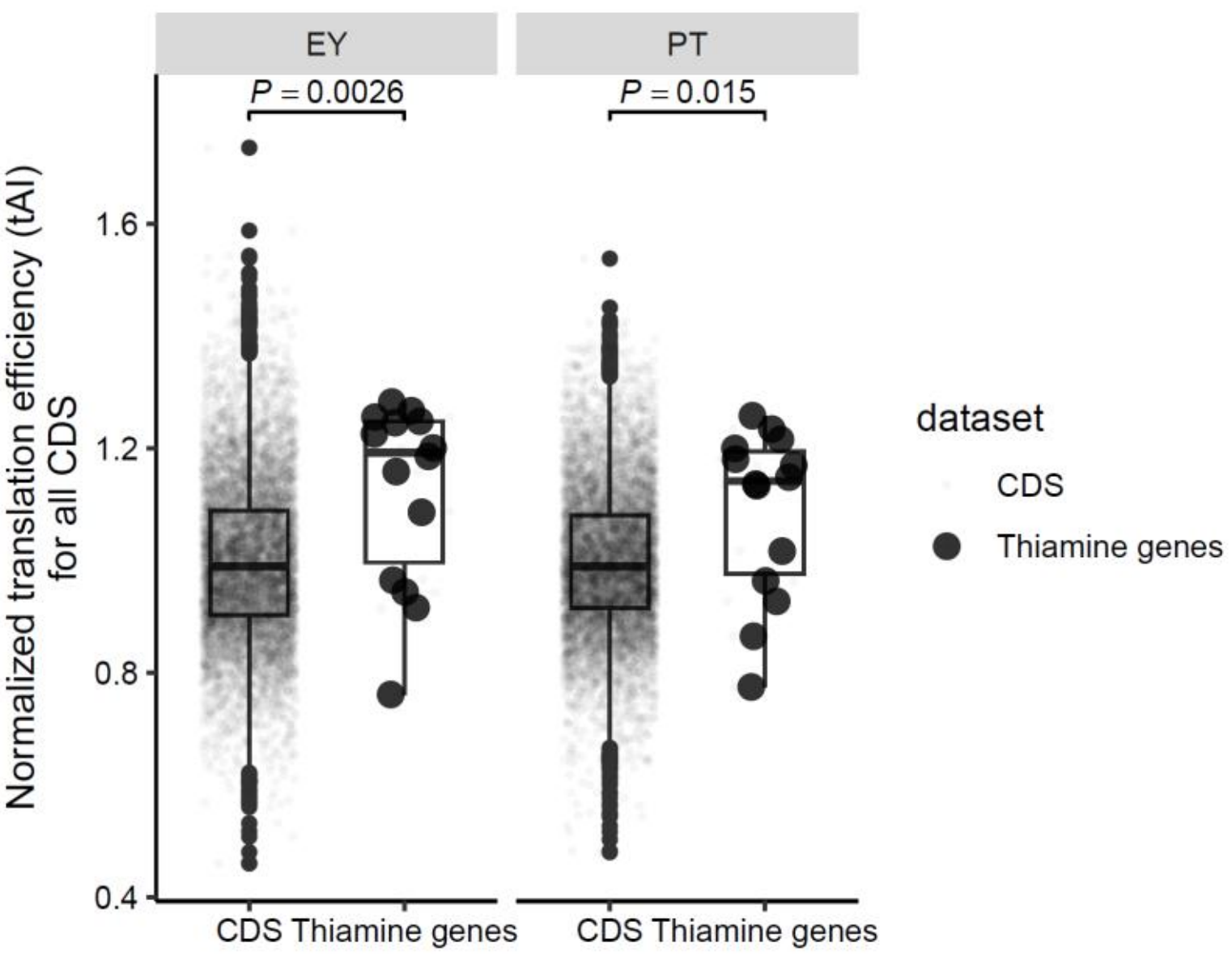

We also revised the corresponding Result section:

Line 416-419: In both EY and PT genomes, thiamine-related genes showed significantly higher translation efficiency (tAI) compared to the average for the rest of the genome (Extended Data Fig. 8). Notably, EY had significantly higher tAI values for these genes, including *DhPER1*, than PT (Fig 4e, Supplementary Table 4, paired Wilcoxon *p*=0.0017), indicating translational optimization correlated with the cross-feeding mechanism.

Figure S9: Additional details in the figure legend would be useful. What does the second question mark over thiamine export to the microbiome indicate? Is the inhibitory arrow from Albugo to the microbiome derived from evidence in this paper or earlier work?

We thank the reviewer for this suggestion and apologize for the incomplete description of this figure. We have now added more details to the figure legend:

Line 816-823: Extended Data Fig. 10. Working model of *Dioszegia-Albugo* interactions in an ecological context. *Dioszegia* exports thiamine via the permease DhPER1, which is taken up by *Albugo* through an uncharacterized membrane transporter (question mark in *Albugo* cell). The only thiamine-related gene in the *Albugo* genome encodes a thiamine pyrophosphokinase (TPTase) that irreversibly converts free thiamine into thiamine pyrophosphate — a bioactive form that cannot exit the cell, effectively sequestering the thiamine acquired from *Dioszegia*. A second question mark indicates the uncertain extent to which secreted thiamine may be utilized by other phyllosphere microbes. The inhibitory arrow from *Albugo* to the microbiome reflects previous findings that *Albugo* selectively suppresses certain phyllosphere microbes[22]. The figure was generated using BioRender.

Figure 4E: Would a paired t-test be more appropriate here to test whether the thiamine-related genes have higher translation efficiency in EY when compared with their paired PT counterparts (orthologs)? Visually it appears this may be more significant.

We thank the reviewer for this suggestion. As the compared genes are orthologs, a paired Wilcoxon signed-rank test is indeed more appropriate. We report a paired Wilcoxon test rather than a paired t-test given the small sample size (n = 14 pairs), which shows a significant difference (paired wilcoxon *p* = 0.00170). We have revised the Fig. 4e and the corresponding text (Line 416-420), and the significant difference actually could support our conclusion better.

Figure 5C: Please see major comment 2 above.

We hope these comments are helpful to the authors in further strengthening their manuscript.
We sincerely thank the reviewer again for the diligent evaluation of our manuscript, which significantly strengthened the logic and the conclusion of this study.

Reviewer #3 (Remarks to the Author):

This manuscript explores a yeast-pathogen metabolic mutualism that promotes their coexistence in the leaf microbiome. First this work demonstrates that both the yeast and the pathogen are keystone taxa within the Arabidopsis leaf microbiome. Based on the finding that some genotypes of the yeast are beneficial to the pathogen and others not, the authors then search for mechanisms. It shows that most likely the production of thiamine by the yeast is responsible for the success of the

pathogen. In order to further obtain evidence, the authors also developed an electroporation-based transformation and CRISPR system for the negative Dioszegia EY strain and they included a gene (DhPER1) that favours the pathogen and is linked to thiamine producing genes. This confirmed their hypothesis. Overall, this is a very nice manuscript, with complementary results. I do have various questions.
We thank the reviewer for the nice summary and the positive evaluation of our manuscript.

1) Have the authors tried to grow the yeast and the pathogen together on plates (in media) and explore competition/coexistence between both microbes?

We thank the reviewer for this suggestion. Since *Albugo* is an obligate pathogen which is not culturable, we need to perform all the experiment on their natural host *Arabidopsis.*

2) What is the yeast receiving from the pathogen? Any metabolite? Albugo had 316 genes that were upregulated when sprayed with Dioszegia EY (the compatible strain) compared to only 20 genes with an incompatible strain. That is interesting. It is still not fully clear to me why Dioszegia would benefit. Is it possible that Albugo provides a direct benefit (a metabolite)? Does any of the upregulated genes points to a potential mechanism (e.g. a metabolite or an effector) that promotes Dioszegia?

We thank the reviewer for this insightful question and suggestion. Identifying the specific molecular return benefit from *Albugo* to *Dioszegia* remains an intriguing question in our ongoing research. We re-examined the 316 *Albugo* genes upregulated by *Dioszegia* EY and could not identify a clear pathway suggesting a direct metabolite "gift" to the yeast. Interestingly, one gene involved in terpenoid biosynthesis was significantly upregulated (~5.5 fold) under EY treatment but not PT, which could be related to the synthesis of signalling compounds or the niche modification in general. However, these 316 genes primarily reflect *Albugo*'s own metabolic activation and preparation for infection, rather than a dedicated mechanism for nutrient export. We have updated the GitLab repository (RNAseq_DEG_analysis) and included full gene annotations of each DEG.

Our analysis is also constrained by the existing *Albugo* functional annotations, as over one-third of the upregulated genes (107 genes) are classified as hypothetical or unnamed proteins. We are conducting a separate project using long-read sequencing and Hi-C technology to generate a more complete, well-annotated *Albugo laibachii* reference genome. We will re-visit this transcriptomic dataset once this improved resource is available to search for novel factors that may specifically facilitate the yeast partner.

Given that *Albugo* restricts yeast growth in sterile conditions (Extended Data Fig. 8b) but rescues it in the presence of a native microbiome (Fig. 5c), we agree with the reviewer that the return benefit is primarily indirect. We argue that this benefit represents an "ecological rescue" mediated by *Albugo* acting as a niche engineer that modulates host metabolites and suppresses competing microbiota to stabilize its metabolic partner. We have now revised the Discussion to clarify this point:

Line 488-505: Our results also indicate that this mutualistic interaction is context-dependent and characterized by a trade-off between competition and facilitation. Under gnotobiotic conditions, *Albugo* restricts the free growth of *Dioszegia* (Extended Data Fig. 9b), likely reflecting niche competition for limited host resources in a simplified system. However, in a natural ecological context (holoxenic conditions), the relationship shifts toward facilitation. While *Dioszegia* alone

faces competitive exclusion by the native microbiota (Fig. 5b), the presence of *Albugo* enables its long-term persistence (Fig. 5c). This 'ecological rescue' suggests that the benefit provided by *Albugo* to *Dioszegia* is primarily indirect, where *Albugo* acts as a niche engineer that modifies the leaf environment—potentially through immune suppression[54], microbial antagonism[22], or modulation of host metabolites exuded during infection[60,61]—to shelter its metabolic partner. The provision of thiamine by *Dioszegia* might fuel the metabolic demands of *Albugo* during these energy-intensive processes of host colonization and immune manipulation. This asymmetric mutualism, combining direct metabolic supply with indirect niche stabilization, indicates that the interaction network extends beyond a single gene-loss dependency and likely involves multiple microbial partners and layered ecological functions. This interaction complexity opens an intriguing area of fundamental research, highlighting the needs to combine computational and experimental approaches to fully understand the direct and indirect metabolic interactions within microbiome.

3) Can it be excluded that the yeast simply benefits from plant metabolites that are exuded upon Albugo infection of the plant (so an indirect benefit). This should be discussed.

We agree with the reviewer that this is a very plausible mechanism and cannot be excluded. The 'modulation of host metabolites' is likely a key component of the indirect benefit *Albugo* provides to *Dioszegia*. We have now explicitly incorporated the 'host exudate' hypothesis into our revised Discussion (Paragraph 2) in our response to point (2) above.

4) Would be possible to measure thiamin production by the different yeast strains? Related to this, it is not fully clear to me which physiological/metabolic changes are caused by insertion of the DhPER1 gene. Is more thiamine produced when this gene is included?

We thank the reviewer for this excellent suggestion. As also detailed in our response to Reviewer 2 (Concern 1), we performed an ELISA experiment to quantify the extracellular thiamine concentration across different *Dioszegia* strains using a thiamine-free Yeast Nitrogen Base (YNB) medium (updated Fig. 3c). Strains with functional *DhPER1* (EY, EY∆per1:pHSP::PER1^{EY}, PT:pHSP::PER1^{EY}) exhibited significantly higher level of thiamine in the medium than the strains without functional *DhPER1* (PT, EY∆per1), suggesting that the DhPER1 mediates the secretion of thiamine. We observed no significant differences in yeast growth rates in thiamine-free media, likely because *Dioszegia* is a thiamine autotroph. This supports our conclusion that *DhPER1* primarily affects the capacity to "share" thiamine with the environment.

We have updated the manuscript as follows:

Line 307-310: We first assessed the capacity of thiamine exportation in different *Dioszegia* strains with an Enzyme-Linked Immunosorbent Assay (ELISA) *in vitro*. In the thiamine-free yeast nitrogen base medium, all strains expressing *DhPER1*^{EY} exhibited higher extracellular thiamine levels (Fig. 3c), demonstrating that DhPER1 mediates the secretion of thiamine.

The Methods section (Line 223-242) and GitLab repository have been updated accordingly.

Other (minor) comments

L128-132: perhaps move the description of differences among countries to the supplement as it is not clear this is highly relevant information for the main message of the paper.

We thank the reviewer for this suggestion. We put the description of differences among countries in the main figure (Fig. 1b,c) since we would like to show that our dataset covers large diversity while maintains enough depth, so that the 'conserved' microbial interactions is ecologically relevant. We have now revised the result section and emphasized this rationale:

Line 137-139: These results align with previous reports of host biogeography explaining microbial variation[11,25], and provide a diverse ecological backdrop for distinguishing universal microbial hubs from site-specific variation.

L182: The effects of the interactors on Arabidopsis is shown in Figure S2. However, it would be good to show biomass data (not only pictures) and also reveal how each microbe affects Aradopsis biomass and Albugo infection (real data).

We thank the reviewer for this suggestion. We have now performed another gnotobiotic plant experiment to assess the effect of different microbes on the *Arabidopsis* biomass. The two filamentous fungi (*Fusarium* and *Stagonosporopsis*) and one yeast (*Sporobolomyces*) significantly reduced the biomass of *Arabidopsis*. Specifically, *Fusarium* completely killed the plant after 12 days as shown in the previous Fig. S2c, so we did not include it in the test with *Albugo*. Most other microbes tested together with *Albugo* do not significantly impact the *Arabidopsis* biomass. We have now updated the Extended Data Fig. 2b as bellow:

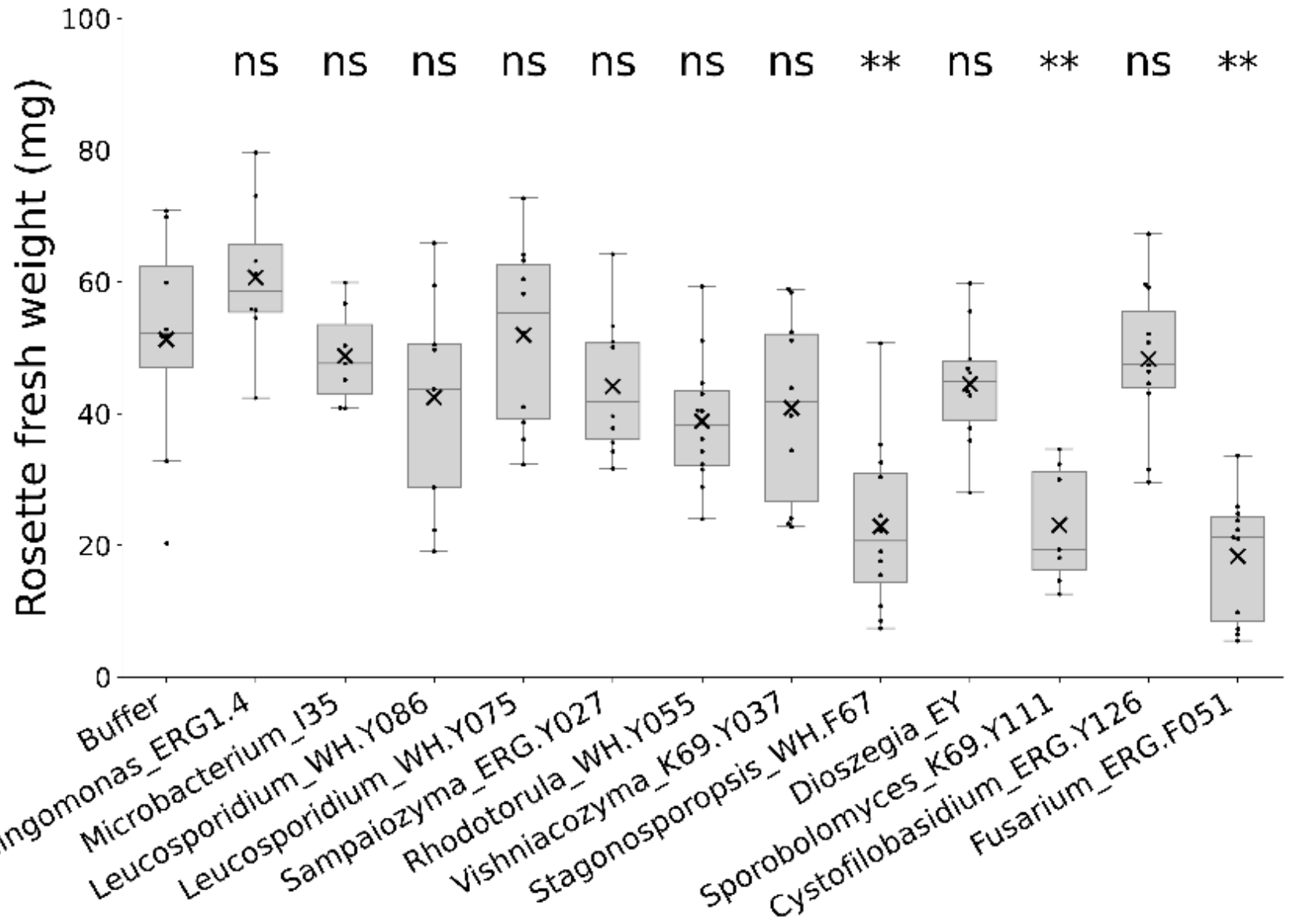

We also updated the result section:

Line 196-198: We found that most of the *Albugo*-interacting microbes were either pathogenic to *A. thaliana* or affected neither *A. laibachii* infection nor *Arabidopsis* biomass (Fig. 1e, Extended Data Fig. 2b).

L182: it is stated that: "different strains of this genus significantly inhibited Albugo infection under gnotobiotic conditions". Perhaps specify into: two different strains.

We thank the reviewer for this suggestion, which improved the clarity of our manuscript. We have now changed the sentence accordingly.

According to Figure 1A the number of Arabidopsis endophytes is: 1069 and the number of leave colonizing microbes is: 1588. This difference appears rather small. Has this also been found in other studies? Please provide rarefaction curves for the sequencing (number of taxa versus sequencing depth).

We thank the reviewer for this question regarding the OTU counts. In our experimental design, 'whole leaf' samples represent the total community (epiphytes plus endophytes), while 'endophyte' samples consist of the subset remaining after surface sterilization. The observed 1.5-fold difference in OTU richness (1,588 vs. 1,069) is also consistent with previous studies, where epiphytic diversity is typically 1 to 2-fold compared to endophytic diversity (Yao et al., 2019: PMID: 30967154; Gomes et al., 2018: PMID: 29500493, Ashhab et al., 2021: PMID: 34322096).

This OTU table processing pipeline is also adapted from a previous similar study of *Arabidopsis* root microbiome (Thiergart et al., 2020, PMID: 31900452), which did not include sample rarefaction. This is because only samples containing OTUs from all four taxonomic groups are kept, and the sequencing depth of each taxonomic group varies significantly among different samples. Therefore, the rarefaction based on the dataset from one taxonomic group would remove many samples containing meaningful datasets in other taxonomic groups. Instead, we performed a strict 7-step filtering of the OTU table and kept most samples for the network analysis to identify hub microbes (See Materials and Methods). For the reviewer's information, we attached the rarefaction curve after the OTU filtering step and added it in our analysis script of the Gitlab repository：/Scripts_for_data_analysis_and_figure_plotitng/Amplicon_data_analysis/OUT_table_processing.

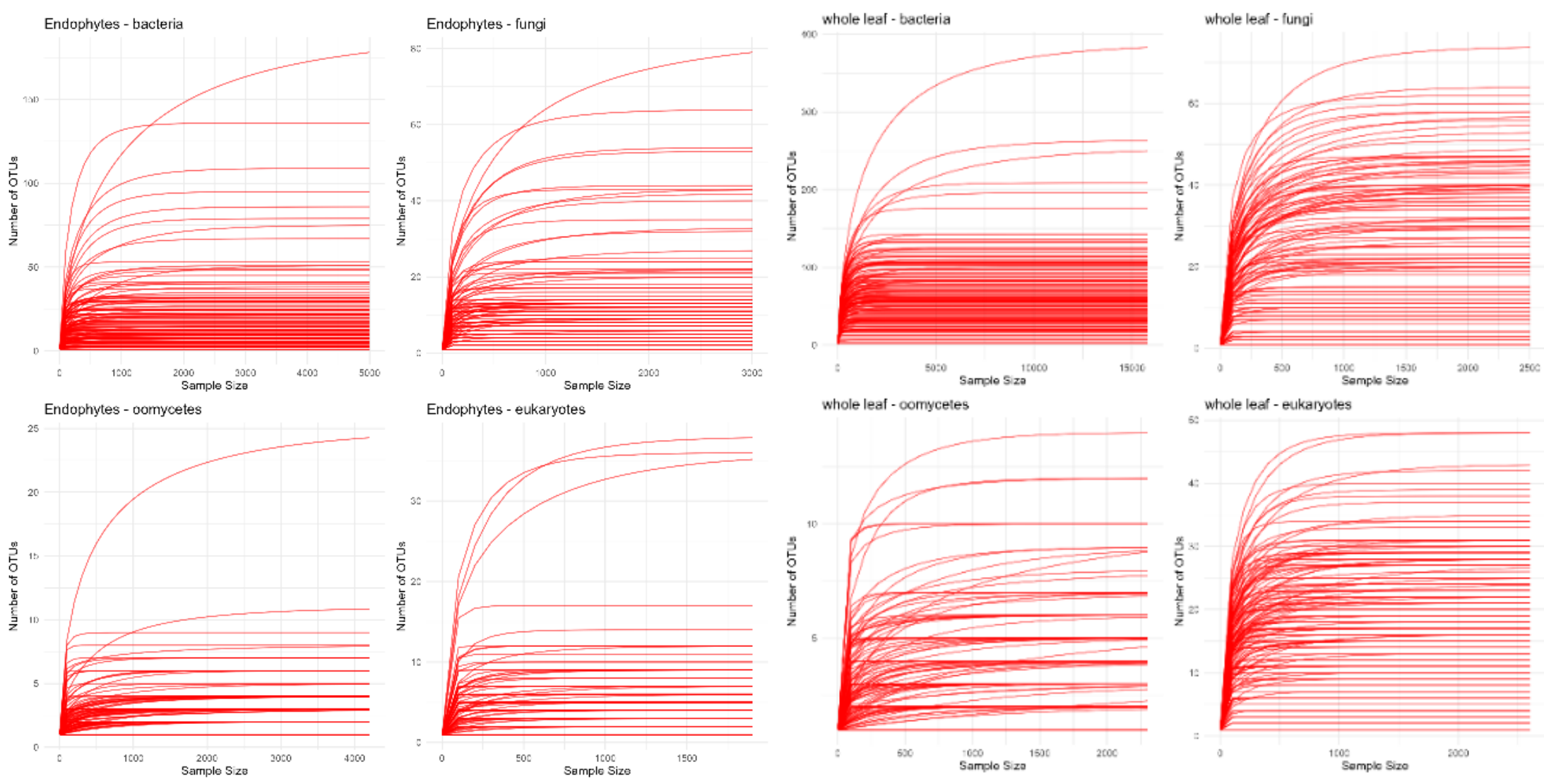

Figure 5B: Would it be possible to verify the abundance of other microbial strain (e.g. to verify that an increase of other microbes caused a decline in the population of Dioszegia?

We thank the reviewer for this interesting question. In this experiment, the initial inoculum of *Dioszegia* used under holoxenic conditions was likely higher than what occurs during natural colonization (e.g., during seed germination), as our primary goal was to experimentally reproduce and observe the *Albugo-Dioszegia* interaction *in planta*. In this context, a decline in the *Dioszegia* population is expected as the yeast immediately faces intense competitive exclusion from the resident microbiota. This is further supported by our gnotobiotic data (Extended Data Fig. 9b),

which shows that even in a simplified system, *Albugo* itself can restrict *Dioszegia* growth, likely through niche or nutrient competition.

In the future, it would be highly valuable to characterize the metabolic profiles of such yeasts within complex microbial networks to better predict competition and the specific mechanisms of exclusion. However, we consider this a distinct ecological question that falls outside the primary scope of our current study on thiamine cross-feeding. We have revised the second paragraph of the Discussion (Lines 488-502) to include a brief discussion of these competitive dynamics and the "ecological rescue" provided by *Albugo*.

Reviewer #4 (Remarks to the Author):

Key results
This study identifies a conserved metabolic mutualism between the basidiomycete yeast Dioszegia hungarica and the oomycete Albugo laibachii within the Arabidopsis thaliana leaf microbiome. Using a continental-scale survey combined with targeted co-colonization and genetic assays, the authors provide compelling evidence that thiamine cross-feeding underpins the stable coexistence between these two dominant hub taxa. A central and notable finding is that Dioszegia produces and secretes excess thiamine, which directly facilitates Albugo colonisation, thereby promoting microbiome stability. This work convincingly links metabolic dependency to ecological prevalence and suggests that nutrient exchange is a key organising principle of leaf microbiomes.

We thank the reviewer for the nice summary and positive evaluation of our manuscript.

Validity
Overall, the data interpretation is sound, and the conclusions are well supported by the experimental evidence. The authors used appropriate controls throughout, and the logic connecting thiamine biosynthesis, secretion, and mutualistic interactions is clear. I did not identify any flaws that would prevent publication. Some interpretations would benefit from clarification, particularly regarding the apparent persistence of Dioszegia strains lacking DhPER1 during long-term cocultivation, despite the demonstrated importance of this gene for Albugo colonisation. This issue can be resolved through a clearer explanation rather than additional experimentation.

We thank the reviewer for this insightful comment. We have now expanded our discussion to emphasize the asymmetric nature of this interaction: While *DhPER1* is essential for the yeast to promote *Albugo* infection, the return benefit from the pathogen to the yeast is likely indirect. As a "niche engineer," *Albugo* modifies the host environment—likely through immune suppression, microbial antagonism, or modulation of host metabolites—to provide an advantageous environment for its microbial partners.

Line 493-498: While *Dioszegia* alone faces competitive exclusion by the native microbiota (Fig. 5b), the presence of *Albugo* enables its long-term persistence (Fig. 5c). This 'ecological rescue' suggests that the benefit provided by *Albugo* to *Dioszegia* is primarily indirect, where *Albugo* acts as a niche engineer that modifies the leaf environment—potentially through immune suppression[54], microbial antagonism[22], or modulation of host metabolites exuded during infection[60,61]—to shelter its metabolic partner.

Significance
The conclusions are significant for the plant microbiome field and extend beyond it to microbial ecology and host-associated microbiomes. This study provides rare mechanistic evidence for how metabolic mutualism shapes community structure in natural plant systems. The identification of vitamin cross-feeding as a driver of hub taxon stability has clear implications for microbiome engineering and plant resilience strategies. While previous work has shown that Albugo suppresses host defence and thereby facilitates secondary pathogen colonisation (https://doi.org/10.1094/MPMI-21-6-0745), this manuscript adds a complementary microbial metabolic dimension that strengthens, rather than conflicts with, existing literature. Together, these findings elevate the conceptual importance of this work.

We thank the reviewer for the positive comment and for pointing out the excellent study about *Albugo* suppressing host defence. We have now added this work in the discussion:

Line 474-477: Combined with previous work showing that *A. laibachii* creates niches by suppressing plant immunity and inhibiting the proliferation of certain microbes within the microbiome[22,54], we propose that the *Albugo*-centered interaction network is a fundamental component of *A. thaliana* leaf microbiome assembly and stability.

Data and methodology
The experimental approach was appropriate and well executed. The continental survey was carefully designed and provided a strong ecological foundation for the subsequent mechanistic work. The genetic, physiological, and colonisation assays were of high quality, and the data were presented clearly in both the main figures and the supplementary materials. The manuscript included relevant controls and sufficient replication. The overall presentation allowed the reader to follow the progression from field observations to molecular mechanisms without ambiguity.
We thank the reviewer for the careful evaluation of our experimental approach and the kind words. We are delighted that the reviewer found the presentation clear and easy to follow from field observations to molecular mechanisms.

Analytical approach
The analytical framework is robust and suitable for the questions addressed. The statistical analyses appear appropriate and are applied consistently across datasets. The integration of ecological association data with functional genetic analysis is a particular strength. No aspect of the analytical approach falls outside my expertise, and I did not identify omissions or inappropriate statistical treatment that would undermine the conclusions.
We thank the reviewer for the thorough assessment of our analytical framework and are pleased that the integration of ecological and functional genetic analyses was recognized as a strength of the study.

Suggested improvements
All suggested improvements fall well within the current scope of the manuscript. First, the rationale for selecting specific thiamine-related genes, particularly those discussed in lines 212–214, should be explained more explicitly. Second, gene nomenclature requires clarification at the first mention, especially for DhPER1, to avoid confusion with unrelated genes in other systems (PER1 refers to a core circadian clock gene in other organisms). Third, the discussion would benefit from modest expansion to address ecological implications more fully, including how thiamine cross-feeding might

interact with Albugo-mediated suppression of plant immunity and broader pathogen dynamics. No additional experiments are strictly necessary.

We thank the reviewer for these important suggestions. We have now revised all three points as follows:

1. We have explicitly stated the rationale for focusing on thiamine-related genes:

Line 225-229: We next generated draft genomes of *Dioszegia* EY and PT strains using a recent reference[37] (Supplementary Table 3) and compared all their thiamine related genes. We focused on this biosynthetic pathway because it is absent in the *Albugo* genome, making thiamine a primary candidate for metabolic complementation in the nutrient-limited phyllosphere.

2. We have clarified the gene nomenclature for *DhPER1* at the first mention:

Line 232-235: We observed that one gene, *Dioszegia hungarica* PERMEASE 1 (*DhPER1*), has a high density of SNPs (105.7 SNPs/kb) relative to the other thiamine-related genes in EY and PT genomes (Fig. 2b), nearly doubled that of the second highest genes.

3. We have expanded the discussion to mention the potential relationship between thiamine crossfeeding and the *Albugo*-mediated suppression of plant immunity:

Line 492-502: However, in a natural ecological context (holoxenic conditions), the relationship shifts toward facilitation. While *Dioszegia* alone faces competitive exclusion by the native microbiota (Fig. 5b), the presence of *Albugo* enables its long-term persistence (Fig. 5c). This 'ecological rescue' suggests that the benefit provided by *Albugo* to *Dioszegia* is primarily indirect, where *Albugo* acts as a niche engineer that modifies the leaf environment—potentially through immune suppression[54], microbial antagonism[22], or modulation of host metabolites exuded during infection[60,61]—to shelter its metabolic partner. The provision of thiamine by *Dioszegia* might fuel the metabolic demands of *Albugo* during these energy-intensive processes of host colonization and immune manipulation. This asymmetric mutualism, combining direct metabolic supply with indirect niche stabilization, indicates that the interaction network extends beyond a single gene-loss dependency and likely involves multiple microbial partners and layered ecological functions.

Clarity and context
The manuscript is clearly written, logically organised, and accessible to a broad readership in plant microbiome research. The figures and tables are well chosen and effectively support the narrative. The contextualisation of the results is generally strong; however, the discussion would benefit from a slightly broader framing that includes obligate downy mildew and related Arabidopsis pathogens, which would reinforce the relevance of the findings. These are issues of emphasis rather than clarity.

We thank the reviewer for this suggestion. We have now further expanded the last paragraph of the Discussion and included the implications of our findings for the broader context of downy mildew and other obligate microbes with corresponding citations:

Line 508-510: However, in plant-associated obligate microbes, such as downy mildews[66] (*Hyaloperonospora* spp.), rusts[67] (*Puccinia* spp.) and arbuscular mycorrhizal fungi[68], nutrient auxotrophy is primarily regarded as a host-dependent trait, despite their central roles in both leaf and root microbiomes.

References
The manuscript appropriately cites the relevant literature and places the findings within the existing

body of work. With the inclusion of a brief discussion of prior studies on Albugo-mediated immune suppression, the reference framework would be fully balanced and comprehensive.

We thank the reviewer for the positive assessment of our reference framework. As noted in our response to the Significance comment above, we have already incorporated a discussion of *Albugo*-mediated immune suppression in the revised manuscript (Line 470–473, Line 493), which we believe addresses this suggestion.

Your expertise
My specialization include plant microbiomes, microbial ecology, and interactions between hosts and microbes, which includes designing experiments, interpreting data, and drawing biological conclusions. I did not thoroughly evaluate the underlying computational code or its implementation, as this is beyond my expertise. Consequently, my assessment of the analyses is confined to their conceptual soundness, biological interpretation and alignment with the results presented.

We thank the reviewer again for the transparent disclosure of their expertise and for the thorough and professional evaluation of our manuscript.

Reviewer #1 (Remarks to the Author):

I appreciate the detailed work the authors put into responding to the reviewers' concerns. I believe all of my questions and concerns have been addressed.

We sincerely thank the reviewer for the positive assessment and are glad that all questions and concerns have been fully addressed.

Reviewer #2 (Remarks to the Author):

The authors have addressed my concerns satisfactorily. In particular, the new ELISA data substantially strengthen the proposed thiamine-export mechanism, and the revised framing of the Dioszegia–Albugo relationship as context-dependent is more consistent with the data. The additional experiments for Figure 5C and the revised statistical treatment of this data also addresses my concerns and reveals a highly significant result consistent with the earlier figures. A few requested analyses were not performed exactly as suggested, especially the microbial-DNA normalization and the quantitative permutation-based treatment of Fig. 1F, but the authors provide good explanations and have appropriately clarified the associated claims.

Minor comments
Line 243: "b, Scatter plot" Change to 'Bar plot'.

We apologize for this mistake. It has been fixed now.

Line 176: "Dioszegia and Leucosporidium displayed outstanding betweenness centrality among all OTUs". You might consider also labeling Leucosporidium in Figure 1d, especially considering it is the only other species that has a significant effect on Albugo infectivity (Figure 1e).

This is a good point. *Leucosporidium* is now labelled in Figure 1d.

We sincerely thank the reviewer for all helpful suggestions, especially for recognizing that the new ELISA data strengthen the thiamine-export mechanism and that the framing and claims have been appropriately clarified.

Reviewer #3 (Remarks to the Author):

The reviewers properly addressed most of my comments (reviewr 3). In their response, it is stated that it cannot be excluded that the yeast simply benefits from plant metabolites that are exuded upon Albugo infection of the plant (so an indirect benefit).

The following answer is given by the authors: We agree with the reviewer that this is a very

plausible mechanism and cannot be excluded. The 'modulation of host metabolites' is likely a key component of the indirect benefit Albugo provides to Dioszegia. We have now explicitly incorporated the 'host exudate' hypothesis into our revised Discussion (Paragraph 2) in our response to point (2) above.

This is okay with me. However, the abstract for instance sounds very conclusive. It states: We show that Dioszegia facilitates Albugo colonization by supplying thiamine via a dedicated membrane permease, alleviating Albugo’s auxotrophy. Genomic and transcriptomic analyses reveal that natural selection has acted on thiamine production in Dioszegia,shaping this metabolic complementation.

If the authors are not sure, it is better to soften the conclusions a bit.

We thank the reviewer for this comment and agree that a more balanced tone is better for the less certain claims. We have softened the abstract accordingly: “reveal” has been changed to “indicate”, and the *in planta* persistence statement is now qualified as observed “under glasshouse conditions”.

We also thank the reviewer for all constructive suggestions, which improved our manuscript significantly.

Reviewer #4 (Remarks to the Author):

I thank the authors for their thorough and careful responses to my comments. All points have been addressed to my satisfaction. The revisions, particularly the expanded discussion on the asymmetric nature of the Dioszegia-Albugo interaction, the clarification of DhPER1 nomenclature, and the broader framing of ecological implications, have strengthened the manuscript. I have no further comments.

Mahmut Tor

We sincerely thank Professor Tör for the positive assessment and are pleased that all points have been addressed to his satisfaction.
